# Curated character of the Initial Upper Palaeolithic lithic artefact assemblages in Bacho Kiro Cave (Bulgaria)

**DOI:** 10.1371/journal.pone.0307435

**Published:** 2024-09-04

**Authors:** Tsenka Tsanova, Vincent Delvigne, Svoboda Sirakova, Elka Anastasova, Pedro Horta, Ivaylo Krumov, João Marreiros, Elena Nacheva, Zeljko Rezek, Jean-Jacques Hublin, Nikolay Sirakov

**Affiliations:** 1 Department of Chemistry G. Ciamician, Alma Mater Studiorum, University of Bologna, Bologna, Italy; 2 Department of Human Origins, Max Planck Institute for Evolutionary Anthropology, Leipzig, Germany; 3 CNRS, UMR 8068 TEMPS, University of Paris X—Nanterre, Nanterre, France; 4 Service de Préhistoire, University of Liège, Liège, Belgium; 5 National Institute of Archaeology with Museum, Bulgarian Academy of Sciences, Sofia, Bulgaria; 6 Department of History, University of Minho, Braga, Portugal; 7 Interdisciplinary Center for Archaeology and the Evolution of Human Behaviour, Universidade do Algarve, Campus de Gambelas, Faro, Portugal; 8 Museum of History- Belogradchik, Belogradchik, Bulgaria; 9 TraCEr, Monrepos Archaeological Research Centre and Museum for Human Behavioural Evolution, LEIZA, Mainz, Germany; 10 Sofia University “St. Kliment Ohridski, Sofia, Bulgaria; 11 Chaire de Paléoanthropologie, CIRB, Collège de France, Université PSL, CNRS, INSERM, Paris, France; Griffith University, AUSTRALIA

## Abstract

The dispersal of *Homo sapiens* across Eurasia during MIS 3 in the Late Pleistocene is marked by technological shifts and other behavioral changes, known in the archaeological record under the term of Initial Upper Paleolithic (IUP). Bacho Kiro Cave in north Bulgaria, re-excavated by us from 2015 to 2021, is one of the reference sites for this phenomenon. The newly excavated lithic assemblages dated by radiocarbon between 45,040 and 43,280 cal BP and attributed to *Homo sapiens* encompass more than two thousand lithic artifacts. The lithics, primarily from Layer N1-I, exist amid diverse fauna remains, human fossils, pierced animal teeth pendants, and sediment with high organic content. This article focuses on the technological aspects of the IUP lithics, covering raw material origin and use-life, blank production, on-site knapping activities, re-flaking of lithic implements, and the state of retouched lithic components. We apply petrography for the identification of silicites and other used stones. We employ *chaîne opératoire* and reduction sequence approaches to profile the lithics techno-typologically and explore the lithic economy, particularly blade production methods, knapping techniques, and artifact curation. Raw material analysis reveals Lower Cretaceous flints from Ludogorie and Upper Cretaceous flints from the Danube region, up to 190 km and 130 km, respectively, from Bacho Kiro Cave, indicating long-distance mobility and finished products transport. Imported lithic implements, were a result of unidirectional and bidirectional non-Levallois laminar technology, likely of volumetric concept. Systematic on-anvil techniques (bipolar knapping) and tool segmentation indicate re-flaking and reshaping of lithic implements, reflecting on-site curation and multifaceted lithic economy. A limited comparison with other IUP sites reveals certain shared features and also regional variations. Bacho Kiro Cave significantly contributes to understanding the technological and behavioral evolution of early *Homo sapiens* in western Eurasia.

## Introduction

The area between the Balkan Mountains and the lower Danube valley lies on the potential departure point of the “Danube corridor”, proposed to be a major route for human dispersals during the Paleolithic and the spread of Upper Paleolithic *H*. *sapiens* into Europe [1–5]. This makes the region crucial for investigating the expansion of our specie across Eurasia. In Europe, this event occurred between 50 ka and 45 ka [6, 7], with *H*. *sapiens* dispersing into new territories and continuing to interact with and, in some cases, replace existing Neanderthal groups. This demographic and cultural process left clear imprint in the archaeological record, in the so-called Initial Upper Paleolithic (IUP) material record characterized by advanced technological practices, such as blade technology with Levallois related attributes and a variety of finely crafted bone tools and personal ornaments. The IUP was first defined after the uppermost deposit (Level 4) at Boker Tachtit in the Negev Desert [8]. Due to the demonstration of its direct development from the technology of the Emirian of the lower layers (layers 1–3) at the same site [9], the term IUP has been expanded first to include the Emirian [10], and then, more as a chrono-stratigraphic unit, to the record from across Eurasia that occurs after the local clear Middle Paleolithic and contains a variety of Levallois-related attributes and concepts of blade production [11]. In southeast Europe, caves on the northern slopes of the Balkan Mountains such as Bacho Kiro Cave, Temnata, and Kozarnika [12], bear witness to the demographic, technological and adaptive processes, of the IUP groups of *Homo sapiens*. Bacho Kiro Cave, as one of the last excavated, represents the IUP of the finest resolution and most complete material culture representation in this part of Europe. The IUP deposits yielded more than four bone fragments and one molar deriving from three human individuals [13] in a well-defined stratigraphic context, with rich and preserved fauna, lithics, bone tools, and personal ornaments [14]. The individuals recovered by aDNA had Neanderthal ancestors a few generations back, confirming that the first UP European *H*. *sapiens* interacted with Neanderthals [13].

The IUP is primarily recognized and defined as a techno-complex [11] stratigraphically always following the Middle Paleolithic and preceding the Early Upper Paleolithic (EUP) techno-complex with emphasis on bladelet production (Protoaurignacian, Early Kozarnikan etc.). Archaeological and biological pieces of evidence strongly suggest that the relatively rapid dispersal of IUP from southwest Asia [15] into mid-latitude Eurasia was by *H*. *sapiens* groups genetically unrelated to the subsequent EUP populations (Aurignacian) and present-day European populations [13, 16]. These first expansions in Europe may have begun as early as 48 ka cal BP [17] as suggested by the record from Ranis [18] but also from isolated human fossil finds likely related to the IUP Bohunician [7]. The record from Bacho Kiro Cave (Layers I and J) aligns with the chronological and the techno-cultural distribution of the IUP laminar assemblages, and from biological perspectives [13, 14] completely fits in the emerging scenarios of *H*. *sapiens* large-scale dispersals and trajectories of cultural evolution [11, 19].

The Eurasian IUP presents several global challenges and questions about chronology, expansion patterns, cultural diversity, adaptation strategies, and interaction with Neanderthals. Reconstructing the time and duration of the IUP across Eurasia is a key but a perplexing task due not only to different dating methods but also to the recognition of valid stratigraphic units [20], scarcity of human fossils [17] and state of preservation of the dating materials [21]. From a chrono-stratigraphical and lithic technological perspective, identifying the IUP today may not be overly challenging [11, 22] however it can be confusing in light of the emerging diversity of the IUP record and when we have only partially aligning data with the IUP definition [11]. An ongoing debate how should be the IUP term used it as a chronological marker or as a techno-complex (i.e. an aggregation of cultures sharing some techno-typological traits) and how this relates to the Neanderthal-*H*. *sapiens* turnover [21, 23–27], and to the replacement of Denisovans [28] in Asia. Under discussion is that the IUP may not exclusively signify the dispersal of *H*. *sapiens* but in certain regions could also stem from convergent evolution or phylogenetic factors [29], aligning with the multiregional (polycentric) model [30]. In the discourse on early *H*. *sapiens* dispersal in Eurasia during MIS 3, the emergence of the IUP techno-complex stands as a pivotal phenomenon reflecting the local adaptation and evolution. In Bacho Kiro Cave, the integration of highly precise radiocarbon chronology, genetic analysis of human fossils, coupled with extensive collections of faunal remains and lithic and osseous industries, alongside comprehensive data on climate, environmental conditions, and raw material origins, collectively unveil a compelling assessment: the emergence of the IUP entity, demonstrating synchronous adaptation within the region after interacting with Neanderthals and before dispersing into Asia [13]. The recently excavated site of Bacho Kiro Cave can provide valuable insights into global challenges by shedding light on the outcomes of IUP *H*. *sapiens* dispersals, as well as their methods of organization and adaptation to environmental conditions and natural resources. Overall, the study of raw material procurement and lithic technology aspects of the IUP provides a multidimensional perspective on the dispersal of *H*. *sapiens* in Eurasia, offering valuable insights into their mobility, environmental adaptation, cultural dynamics, and behavioral ecology during this pivotal period in human prehistory.

In this paper, our aim is to enhance understanding of the integrity of technical systems [31], presented in the IUP lithic assemblages from layers I and J in Bacho Kiro Cave. We examine the organization of activities, including raw material acquisition and off- and on-site activities, to reconstruct lithic technology and the curated character of the lithic implements imported as finished products in the cave. Additionally, we compare the IUP lithic technology with that of other sites and discuss the implications of our findings for the early dispersal of *H*. *sapiens*, integrating genetic, environmental, and the available interdisciplinary data. We explore regional techno-cultural variability and gain insight into the techno-cultural, and behavioral strategies employed by early *H*. *sapiens* in the region.

### Background to Bacho Kiro Cave: Stratigraphy, chronology and cultural interpretations

**Fig 1:**
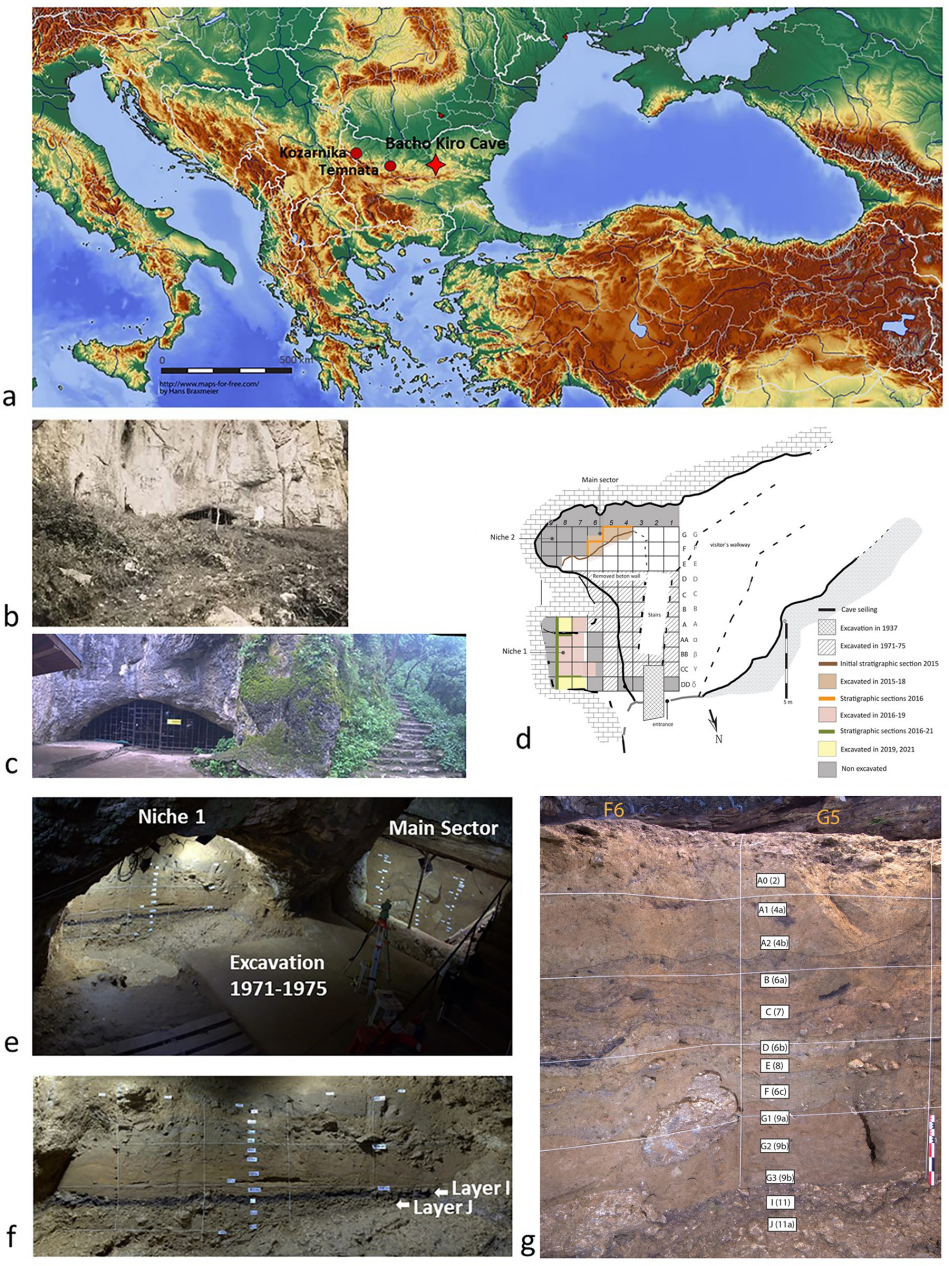
Location of the cave and the excavated area. a. Map with location of Bacho Kiro Cave, and the mentioned Temnata and Kozarnika caves (T. Tsanova); b. The cave entrance in 1938 (R. Popov and D. Garrod excavation); c. The entrance of Bacho Kiro Cave today (V. Aldeias); d. Site plan and excavation grid showing previous excavations and excavated areas from 2015 to 2021 (V. Aldeias, modified T. Tsanova); e. View to the excavation sectors (Sh. McPherron); f. Niche 1 with location of layers I and J (Sh. McPherron); g. The Main Sector initial stratigraphic section in 2015 with indicated layers and the corresponding layers from 1971–1975 excavations (T. Tsanova).

The Bacho Kiro Cave (42°56′48″N; 25°25′49″E), is situated on the northern range of the central Balkan Mountains near the town of Dryanovo in Bulgaria. The cave is located about 70 km south of the Danube River and 260 km west of the Black Sea **(Fig 1A)**. Its archaeological deposits formed at the mouth of a large Cretaceous limestone system that is over 3 km long and lining up along the Dryanovo River canyon.

**Fig 2:**
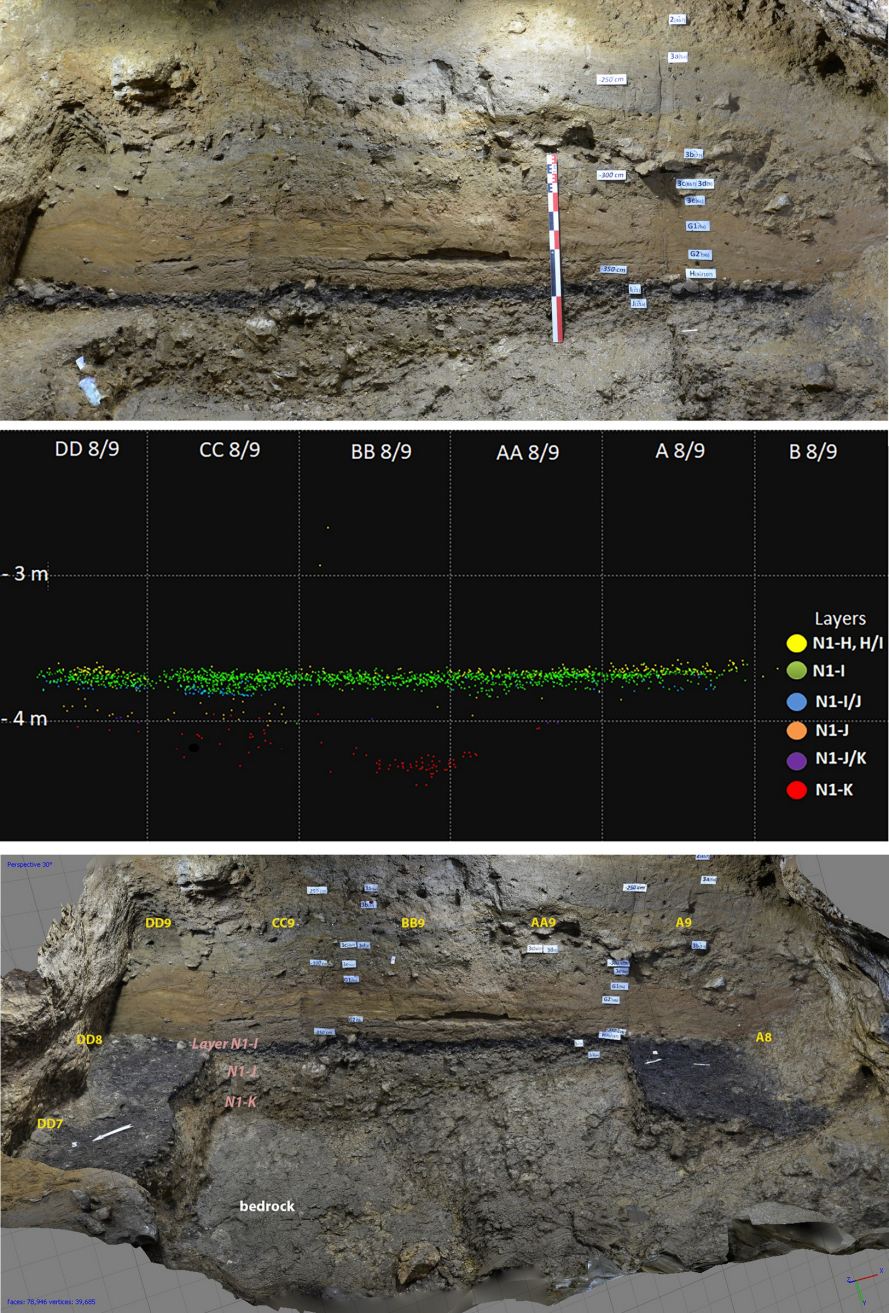
Stratigraphy and lithics plot in Niche 1, Bacho Kiro Cave. Top: Picture of the stratigraphic section (East) (T. Tsanova); Middle: Plot of the lithics per layer (T. Tsanova & Z. Rezek); Down: 2019 excavations in sq. A8, DD7, and DD8 with the distinctive dark-gray sediments of Layer I (3D model N. Zahariev in Sirakov, Tsanova, Hublin 2019).

The research on the Upper Paleolithic (UP) and the “Aurignacian” *Homo sapiens* in Bacho Kiro Cave began with the investigation by R. Popov and D. Garrod in 1930s **(Fig 1B)**. The archeological attribution of the excavated material was only of general character [32]. In the 1970`s new field investigations were established by a Bulgarian-Polish team led by B. Ginter and J.K. Kozlowski which excavated the area of about 58 m^2^ [33]. They established archeological sequence spanning 5 m in depth that covers deposits from the Middle Paleolithic (MP) and the UP with a clear technological break between them [34]. At the base of the sequence three Mousterian layers (layers 14, 13 and 12) attested the use of local (available less than 10 km from the site) stone raw materials and Levallois method for flake production [35]. The overlying UP deposits encompassed very early UP in layers 11 and 11a, with succeeding Aurignacian, and Epigravettian at the top of the sequence (layer 2) [33]. The layer 11 is the richest in finds **(Fig 2B)**. The collection from this layer became one of the references for the earliest UP in Europe, even at times being interpreted as the possible origin of the Aurignacian on the basis of tool typology and presence of pendants and ornaments that were assumed to be made by *Homo sapiens* [1, 33]. The first radiocarbon date (^14^C) for layer 11 resulted in an age of > 43 ky BP (GrN 7545) attesting to older, pre-Aurignacian, age of this UP record [33]. In general, the identified techno-typological and raw material differences between the MP and UP deposits have been interpreted to reflect the use of this landscape by a different group(s), which led to the regional models of the replacement of Neanderthals by *H*. *sapiens* populations [33, 36]. Subsequently, a detailed analysis of the lithic collections from layers 11 and 11a (today Layers I and J, respectively) demonstrated that artifacts share certain technological features with those of underlying MP (e.g., thick facetted platforms, occasional bidirectional flaking by a direct hard-hammer percussion as developed in [37, 38].

Therefore, the name of “Bachokirian” [33] emerged for these collections, being classified as a transitional Middle-to-Upper Paleolithic industry, not as resulting from mixed layers but rather as reflecting a behavioral continuity from techno-typological and economical perspective between the two periods [37]. We started new excavations in 2015 as a joint project of the National Archaeological Institute with Museum of the Bulgarian Academy of Sciences [39] and the Department of Human Evolution at the Max Planck Institute for Evolutionary Anthropology in Leipzig (Germany). Our excavation was directed towards two areas adjacent to the old excavation area: the “Main Sector” (ca. 3 m^2^) and the “Niche 1” (more than 10 m^2^) which is a low-ceilinged lateral chamber **(Fig 1D, 1E)**. The UP sequence is best preserved in the Main Sector **(Fig 1G)** where we correlated the UP layers with the previously reported stratigraphy [14]. Below the UP deposits, due to the raised cave floor in this sector, only the upper part of the Layer J (previous 11a) is present **(Fig 1G)**. In the Niche 1 the cave floor is at a lower elevation (since it slopes towards the current exit of the cave). The lower part of the overall stratigraphy, including the MP and IUP deposits, is thus substantially developed across this area **(Figs 1F and 2)**. The newly recognized layers are kept separate from the two sectors with the layers from Niche 1 prefixed as “N1-” and letters in order to differentiate with previous excavation where the layers were indicated only with numbers. We correlated our stratigraphy with the previous one first by our field observations while excavating and then with the extensive set of radiocarbon dates of high precision [6]. The IUP layers from the Niche 1 and the Main Sector are also associated with genetic refits of two human fragments identified by ZooMS and belonging to either the same individual or identical twins [13]. A human molar F6-620 found in the upper part of Layer J in the Main Sector and a bone fragment AA7-738 found in Layer I in the Niche 1 have identical mitochondrial genome sequence suggesting to belong likely to the same male individual [13].

The present stratigraphy at the bottom starts with Layer K, which includes sedimentary deposits that accumulated during the late MP [34]. This layer is sloping towards the cave entrance that was probably blocked by roof fall allowing for the accumulation of these sediments over the inclined bedrock [14]. The Layer K is dated by OSL to about 61 ka, but this age should probably be taken as a maximum due to the sediments of this layer coming and being reworked from inside the cave. ^14^C age brackets its age above 50 ky BP [40]. The switch to the IUP seems to be in the lower part of Layer J. This break is not sharp, either in terms of geology or the accumulation of artifacts [6]. The sedimentary contact between Layer J and the underlaying Layer K is generally gradual and in the lower part of Layer J there are artefacts from fine-grained flint, which is characteristic for the lithics of the upper Layer J and the Layer I, and artefacts of local raw material with predominantly Levallois technology (alike in the Layer K). Difference in technology and raw material detected in the upper part of J, together with the presence of the human molar (F6-620, mentioned above) found 8 cm below the contact with Layer I in the Main sector, attest to the clear IUP occupation which continues into Layer I. Layer K, J and I in the Niche 1 are rich in limestone clasts up to 20 cm from roof fall in Layer K and J [14] and also in the three layers clasts with sizes 1–2 cm and 4–8 cm [41] and associated with dry cave setting with main source of sediment originating from inside the cave [14]. Unlike contact between the Layers K and J, the contact between the Layers J and I is sharp due to the distinctive nature of Layer I sediments. The latter is a distinctive dark brown loamy clay and it is the richest layer in archaeological remains within the whole sequence **(Fig 2, S1 Table)**. The Layer I deposit is an aggregate of anthropogenically accumulated materials such as bone bits bellow 20 mm in size, knapped flint fragments (bellow 15 mm), charcoal, and plant remains. The dark color is due to these organic inputs [14]. In our excavation no distinctive combustion feature was found, which is in contrast with the previous excavations that took place in the center of the chamber where they reported on thirteen hearths in four different stratigraphic units. In our excavations in the Niche 1, the deposits represent a mix of burned and unburned components, possibly resulting also from raking the material closer to the cave wall. Micromorphological analysis of Layers I and J reveals sporadically crudely bedded lenses of sands and silts, approximately 1.5–2 mm in thickness, which suggests there were several surfaces formed during the formation of this layer that were impacted by low-energy sheet wash [14]. These observations are indicative of reoccupation events and arguably an intensive use of this cave in general during the later part of the IUP occupation of the cave. The Layer I is capped by water-laid sediments related to a low-energy stream from inside the karst system [14].

A large set of radiocarbon dates with exceptionally narrow error ranges places the upper part of Layer J at 45 990 cal BP and Layer I between 45 040 to 43 280 cal BP [6, 42] **S1 Appendix in S1 File**.

In order to present the IUP character of the Bacho Kiro assemblage of Layer I, both in the Main Sector and Niche 1, here we present four main aspects of the lithic record: raw material use, blank production technology (especially in regards to blades), on-site lithic curation activities (blade and tool fragmentation, reshaping and re-flaking of tools, on-anvil flaking), and typology of the retouched artifacts. We then briefly discuss this chrono-cultural (to techno-complex) attribution within the context of other regional and Eurasian IUP contexts.

## Materials and methods

The analyzed lithic collection attributed to IUP consists of 2247 artifacts, which represents 92.7% of the excavated piece-plotted lithics from the IUP deposits **(Table 1)**. The majority of the lithics originate from the Layer N1-I (n = 1716), and the number from the overlying layer N1-H (including the contact N1-H/I) is 305. The contact layer N1-I/J yielded 102 lithics, while Layer N1-J only 41. The stratigraphic context of artefacts origin is described further on.

**Table 1:** Quantities and percentages of analyzed lithics that were individually provenienced, total number of individually provenienced lithics in the IUP layers per sector, and layers of excavation in Bacho Kiro Cave, excavation 2015–2021.

	IUP Layers	Analysed plotted (> 1.5 cm)	Not analyzed	Total plotted Main DB	Analysed from sieving (<1.5 cm)
**Main Sector**	H	1	0	1	1
	H/I	13	0	13	0
	I	34	8	42	0
	I/J	25	1	26	23
	J	9	1	10	0
**Niche 1**	N1-H	51	42	93	187
	N1-H/I	251	0	251	99
	N1-I	1716	92	1808	141
	N1-I/J	106	21	127	24
	N1-J	41	12	53	4
	Total	2247	177	**2424**	480
	**%**	**92.7**	**7.3**	**100**	Ongoing

### Excavation methods

The site was excavated following established protocols described in [14]. Layers were defined based on lithological and archaeological criteria, with stratigraphy independently named from previous excavation [34]. Two separate areas were excavated: the south area, known as the Main sector, and a niche to the east called Niche 1 (N1) **(Fig 1D, 1E)**. Layer naming conventions differ between the two areas. All lithics >15 mm and fauna >20 mm in length and all samples (ancient DNA, micromorphology, phytoliths etc.) were provenienced using Leica total station (5″ accuracy) and given unique identifiers (IDs). Sediments were collected in 9-liter buckets and wet-screened on-site through 6- and 1.2-mm meshes. Buckets were assigned unique IDs and coordinates measured before (on the field) and after excavation (in the lab). All features were provenienced, and digital photography documented daily. Final sections were documented through photography, drawings, and total station measures, with structure-from-motion models created and georeferenced.

The excavation of Bacho Kiro Cave was authorized by the Bulgarian Ministry of the culture, delivered by National Institute of Archaeology with Museum at Bulgarian Academy of Sciences, Sofia (NIAM-BAS): Nr 124/11.05 2015; Nr 225/28.04.2016; Nr 47/02.05.2017; Nr 99/17.04.2018; Nr 120/2019, and Nr 252/2021.

### Raw material analysis

Each sample (neither artifact of geological reference) is described according to three types of observation that document particular aspects of their life: diagenesis, gitological alteration/deposition, and post-depositional modification [43–46].

The objective of this three-tiered approach to raw material characterization is reconstruction of the pre- and post-depositional history of lithic objects as well as the litho-space of layer [47].

First set of variables describes the diagenesis processes the environment of formation and the age of the rock that make it possible to characterize the “genetic type” [46] **S2 Appendix in S1 File**. We mainly focused on distribution, abundance, sorting, bluntness, sphericity, size, and nature (detritic, chimical or biogenic) of clasts [48] using binocular analysis. Objects are analyzed under a film of water with a magnification between x80 and x200. In this respect, we take into account all the variables to definie the microfacies [49] and do not base the diagnosis of silicite groups on one variable only **S3 Appendix in S1 File**.

The second set of variables aims to identified the gitological alteration and deposition. For each surface, we deciphered the kinds of physical actions (such as cracking or fragmentation), as well as the chemical processes (such as alterations or diffusion of oxides in the matrix) that had acted on or from that surface. In the end, we obtained indicators for a type of deposition [50] primary, sub-primary, colluvial, alluvial, old alluvial, marine, etc.).

Finally, the third set of variables concerns taphonomy. This describes the kind and intensity of post-depositional processes according to the microtopography: such as patina, shocks, glosses, thermal phenomena. These elements give us indications of the edaphic processes that the objects were exposed to since their discard. Detailed description of the methodology for raw material analysis and the criteria of definition for each group of silicite rock is given in **S4 Appendix in S1 File**.

### Lithic analysis

We performed techno-typological and techno-economic analysis of lithic artifacts using a *chaîne opératoire* approach [47, 51–53]. Stone tool curation refers to the process of managing and maintaining stone tools throughout their life cycle [54–57]. Description of the methods of lithic analysis are given in S5 Appendix in **S1 File**.

## Results

### Stratigraphic context

The stratigraphy in both sectors consists of mainly UP deposits, from layers 3c/3d at the top to J at the bottom **(Fig 1F, 1G)**. The MP is present only in Layer K, which accumulated only in the Niche 1 **(S1 Table)**. The Main Sector (MS) had 246 piece-plotted (cut-off size 1.5 cm) lithics across ca. 3m^2^, for 297 liters of sediments, while Niche 1 yielded 2435 lithics across ca. 10 m^2^ for 4392 liters of sediments. At times, lithics smaller than 1.5 cm, including small bipolar flakes or well-shaped tiny flakes and bladelets, were also individually plotted during the excavation. The majority of them are from the IUP deposits from Layer N1-H, to Layer N1-J. In our analysis, we merged the collections from these two sectors, and henceforth we will refer to these without the sector prefix (as Layer I, J). When we refer to these collections only in the Niche 1, we will refer to them with the “N1” prefix.

The density and total number of finds in the IUP layers (for both sectors merged). The density of lithics is the lowest in the lowermost IUP Layer N1-J, only 0.03 per liter of excavated sediment while in Layer N1-I the density is 2.03 per liter of sediment **(Table 2)**.

**Table 2:** Number and density of plotted lithic (>15 mm) and faunal remains (>20 mm) in the IUP layers (Main Sector and Niche 1 merged) in Bacho Kiro Cave.

Layers	Attribution	Lithic	Bones	N* buckets**	Volume (m3)	Liters	Lithic/l	Bone/l
**H, H/I**	IUP	357	895	122	1.098	1098	0.32	0.81
**I**	IUP	1851	12.713	101	0.909	909	2.03	13.98
**I/J**	IUP	153	1567	43	0.387	387	0.39	4.04
**J**	IUP	63	2042	222	1.998	1998	0.03	1.02

* Abbreviations: N = number; lithic, bone/l = number of lithic, bone per liter of sediment

**: 1 bucket consists of 9 liters of excavated sediment.

### Lithic raw materials used

The lithic raw material analysis is ongoing and here we report identification of silicites and other rocks from 1369 artifacts from the whole sequence which represent more than half (57%) of the entire collection of individually provenienced lithics. In total, we identified eighteen groups of silicites and ten other rocks thus far **(Tables 3, 4)**, with flint being the most abundant rock. The characteristics of each silicite group are described in **S2 Table** while the variety of microfacies for some of the groups is depicted in the **S1 to S6 Figs**.

**Table 3:** Distribution of raw material types, geological provenance and geotopes of the flint used in IUP lithic assemblages from Bacho Kiro Cave, excavations 2015–2021.

	Raw material types	Primary stratigraphic origin	Geotope	Nb artefacts IUP Layers	
**Silicites**	**Group 11**	Lower Cretaceous (Aptian)	Ludogorie	587	833
	**Group 12**	Lower Cretaceous (Aptian)	Ludogorie	1	
	**Group 13**	Lower Cretaceous (Aptian)	Ludogorie	203	
	**Group 16**	Lower Cretaceous (Aptian)	Ludogorie	3	
	**Unprecise Lower Cretaceous**	Lower Cretaceous (Aptian)	Ludogorie	39	
	**Group 22**	Lower Cretaceous (Aptian)	Ravno-Kamenovo area	3	4
	**Group 23**	Lower Cretaceous (Aptian)	Ravno-Kamenovo area	0	
	**Group 24**	Lower Cretaceous (Aptian)	Ravno-Kamenovo area	1	
	**Group 33**	Upper Cretaceous (Campanian)	Asenovo—Nikopol area	214	227
	**Group 34**	Upper Cretaceous (Maastrichtian)	Ohoden area	4	
	**Group 35**	Upper Cretaceous (Campanian)	Muselievo area	9	
	**Group 37**	Upper Cretaceous (Campanian)	Shumen area	0	8
	**Group 61**	Upper Cretaceous (Campanian)	Shumen area	8	
	**Group 21**	Upper Cretaceous (Campanian?)	Unknown (Danubian valley?)	0	4
	**Group 36**	Upper Cretaceous	Unknown (Danubian valley?)	4	
	**Group 42**	Upper Cretaceous	Unknown (Danubian valley?)	0	
	**Group 41**	Eocène	unknown	0	74
	**Group 62**	Upper Cretaceous	unknown	1	
	**Unprecise Upper Cretaceous**	Upper Cretaceous (Campanian)	Pleven or Shumen area	73	
	**Group 51**	Eo-Oligocen	Rhodopes mountains	0	0
	**Indeterminate**	-	-	9	9
**Volcanogenic rocks**	**Rhyolithe porphyrique**	local < 10 km	local < 10 km	23	37
	**Metarhyolith**	local < 10 km	local < 10 km	1	
	**Rhyolithe**	local < 10 km	local < 10 km	4	
	**Basalt**	local < 10 km	local < 10 km	3	
	**Quartz**	local < 10 km	local < 10 km	6	
**Sandstone**	**Carbonated sandstone**	local < 10 km	local < 10 km	8	28
	**Quartzitique sandstone**	local < 10 km	local < 10 km	14	
	**Iron sandstone**	local < 10 km	local < 10 km	1	
	**Psammite**	local < 10 km	local < 10 km	3	
	**undifferenciated Sandstone**	local < 10 km	local < 10 km	0	
				Total	1222

**Table 4:** Raw material types per sector and excavated layers in Bacho Kiro Cave, excavation 2015–2021.

**Layers**	**A0**	**A1**	**A2**	**B**	**B/C**	**C**	**G2**	**G3**	**H/I**	**I**	**I/J**	**J**	**N1-2**	**N1-3c**	**N1-H**	**N1-H/I**	**N1-I**	**N1-I/J**	**N1-J**	**N1-J/K**	**N1-K**	**Total**	**%**
**Raw material types**																							
**11**	1	1	1	2	1	2	1	1			1	2		1	18	49	482	33	2			598	43.49
**12**																	1					1	0.07
**13**			1	4						1	1				6	14	171	7	3			208	15.12
**16**																	3					3	0.22
**21**				1																		1	0.07
**22**		1	1	2		2							1			3						10	0.73
**23**				1		1																2	0.14
**24**																	1					1	0.07
**33**		3	2	27		3		1	1	1	1				5	25	168	12	1		1	251	18.25
**34**						1											3		1			5	0.36
**35**				7	1										1	2	6					17	1.23
**36**															1		3					4	0.29
**37**				2																		2	0.14
**41**				1																		1	0.07
**42**					1																	1	0.07
**51**						1																1	0.07
**61**		1	4	26	2	2											8					43	3.13
**62**																	1					1	0.07
**Aptian (Lower Cretaceous)**						2									1	2	36					41	2.98
**Upper Cretaceous**			3	8		2									5	9	54	4	1			86	6.25
**Rhyolithe porphyrique**																2	19	1	1	1	5	29	2.11
**Rhyolithe**																	4					4	0.29
**Quartzitique sandstone**															1	2	11					14	1.09
**Basalt**					1												2		1		1	5	0.36
**Quartz**																	2		4			6	0.44
**Carbonated sandstone**									1						1	1	4	1				8	0.58
**Iron oxyde**																	3					3	0.22
**Iron sandstone**																	1					1	0.07
**Limestone**																	1		1			2	0.14
**Metarhyolith**																	1				1	2	0.14
**Psammite**																	3					3	0.22
**Calcite**																	1					1	0.07
**Sandstone**	1																			1		2	0.14
**Indeterminate**				7	1	1											8	1				18	1.31
**Total**	2	6	12	88	7	17	1	2	2	2	3	2	1	1	39	109	997	59	15	2	8	1375	100
%	0.15	0.44	0.87	6.4	0.51	1.24	0.07	0.15	0.15	0.15	0.22	0.15	0.07	0.07	2.84	7.93	72.5	4.29	1.09	0.15	0.58	100	

The primary stratigraphic origin, and geotop [58] of each group is given in **Table 3**. The main recognized raw material types used by the IUP humans are allochthonous Lower Cretaceous (Aptian) and Upper Cretaceous (Campanian) flint.

**Fig 3:**
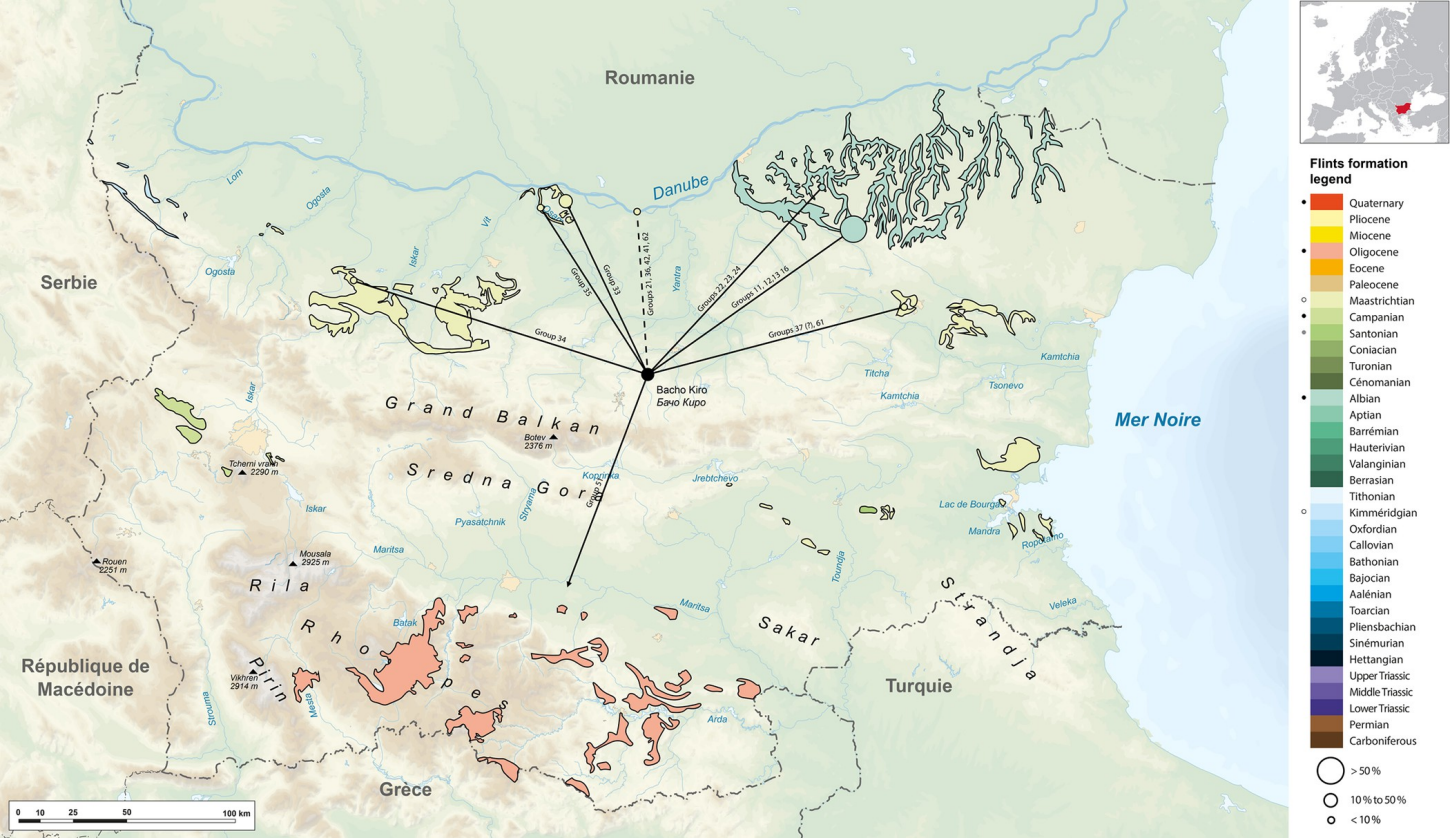
Map of Bulgaria with the location of Bacho Kiro Cave and raw material sources of the silicites found in the IUP layers. Size of the circle correspond to the proportion found in the IUP layers from BK; In the legend dots correspond to the known formation with silicites (white dots: sources not exploited; black dots: sources exploited) author V. Delvigne.

The various groups of Lower Cretaceous (Aptian) flints originate from several sources in northeast Bulgaria in the Ludogorie area (groups 11, 12, 13, 16) and in the Ravno region (group 22, 23, 24) **(Fig 3)**. The geological variation of exogenous silicites and the other rocks, along with their potential distance from their original sources, is detailed in **S3 Table**.

These were mainly collected in sub-primary formations of alterites and colluviums, but some archeological objects testify selection of these flints in alluvial formations. The Aptian flint from the Ludogorie area, distant from 160 to 190 km north-east (groups 11, 12, 13, 16 and unprecise lower Cretaceous) of Bacho Kiro Cave, is represented by 851 artifacts (62,1% of all identified IUP lithics), while groups 22, 23 and 24 from the Ravno region can be detected in only 13 lithic (11%). For 39 (32%) artifacts made out of Aptian flint, the group could not be precisely determined. The Aptian flint is distributed in the majority of the techno-typological artifact categories: unretouched (including cortical) and retouched flakes and blades, other retouched tools, the core, only one core was found in total. The relatively high quantity of found debris suggests that this imported flint was also reduced in the cave **(Table 5)**. Precise counts, taphonomic and techno-economic analysis will be reported after the complete raw material analysis.

**Table 5:** Raw material types per techno-typological categories in the IUP layers (H, H/I, I, I/J, and J) from both sectors in Bacho Kiro Cave, excavation 2015–2021.

Blank type	Pebble	Core	Flake	Laminar flake	Blade	Bladelet	Flake tool	Blade tool	Retouched piece	Burin spall	Scaled piece	Slab	Debris	Bead	Pre-form bead?	Fire flake	Lithoclase	Unknown	Total	%
Raw material																				
**Group 11**		1	300	37	35	33	20	14	7	10	1		126			3			587	48.04
**Group 12**			1																1	0.08
**Group 13**			81	27	17	17	8	3	1	2			46			1			203	16.61
**Group 16**			2							1									3	0.25
**Group 22**			2				1												3	0.25
**Group 24**			1																1	0.08
**Group 33**		1	90	15	6	14	15	7	5	8			51				1	1	214	17.51
**Group 34**			2			2													4	0.33
**Group 35**			5								1		3						9	0.74
**Group 36**			2	1		1													4	0.33
**Group 61**			2	2		1	1		1	1									8	0.65
**Group 62**								1											1	0.08
**Aptian (Lower Cretaceous)**			12	3	3	1	1	3	1				13					2	39	3.19
**Upper Cretaceous**			25	5	5	3	2	5	3	2			22					1	73	5.97
**Rhyolithe porphyrique**	2		13										8						23	1.88
**Rhyolithe**			2										2						4	0.33
**Quartzitique sandstone**			4									1	4					5	14	1.14
**Basalt**	1		2																3	0.25
**Quartz**	2		1										3						6	0.49
**Carbonated sandstone**	1		1										3		1			2	8	0.65
**Iron sandstone**																		1	1	0.08
**Metarhyo-lith**							1												1	0.08
**Psammite**	1													1	1				3	0.25
**Indeter-minate**			2			1			1				5						9	0.74
**Total**	7	2	550	90	66	73	49	33	19	24	2	1	286	1	2	4	1	12	1222	100
**%**	0.57	0.16	45	7.37	5.41	5.98	4	2.7	1.56	1.97	0.16	0.08	23.4	0.08	0.16	0.33	0.08	0.99	100	

The second most abundant group of flints is rich in planktonic microfossils (group 33) and is attributable to Upper Cretaceous (Campanian) formations. This group is represented by 251 artifacts or 18.3% of all identified flints in the IUP layers. The flint outcrop is located next to the Danube River between Nikopol and Asenovo, 110 to 130 km northwest from the cave **(Fig 3)**. To this group we can add five artefacts coming from the west (Ohoden region, group 34) and 17 coming from the Muselievo area, along the Danube Valley, due to the abundance of bryozoans and algae (group 35).

In total, 273 artifacts representing 19.9% are made out of Upper Cretaceous flint from the middle north part of Bulgaria. The distribution of this flint follows a similar pattern in the techno-typological categories like the Lower Cretaceous flint **(Table 4)**, attesting that similar kinds of products were manufactured from allochthonous Lower and Upper Cretaceous flint originating from two distinct areas.

Related to these areas we have to add three groups (21, 36 and 42) whose precise origin remains unknown, but whose composition unquestionably evokes the Upper Cretaceous (probably the Campanian or the Santonian). However, no sample observed in the lithoteque of the Man and Earth Museum in Sofia presents such facies. Possible origin for these six artifacts (0.4%) are the left bank (or the left terraces) of the Danube.

Cretaceous terrains that provided Campanian flints also have outcrops in the vicinity of Shumen, more than a hundred kilometers west of the site. They differ in particular from the Campanian of the Asenovo-Nikopol region (see above) with their increased amounts of planktonic microfauna, a decrease in the remains of spicules and a generally black matrix. 45 objects (3.2%) represent this geotope and bear cortical surfaces attesting to the selection of raw material in alterite formations (group 61) or in terraces (group 37). However, group(s) of 86 artifacts (6.3%) made in Upper Cretaceous flint are not identifiable due to their excessive white patina and thus cannot be attributed to a particular geotope.

Finally, note the existence within the archeological collection of a single artifact made in hydrothermal silica (group 51), which remind us of geological formations that outcrop further south in the Tertiary deposits of the Rhodopes. A systematic survey of such deposits in this area would make it possible to locate the source of these artifacts.

**Fig 4:**
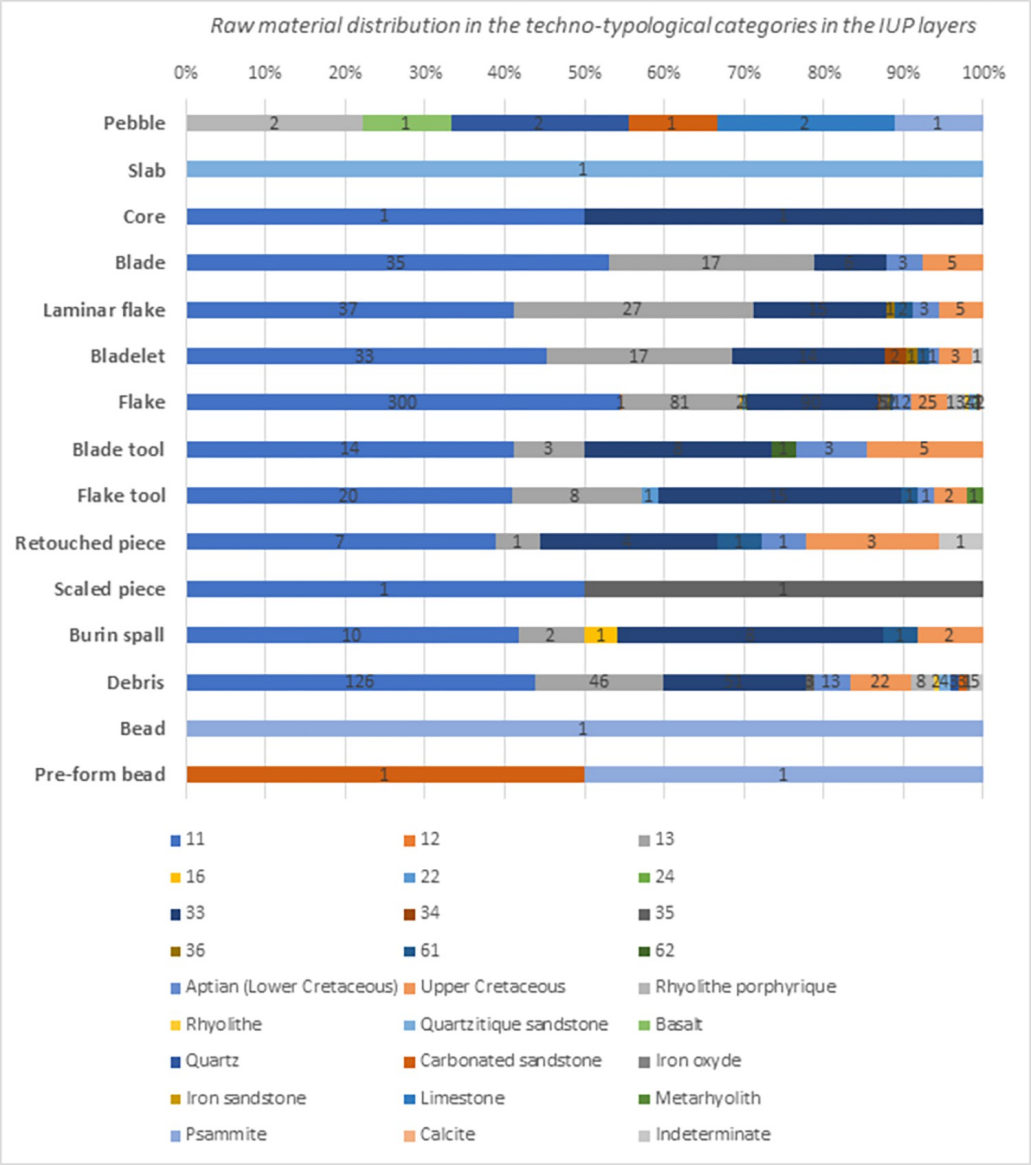
Raw material types distribution in the techno-typological groups in the IUP layers in Bacho Kiro Cave, excavation 2015–2021.

Metamorphic and sedimentary rocks (e.g., rhyolite porphyric, rhyolite, quartzitic sandstone basalt, quartz, carbonated sandstone), likely of local origins, are represented in the techno-typological categories by flakes, pebbles and debris, suggesting that they were used rather sporadically and reduced in place **(Table 4, Fig 4)**. Sedimentary sandstone, such a psammite, is used in one case for bead manufacture of similar morphology as the pierced ivory beads [59]. In two other cases, it is very likely that psammite is in a stage of pre-shaping **(Fig 4)**. A small psammite unworked pebble suggests stone beads were likely manufactured in place.

### Techno-typological classification of the lithic assemblages

The IUP lithic assemblages consist of 2247 analyzed artefacts, sorted by layer in **Table 6**.

**Table 6:** Count of techno-typological categories in the IUP layers H, H/I, I, I/J and J from the Main Sector and Niche 1 in Bacho Kiro Cave.

**Techno-typological categories**	**H**	**%**	**H/I**	**%**	**I**	**%**	**I/J**	**%**	**J**	**%**	**Total**	**%**
**Cores (including bipolars)**	2	4.08	1	0.37	24	1.37	3	2.36	2	4	32	1.42
**Crested blades**		0	1	0.37	12	0.69		0		0	13	0.58
**Tablets**	1	2.04	1	0.37	10	0.57		0		0	12	0.53
**Flakes and flake fragments**	22	44.9	97	35.93	634	36.21	37	29.14	16	32	806	35.87
**Blades and blade fragments**	6	12.24	34	12.59	275	15.71	21	16.54	5	10	341	15.18
**Retouched tools and tool fragments**	13	26.54	54	20	240	13.71	23	18.11	8	16	338	15.04
**Retouch waste (flakes and chips)**	1	2.04	16	5.93	196	11.19	16	12.6	6	12	235	10.46
**Debris**	3	6.12	35	12.96	150	8.57	13	10.24	4	8	205	9.13
**Split pebbles**		0	1	0.37	5	0.28	2	1.57	1	2	9	0.4
**Bipolars blanks**		0	5	1.85	36	2.06	2	1.57		0	43	1.91
**Scaled pieces**		0	14	5.19	92	5.25	4	3.15	1	2	111	4.94
**Bipolar fragments**		0	2	0.74	19	1.08		0		0	21	0.94
**Bipolar debris**		0		0	9	0.51	2	1.57	1	2	12	0.53
**Anvils (sandstone slabs)**		0	7	2.59	20	1.14	1	0.79	1	2	29	1.29
**Manuport (including worked ones)**	1	2.04	2	0.74	29	1.66	3	2.36	5	10	40	1.78
**Total**	49	100	270	100	1751	100	127	100	50	100	2247	100

The representation of the major lithic classes is as follows: flakes 35.87%, blades 15.18%, retouched blanks 15.04%, and cores 1.42%. The low presence of cores and characteristic products of core shaping such a crested blade (0.58%) and core tablets (0.53%) suggest that not much flaking was done in place. In contrast, relatively high presence of retouch flakes and chips from resharpening of the tools (10.46%) indicates blanks were retouched on place. The presence of all kinds of pieces with bipolar percussion stigmata (pebbles, cores, blanks, fragments and debris), suggests that blades and flakes were systematically used as cores on anvil for production of small blanks **(Table 6)**. The group of bipolar artifacts together consists of 219 pieces or 9.75% of the whole lithic collection. The techno-typological classification of bipolar artifacts, along with the presence of artifacts representing all phases of the bipolar reduction sequences, attests to the systematic application of the on-anvil technique on-site **(Table 7)**. A large number of sandstone slabs with impact marks indicates that they were used as anvils (n = 29) and this technique was a commonly practice at the site.

**Table 7:** Count of bipolar techno-typological categories in the IUP layers from the Main Sector and Niche 1 in Bacho Kiro Cave.

**Bipolar techno-typological categories**	**H**	**H/I**	**I**	**I/J**	**J**	**Total**	**%**
**Bipolar cores**	2	1	16	3	1	23	10.5
**Bipolar fragments**		2	19			21	9.6
**Bipolar blanks**		5	36	2		43	19.6
**Scaled pieces**		14	92	4	1	111	50.7
**Bipolar debris**			9	2	1	12	5.5
**Bipolar pebbles**		1	5	2	1	9	4.1
**Total**	2	23	177	13	4	219	100

### Cores

**Fig 5:**
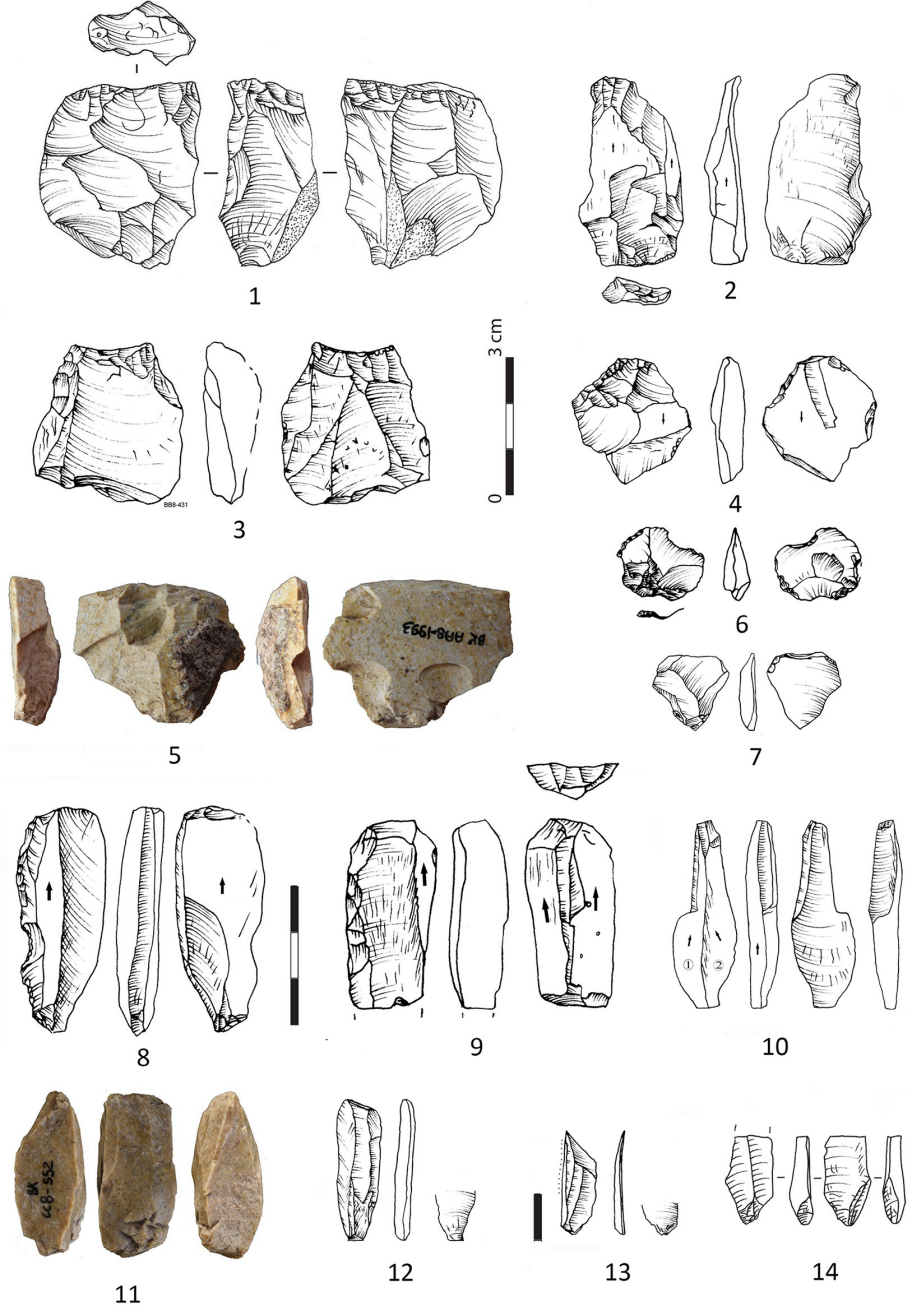
Cores and products in the IUP layers, from Bacho Kiro Cave, excavation 2015–2021. Bipolar cores (1, 3, 5, 8–9, 11) and bipolar products (2, 4, 6, 7, 10, 12–14). Note Nr 9 is a bipolar core on previous endscraper on blade. Note for Nr 18 and 19 an arrow indicates the debitage direction of the previous surfaces while the last removals (*redébitage* or reflaking, reshaping are drawn with ripples), (Pictures T. Tsanova, drawings T. Tsanova and I. Krumov).

The cores represent quite a low percentage of the IUP lithic assemblage 1.42% (n = 32) and there are disconnected with the blade technology presented in the IUP layers. Cores are subdivided according to their knapping technique: the bipolar on anvil cores are 3 times more frequent (75%) than the cores reduced by freehand direct percussion technique (25%) (**S4 Table**). For some of the cores (n = 3) stigmata of both techniques are readable on the same core [60] which means that both techniques were interchangeable at the same core in order to extract more flakes. Almost all cores (except 2) are in the final reduction stage with lengths between 45 and 20 mm and widths between less than 40 mm to 10 mm, while the thickness varies from 25 to 11 mm. One-fourth of the core blanks are unidentifiable (25%) because of the reduced core sizes, nevertheless, a clear tendency is to transform the blanks into bipolar cores. The most frequent blanks for bipolar cores are flakes (25%), and blades or flakes (25%) are impossible to determine because of the recovered debitage scars on the original blank arises **(S4 Table)**. Flakes were turned into regular freehand cores in 3 cases **(S4 Table)**, one freehand core is on concretion and is the largest and less reduced core in the group, over 70 mm **(S7 Fig)**. Freehand cores are turned into bipolar cores in 2 cases **(Fig 5: 1, 11)**. The core general morphology is prismatic and flatted lenticular, both types are equally represented and consist of more than half of the cores. More than 20% of cores are discarded shapeless while 9.3% remain in pyramidal form, and the other 9.3% are unclassifiable. For almost half of the cores the flaking surface is oriented on the wide side of the core blank (n = 15), while in 4 cases the flaking surface is implanted on the narrow side (the edge), and in 1 case is combined: on the narrow and the wide side **(Fig 5: 1)**.

**Fig 6:**
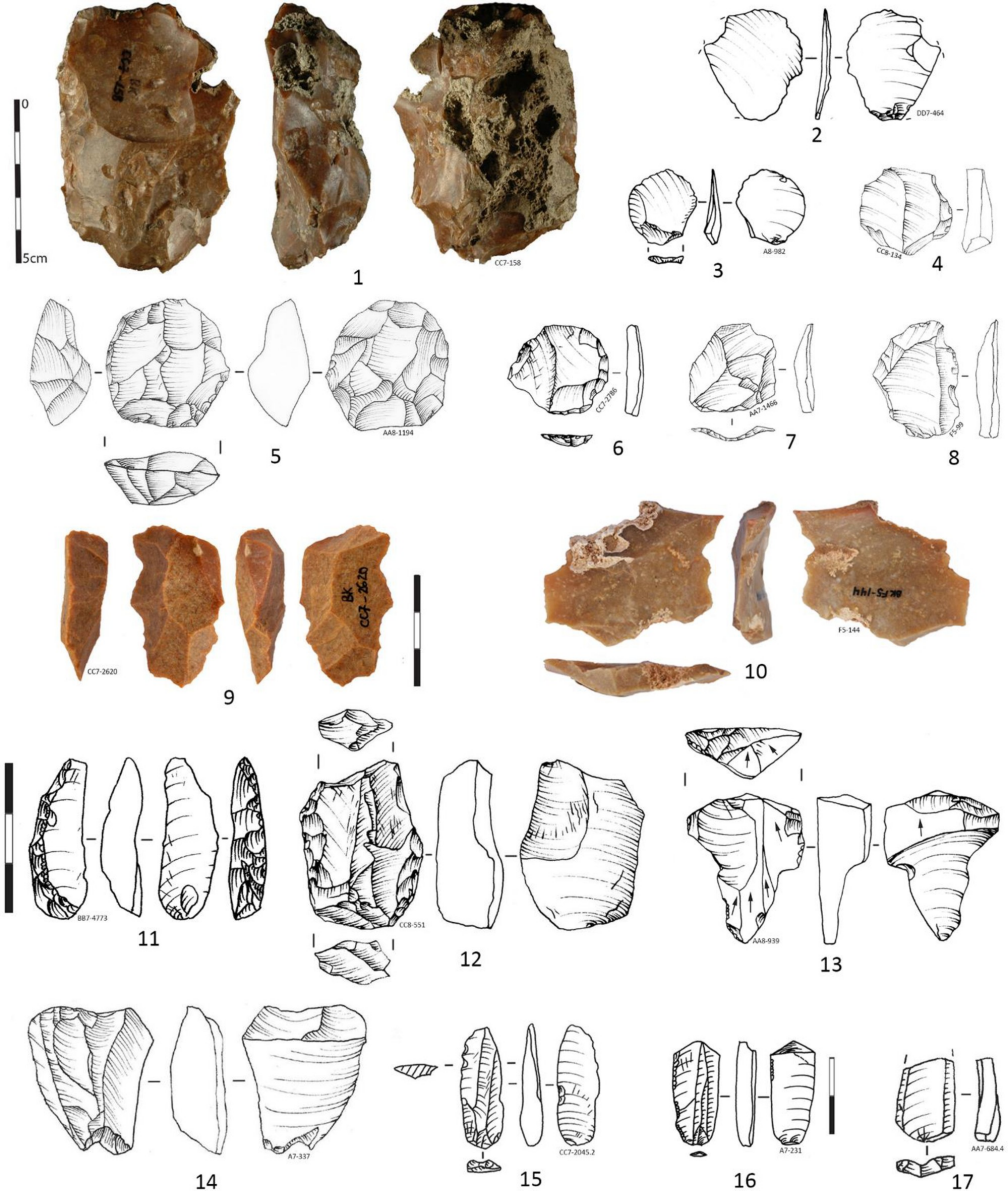
Cores and products in the IUP layers, from Bacho Kiro Cave, excavation 2015–2021. **1** Core on cortical flake Kombewa type; **2–3** Biconvex small flake Kombewa type; **4, 6–8, 10** Flakes of Levallois type; **5, 9** Cores of Levallois concept; **11** Neo-crest corresponding of retouched edge of a tool (typologically is a burin spall); **12** Reflaking the lower face of a retouched tool (typologically corresponding to flat burin); **13** Overshot flake with negatives of reflaking (the blank was likely a heavy blade) **14** Bipolar core for bladelets; **15–17** Bladelets whose nr 15 resulting of bipolar percussion (pictures Ts. Tsanova, drawings 1–4, 6–8, 11–12 I. Krumov, 5 S. Sirakova).

Reduction schema is most frequently unidirectional for the 2 general groups of cores (34.4%) but for the bipolar cores unidirectional and bidirectional reduction is equally presented while more numerous are the bipolar cores with unidentifiable reduction schema. With centripetal negatives of reduction are 4 cores (12.5%). The presence of cortex on the cores is low. Only one bipolar core on quartz pebble is with a totally cortical back, while 4 others are with cortical remains (< 50% cortex), and 3 are partially cortical (> 50% cortex). In total 9 cores are with volume shaping out removals on both lateral edges and on the tip. All 5 Levallois cores exhibit negatives of shaping out, as well as 1 other freehand core, and 3 bipolar cores. The bipolar cores are systematically reduced without any preparation or shaping out (79.2% of the bipolar cores), in this way, they are more opportunistic and the lateral edges and arises of the core blank (previous retouched tool, or blade fragment) have role of a crest **(Fig 6: 11, 12)**.

Cores of Levallois concept with centripetal scar pattern (n = 4) are almost all with facetted platforms including a Levallois core fragment **(Fig 6: 1, 5, 9)**. All identified cores of Levallois concept (n = 5, 15.6%), are freehand cores **(Fig 6: 1, 5, 9)**, except one which is mixed: started as freehand and finish as bipolar on anvil **(Fig 5: 5)**. Two core results from Kombewa method **(Fig 6: 1, 12)** already described from previous collection study [38]. Both Levallois and Kombewa methods are used on place for the production of small circular Levallois **(Fig 6: 4, 6–8)** and biconvex (Kombewa) flakes **(Fig 6: 2, 3)**. Almost all freehand cores display negatives from last flakes removals (n = 7) while the bipolar cores show flakes (n = 12), bladelet removals (n = 8), and bladelets and small flakes (n = 3). Almost 60% of all cores are abandoned at final stage of flake production while the 25% of the bipolar cores have produced bladelets and small flakes with bladelets (9.37%).

Core groups shows that they are unrelated to blade production however they are manufactured on the same flint types like the rest of the assemblages, which make them integral part of the IUP assemblages and unrelated to the underlaying Middle Paleolithic. Moreover, fragmented blades and flakes were systematically used as core blanks, *redébitage*, also reused in tools. **S7 Fig** shows the sizes of the 2 core groups overlap. However freehand cores are shorter and larger (more reduced in length and less in width) while bipolar cores are longer and narrower **(S7 Fig left)**. In thickness, the freehand cores also appear more reduced than the bipolar cores **(S7 Fig right)**. In summary, all cores result from the on-site intense reduction of blanks and tools, primarily through secondary knapping, mostly using the bipolar technique. This secondary knapping transforms some of the blanks and tools into more or less typical scaled pieces.

### Laminar technology

The imported blades resulting from distinct *chaines operatoires* are technologicaly and morphologically coherent originating from a method and technique with similar finality: straight heavy blades with lengths up to 120 mm, width up to 4 cm, and with the average thickness of 6.92 mm (measured on the complete blades) **(Table 8)**. The absence of corresponding blade cores, cortical and characteristic products of reshaping laminar cores are indicative for off-site laminar productions and import of finished products. Additionally, the low ratio core to tool (0.02) supports this previously considered interpretation [14, 38]. Based on consistency in attributes and shapes, those reduction methods and techniques seem to had been somewhat systematic and oriented towards the production of elongated products (with 1/3 ratio between length and width) with straight profiles, and triangular and trapezoidal sections.

**Table 8:** Average dimensions of the blades in the IUP layers from Bacho Kiro Cave.

Blades mean values	Length (mm)	Width (mm)	Thickness (mm)
**Complete blade tools**	46.33	18.04	6.92
**Complete and fragmented blade tools**		20.27	6.56
**Non-retouched blades**	32.77	13.07	5.03
**Complete and fragmented non-retouched blades**		16.3	5.22

In the absence of corresponding blade cores in the IUP layers from Bacho Kiro Cave, we turn to the volumetric cores from Temnata Cave Layer 4 to discern the methods of laminar production. The comparison of the IUP blade productions from both Bacho Kiro and Temnata Caves (Layer 4) is revealing, suggesting that laminar products from both sites are comparable in their morphologically straight blades with parallel edges and in their continuous production of large series of morphologically varied blades, ranging in their dimensions from large to small, along with the manufacture of small blades on flake lateral edges (burin core-tools) [12, 38]. Despite differences in both IUP assemblages, stemming from locally available raw materials in Temnata Cave and imported laminar products from a long distance in Bacho Kiro, we assume that the debitage method is likewise: utilizing unidirectional and bidirectional reduction methods on volumetric, prismatic, and sub-prismatic core volumes, as well on the narrow and wide core’s sides, employing a direct percussion with a hard hammer. Another argument possibly linking the laminar debitage concept of Temnata Layer 4 with the IUP’s missing blade cores record is the distribution of facetted and dihedral platforms in both assemblages. If the large imported blades in the IUP assemblages from Bacho Kiro Cave were produced by Levallois method (claimed by others below), we would expect a higher frequency of facetted and dihedral platforms. Instead, these platform types are more frequent in the assemblages from Temnata Cave, Layer 4 where Levallois method is absent [12]. In the IUP from Bacho Kiro Cave, facetted platforms among the blades account for 18.1%, while in Temnata, they are 23.5%. Dihedral blade platforms in Bacho Kiro Cave represent 0.7%, whereas in Temnata, they are 4.7% **(S5 Table)**.

In the IUP layers from Bacho Kiro Cave most of the raw blades exhibit unidirectional parallel scar pattern (84.4%), likewise is the case in Temnata layer 4 (39,6%) [38]. In Bacho Kiro Cave there are 11 retouched blades (6.96%) with unidirectional convergent negatives (**Table 9**) and 13 (8.22%) that resulted from bidirectional blade production with two opposite striking platforms. The blade debitage schema’s are comparable to the layer 4 from Temnata where the blades with bidirectional removals are 9.6% and those with convergent unidirectional removals are 5.4% [38].

**Table 9:** Dorsal scar pattern comparing retouched blade tools with unretouched blades in the IUP layers from Bacho Kiro Cave.

Dorsal scar pattern	Blade	%	Blade tools	%
**Cortical** *	4	1.13		
**Cortical remains** **	13	3.68		
**Unidirectional parallel, partially cortical** **	9	2.54	6	3.8
**Unidirectional parallel, cortical remains** ***	11	3.11	3	1.9
**Unidirectional parallel**	299	84.47	119	75.32
**Unidirectional convergent**	1	0.28	11	6.96
**Unidirectional convergent, cortical remains**			1	0.63
**Bidirectional opposite, cortical remains**	1	0.28		
**Bidirectional opposite**	14	3.95	13	8.22
**Bidirectional convergent**	1	0.28		
**Multidirectional**	1	0.28	2	1.27
**Undeternimable**		0	3	1.9
**Total**	354	100	158	100

*100% cortex

**partially cortical <50%

***cortical remains >50%

**Fig 7:**
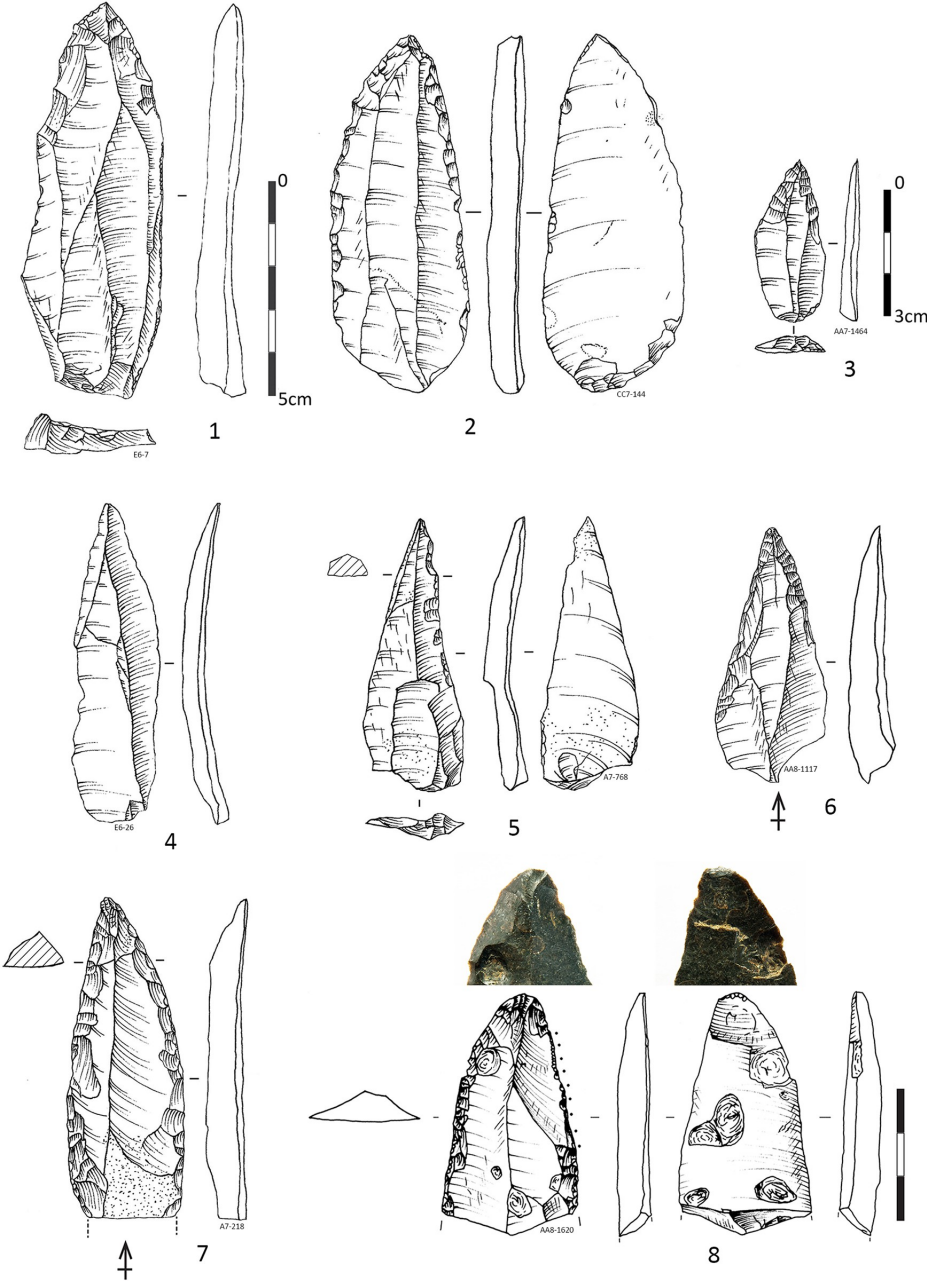
Pointed blades from the IUP layers in Bacho Kiro Cave. **1–2** Heavy gauge blades retouched in the distal part with number 2 could be classified as side-scraper, the proximal end is shaped out by inverse retouch; **3** Small pointed blade. Note that numbers 2 and 3 are of similar morphology slightly truncated on the left distal edge while the right edge is of straight delineation; **4–5** Pointed unretouched and slightly retouched blades with shape predetermined in the debitage method, number 5 is only slightly retouched on the right edge forming a notch below the apex; **6** Pointed elongated triangular flake; **7** Pointed blade from bidirectional reduction and with missing proximal part; **8** Long distal fragment of pointed retouched blade with a step terminating a bending fracture on the apical ventral surface (negative of the fracture precedes the burnt alteration), (Drawings I. Krumov and T. Tsanova).

In the IUP layers of Bacho Kiro cave, there is also evidence of heavy blades detached from core with opposite and shifted adjacent flaking platforms **(Fig 7: 1)**. This clue suggests that a portion of the imported blades originated from volumetric bidirectional debitage with shifted adjacent flaking platforms, similar to the laminar concept observed in Temnata Layer 4 [38]. This method of blade production involves systematically flaking from adjacent platforms located on both the narrow and wide portions of the core. It allows for the production of blades with convergent or parallel edges, as well as intercalated large blades resulting from reshaping the flaking platforms (such as intercalated overshot blades, identical to those found in Layer 4 of Temnata Cave). By alternating striking platforms, manufacturer can control the shape and size of the blades, ensuring consistency in morphology and maximizing the use of the stone raw material.

The degree of fragmentation **(S6 Table)** and reworking of the imported blades of Layer I and J limit the precise reconstruction of the original blades manufacturing methods. Nevertheless, there are indications that at least one part of imported blades originated from volumetric unidirectional prismatic **(Figs 7: 2; 8: 3–5)**, bidirectional **(Figs 7: 7, 8; 8: 2, 8)** and also bidirectional pyramidal cores **(Figs 7: 1, 4, 8: 7)**.

**Fig 8:**
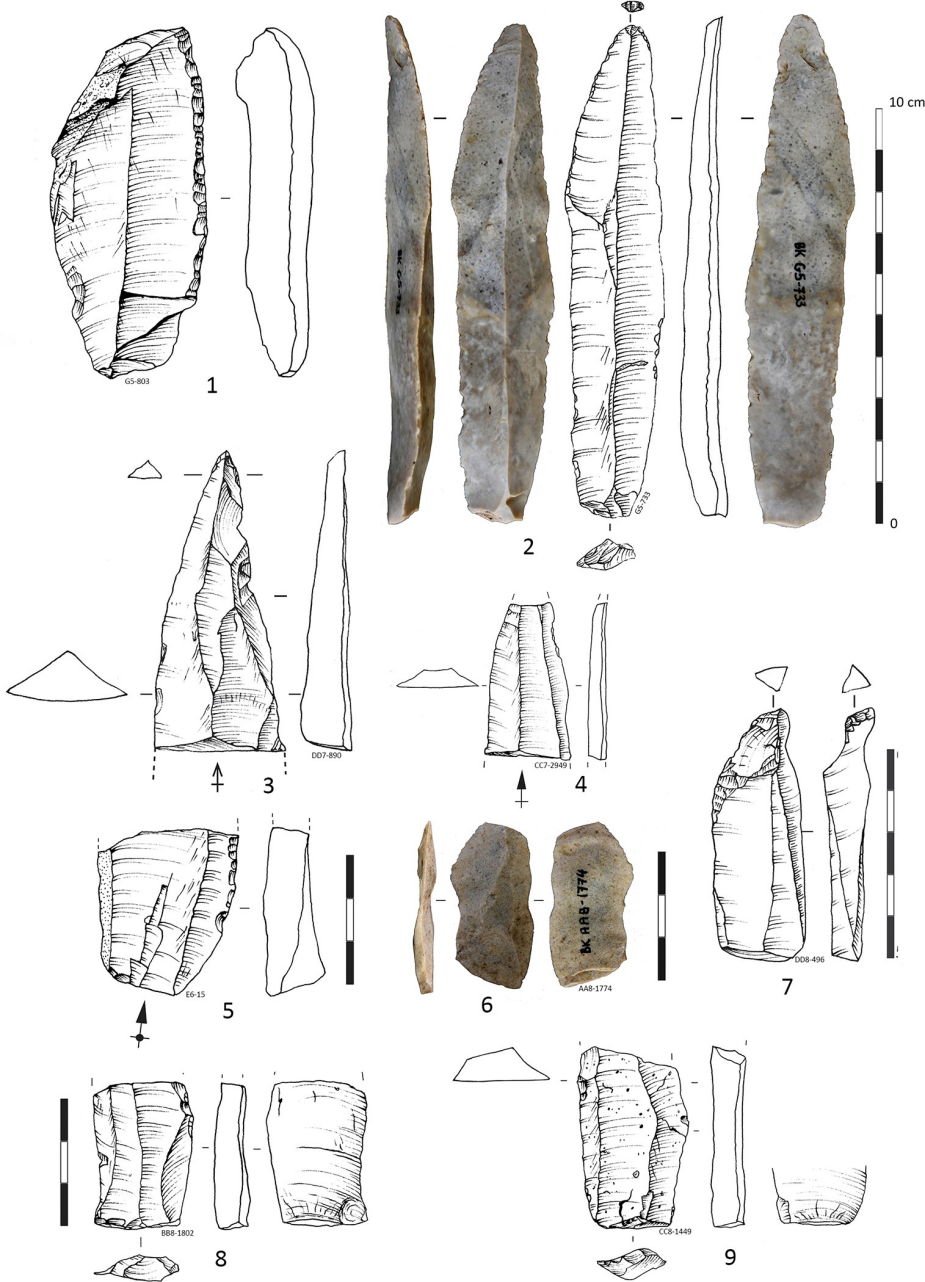
Laminar products, IUP layers, Bacho Kiro Cave. **1** Laminar retouched flake with distal cortex; **2** Blade with convergent edges from bidirectional debitage; **3** Pointed blade (predetermined shape) long distal-medial fragment; **4** Blade long medial fragment; **5, 8, 9** Blade proximal long fragments (whose nr 5 with lateral cortex and nr 8 with bidirectional negatives); **6** Blade fragment with convergent scar pattern, **7** Overshot blade with retouch and bidirectional scar pattern, resulting from volumetric debitage (Pictures Ts. Tsanova, drawings I. Krumov).

If we look at the cores produced on-site, we see that the humans employed reduction on both the narrow and wide sides of the core for production of small blades **(Fig 5: 1)**, but also, they produced small rectangular blades from previous blade tools **(Fig 5: 8–14)**. The manufacturers also employed the Levallois method for production of small flakes **(Fig 6: 1, 4–10)** which is an element similar to Bohunician where at the final stage of blade reduction the cores are transformed into Levallois cores [61]. The most common platform type for blades and flakes are unprepared plain (over 50% for both groups of blanks), with notable 18.1% facetted platforms for the blades and 12.8% for the flakes **(Table 10)**.

**Table 10:** Types of blades and flakes platforms (counts for unretouched and retouched blades and flakes, complete items and all proximal fragments) in the IUP layers from Bacho Kiro Cave. Percentage of each platform type is calculated from the total value of the blades and flakes categories.

Platform types	Blades	%	Flakes	%	Total	%
**Cortical**	2	0.77	17	2.51	19	2.03
**Plain**	134	51.74	357	52.73	491	52.45
**Dihedral**	2	0.77	14	2.07	16	1.71
**Facetted**	47	18.15	87	12.85	134	14.32
***"Chapeau de gendarme"***		0	10	1.48	10	1.07
**Linear**	27	10.42	126	18.61	153	16.35
**Punchiform**	4	1.54	9	1.32	13	1.39
**Shattered** *	1	0.39	6	0.89	7	0.74
**Smashed** **	10	3.87	6	0.89	16	1.71
**Broken** ***	28	10.81	27	3.99	55	5.88
**Undeterminable**	4	1.54	18	2.66	22	2.35
**Total**	259	100	677	100	936	100

* Bipolar blank often exhibit a distinct shattered platform, featuring bulbar scars on the ventral face and small step scars and splintering on the dorsal face at the impact area [62], or often results in the formation of irregular cracks across the surface of the platform

** a smashed (or crushed) describes a platform that has been forcefully compressed or flattened, also deformed. This compression can occur due to intense shock or percussion, such as from bipolar percussion

*** A broken (missing) platform generally refers to a platform that has suffered damage or detachment, resulting in a break or separation from the blank. This can occur during the knapping process or as a result of post-depositional processes.

### Primary knapping techniques

**Fig 9:**
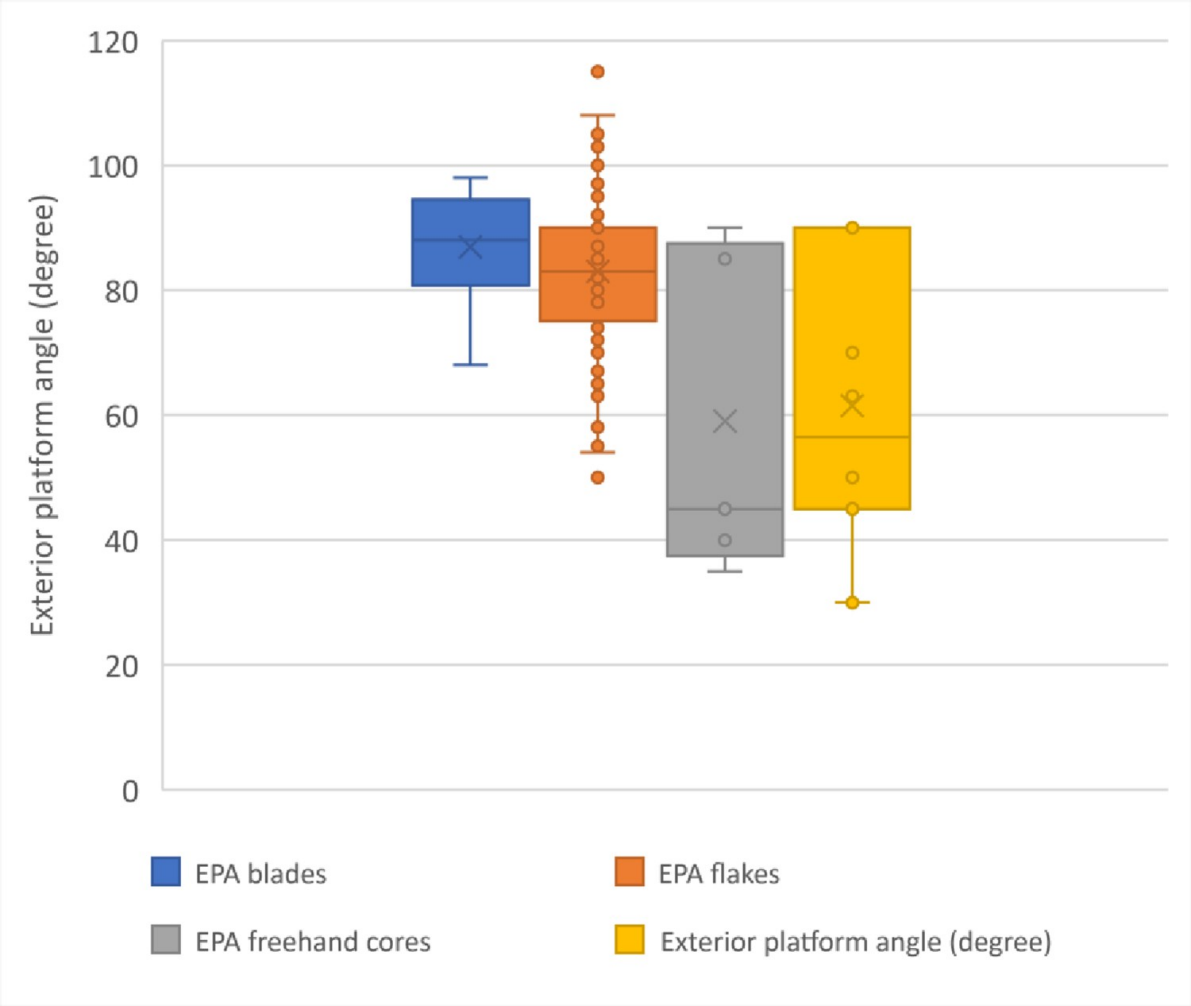
Plot of the exterior platform angles (EPA) of blades, flakes, freehand and bipolar cores, IUP layers, Bacho Kiro Cave.

Exterior platform angle (EPA) of the products, platform size, impact point and bulb characteristics are essential for knapping techniques reconstruction [63]. The results are still preliminary, but describe the general tendency of the lithic assemblages. EPA and platform dimensions are measured for 23 blades and 103 flakes. EPA for blade and flakes follow similar pattern with a mean value for the EPA blade angle of 86.9° and a mean value for the EPA flake angle of 83.0° **(Fig 9)**. **Fig 9** show also the EPA cores and demonstrate that blades and flakes presented at the site are unconnected with the 2 main cores group, with mean EPA value for the freehand cores of 59° and 61.5° for the bipolar cores.

**Fig 10:**
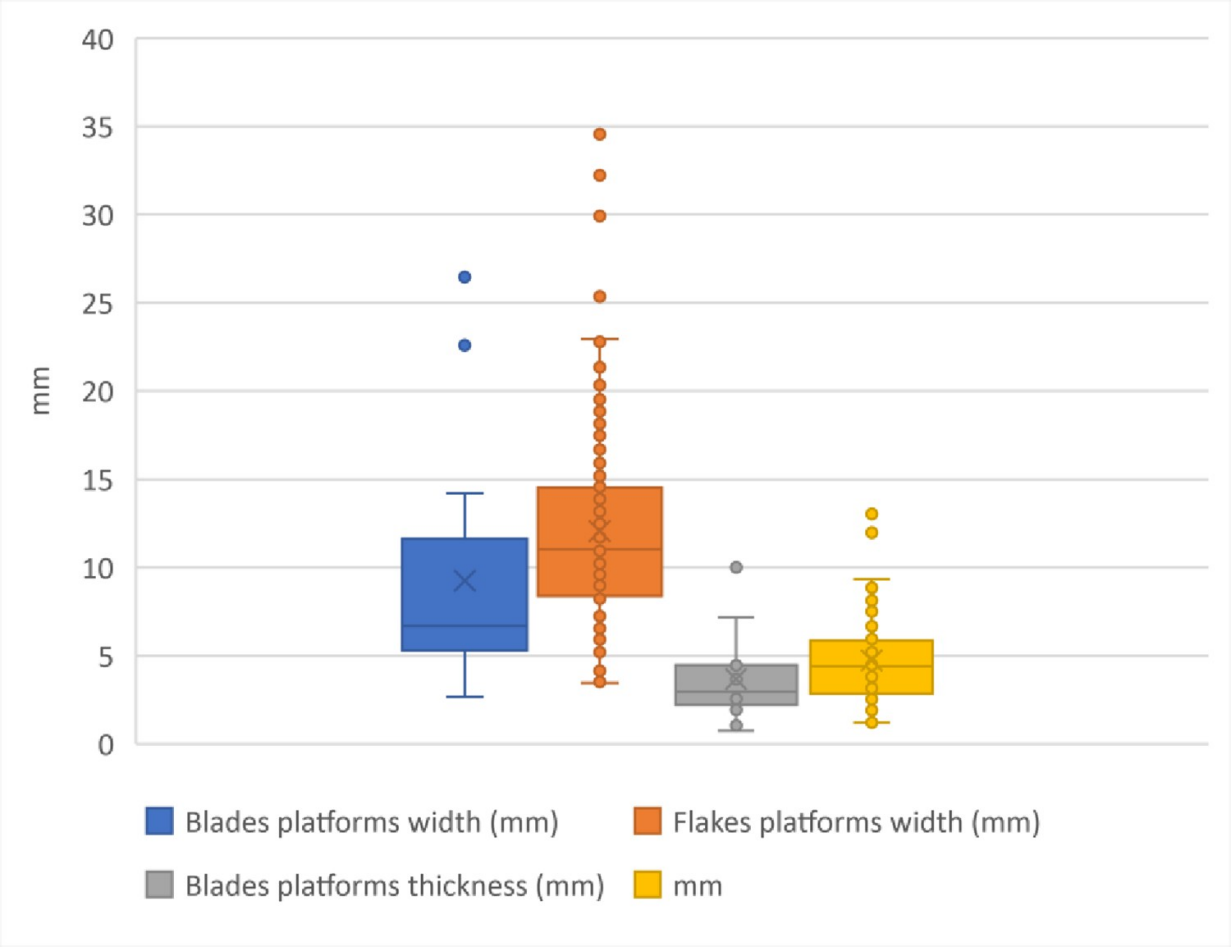
Box & whisker plot of the blades and flakes platforms dimensions (width and thickness), IUP layers, Bacho Kiro Cave.

The open angle of EPA more than 80°, combined with the platform sizes of the blades: majority of blades are with platforms wider between 6 and 12 mm, and thicker between 3 and 5 mm attest the use of hard hammer **(Fig 10)**.

The flakes platforms are clearly larger and thicker than the blades ones. The percussion bulbs are in general well pronounced with clear impact marques (see examples on **Fig 8: 8, 9**) except for the smallest blades where the low thickness of the laminar blanks imposes a more tangential knapping gesture.

### On-site knapping activities: “retouched” product, fragmentation and *redébitage*

**Fig 11:**
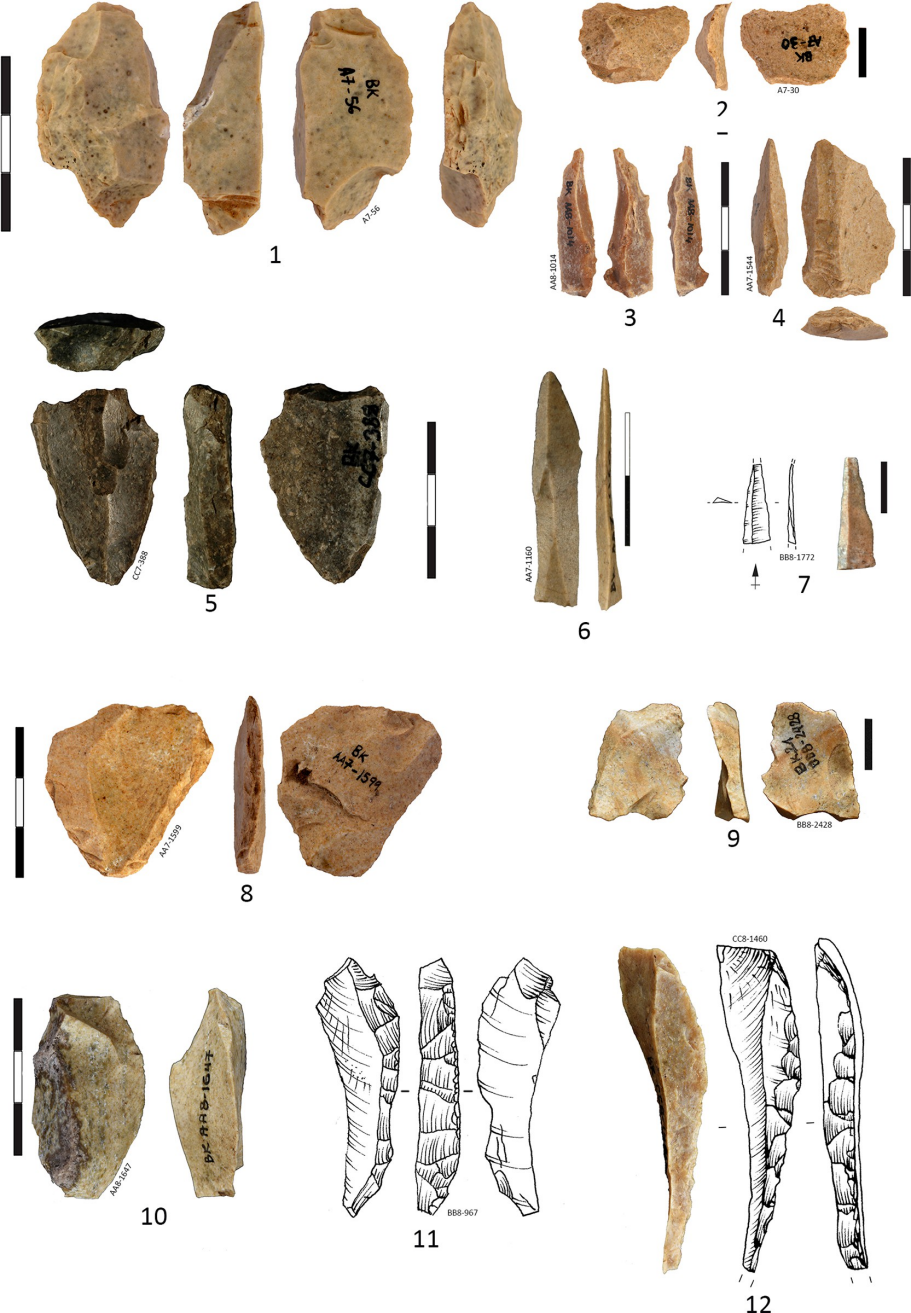
Examples of curation- *redébitage* modalities from the IUP layers in Bacho Kiro Cave. **1** On a edge of a retouched flake (ex-sidescraper?) reworked on burin core on anvil, the right edge is reflaked like burin. Theoretically 2, 3, 4 could be products or byproduct of nr 1; **2** Typological “Retouch” flake; **3** Typological “burin” spall (edge of retouched tool); **4** Splintered flake; **5** On a upper face of a blade fragment, Kostienki type- bladelets are extracted from the central arise; **6, 7** straight triangular bladelets likely produced by Kostienki technique; **8** Bipolar core on retouched flake, with clear negative of removed triangular flake from his upper face; **9** Typological “Retouch” flake; **10** Resharpened flake edge, **11–12** Typological “burin” spalls theoretically corresponding to nr 10 (Pictures Ts. Tsanova, drawings I. Krumov).

**Fig 12:**
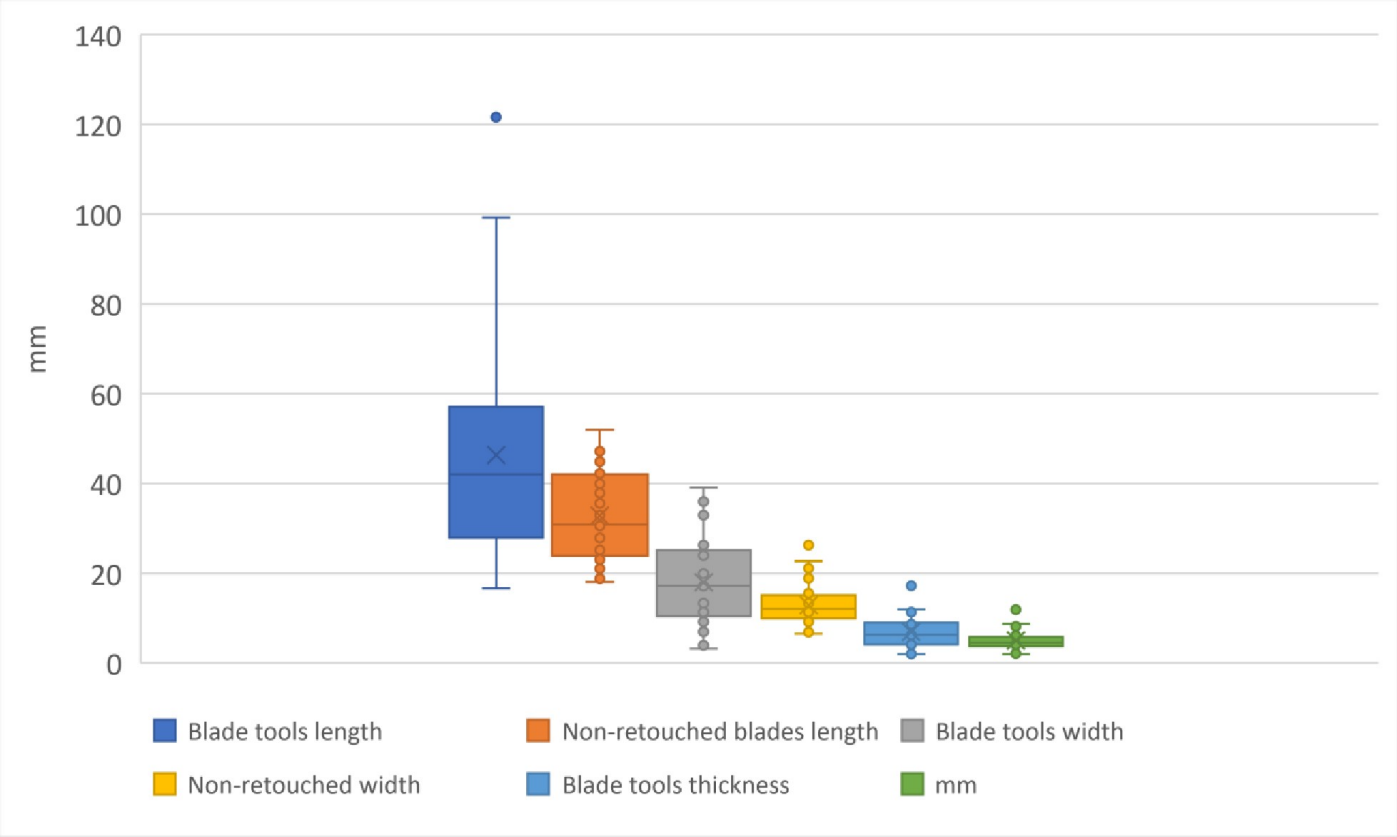
Plot of blades dimensions: Length, width and thickness comparing complete retouched (tools) and complete unretouched blades, IUP layers, Bacho Kiro Cave.

The lithics from the IUP layers were frequently fragmented and reworked for extraction of small blanks of various morphologies, on anvil through bipolar axial knapping or by direct percussion in various techniques: Kombewa, Kostienki like-on a dorsal arises, along-edge fracture of a blank (resulting in a burin) **(Figs 6 and 11)**. Half of the retouched blade tools are longer than 40 mm (with the average length of 46.33 mm), while the unretouched blades are on average 32.77 mm long **(Table 8, Fig 12)**, suggesting that only bigger blanks were in reality retouched, and perhaps small blanks knapped on place were used unretouched.

**Fig 13:**
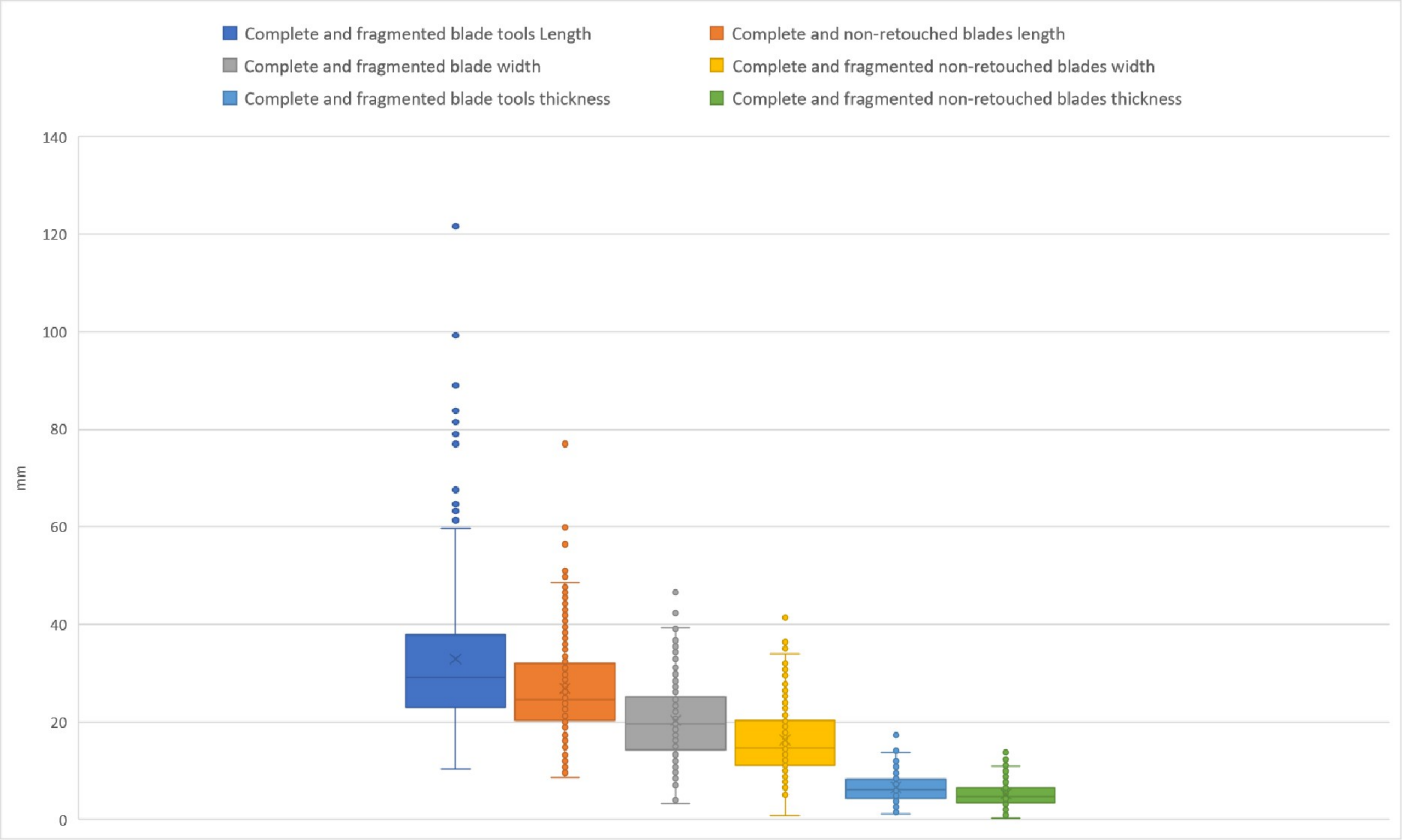
Plot of length, width and thickness of complete and fragmented blades tools and unretouched blades, IUP layers, Bacho Kiro Cave.

Were the non-retouched blanks or the retouched tools more frequently fragmented and further reworked in small cores or new tools? **Fig 12** shows that there is metric continuity between the retouched blade tools and non-retouched blanks, and that blade tools are significantly longer and larger that the non-retouched blades. If we confront the data from complete and fragmented blade tools and non-retouched blades **(Fig 13)** this tendence become even more clear with showing continuity of the record and many outliers of blades tools with length above 60 mm up to 120 mm. This indicate that large blades are fragmented. Half of the blade tools are larger than 20 mm and up to 40 mm indicating that original largest blades are really robust (width is 1/3 of the length).

The term retouch is used here in its classic sense: blank modification after the blank detachment. In the IUP layers of Bacho Kiro Cave, there are varieties of retouch and probably not all modifications and the resulting edges had their own functional purpose but were the results of further reflaking. After the blanks were retouched by direct “classic” retouch many of them were intentionally fragmented and reworked **(Fig 5: 9)**. Some tools and blanks were reworked for further extraction of smaller blanks being subject of ***redébitage*** (reflaking) [37, 38].

### Segmentation of blades, bipolar knapping and curation of the imported lithic implements

The lithic economy during the IUP at the Bacho Kiro Cave involved remote provenance of both the raw material and lithic tools, and on-site blank curation for intensive butchering and other activities. The dietary emphasis remains consistent across both the IUP and Middle Paleolithic layers, with a primary focus on the utilization of species from various habitats such as Bos/Bison, Cervidae, Equidae, and Caprinae. Large herbivore body parts were deliberately transported to the site, indicating selective choices [64]. A range of bone tool types, both formal and informal, reveals diverse technological strategies for on-site tasks, often centered on animal skin processing, possibly for crafting clothing for low temperatures [59]. The manufacture of personal ornaments, primarily using carnivore teeth, especially from cave bears, demonstrates technological adaptability, complemented by the inclusion of herbivore teeth and small beads [59].

**Fig 14:**
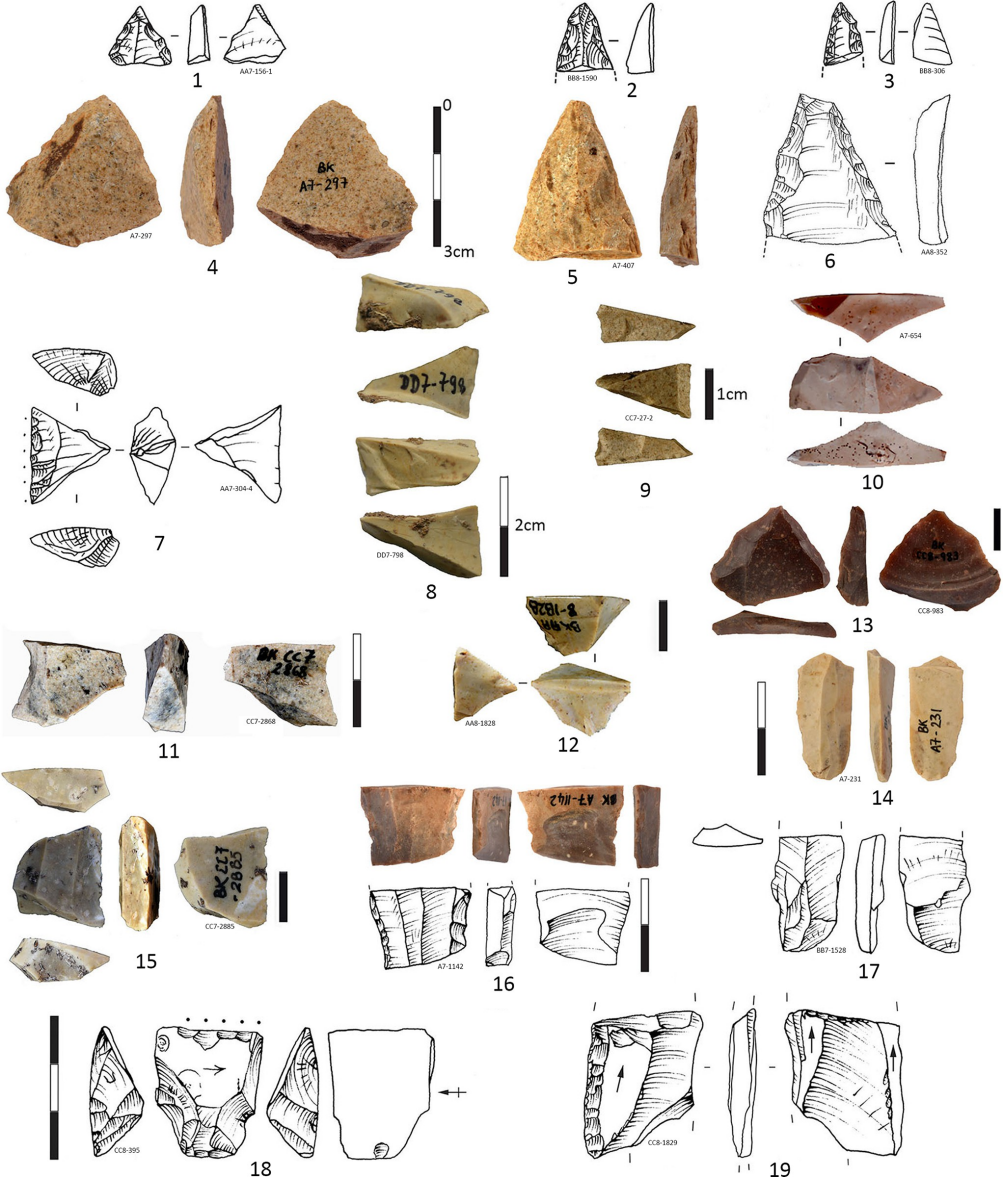
Fragmentation and curations of retouched tools and blades in the IUP layers from Bacho Kiro Cave. **1–3** Short distal tips of retouched points; **4–6** Distal fragments of retouched points (Nr 6 could be convergent sidescraper, Nr 6 is with missing tip, likely in stage of tip resharpening), **7–12** Blade medial fragments of which 7–8, 11 are with bulb on the break attesting deliberate fragmentation; **Blanks and tools with clean breaks:** bulbs (4, 7–11), upper languette (6), lower languettes (13, 16–17), “parasitical” flake (12) or indicative marks of deliberate fragmentation. Typology of breaks according to [71]. Note for Nr 18 and 19 an arrow indicates the debitage direction of the previous surfaces while the last removals (*redébitage* or reflaking, reshaping) are drawn with ripples (Pictures T. Tsanova, Drawings 1–3, 7, 16–19 T. Tsanova, 6 I. Krumov).

A number of heavy gauged blades and retouched tools were subject of intentional fragmentation (visible on 48 break fragments, **S6 Table**), reshaping and re-flaking **(Figs 6, 11)** by bipolar knapping. The 48 artefacts wearing percussion marks and bulbs on the break which attest a deliberate blanks and tools fragmentation **(S7 Table)** as identified in the study of the Bachokirian collection from the previous excavation [38]. Such fractioned marks and bulbs are identified on 26 blade blanks and flakes fragments (2.26%) and 22 retouched tools (6.5%) on various blanks, mostly on medial and proximal fragments **(Fig 14)**. Identified bulb on a breaks concern in 45.8% retouched tools on blades **(S7 Table)**. The deliberate fragmentation does not always leave a bulb or other indicative features [65]. Also, part of the fractures for the thinner blanks and tools could have occurred by tramping [66]. There are in some cases lower face languettes described in [65] and also “parasitical” flakes detached between two opposite languettes **(Fig 14: 12)** and resulting from deliberate blade fragmentation. Other medial blade fragments of triangular shape wear series of cracks in the break zone and likely result from deliberate breakage of medial triangular blade segments **(Fig 14: 8)**. It seems blanks and larger tools were fragmented with purpose to turn them into small cores, and for tool resharpening, even possibly in some cases for manufacture of smaller new tools **(Fig 14: 18)**. Shorter tips of pointed blades suggest some of the deliberate fragmentation results of tool resharpening **(Fig 14: 1–3)**. Such an intentional fragmentation of blades is documented in the IUP site of Kara-Bom (Russian Altai) [67] and possibly at Tolbaga site [68, 69] while for other IUP site of Shuidongdou (North China) the blade fragments are proved to be resulting from accidental breakage [70].

When compared the fragments distribution between the retouched blades (tools) and unretouched blades **(S10 Fig)** is evident that distal fragments are overrepresented among the retouched blade tools and underrepresented among the unretouched blanks. The majority of the pointed blades are represented by distal fragments heavily retouched **(Fig 14: 2, 4–6)** and for this reason typologically reminiscent to Mousterian points as suggested for the previous collection [e.g., 38].

The segmentation and reworking of the lithic artefacts likely serve the purpose of extracting additional blanks and creating additional working edges. Bipolar knapping **(Fig 5)** is often applied in Paleolithic assemblages as an alternative to free-hand knapping for small raw material volume reduction [60, 71–74]. On the other hand, scaled pieces can also reflect a re-use of former tools, cores and blanks as wedges for splitting and processing hard organic material such as bone, wood and antler [72, 75–79]. Here, blanks, cores and fragments show signs of such curation: bipolar knapping is represented by almost 10% of the artifacts, the free-hand cores, the heavily retouched blades and tools **(Fig 7: 7)**, the fragmented tools in various reduction stages **(Figs 11, 13, S10 Fig)**, the small blades **(Figs 5: 10, 12–14; 6: 15–17)** and flakes **(Fig 6: 2–4, 6–8, 10)** produced on-site, the retouch flakes and manufacturing debris (~20% of the assemblage). The multifunctional character of the retouched tools [80], as well their high morphological diversity are indicative of their curation and longer use-life [54].

### Retouched tools: An ambiguous quantity and variability

The intensive blank reduction, fragmentation, large quantity of bipolar pieces, *redébitage* products, tool reshaping and resharpening byproducts allow to explain the initially documented typological variability [33]. A significant portion of retouch flakes and chips (>10%) results from the reshaping and reduction of large blanks and tools, as well as from *redébitage* **(Table 6, Figs 6, 11)**. Our analysis encompasses relatively conventional typological specimens **(Table 11)**. Retouched flakes (34.8%) and blades (25.7%) are the most common tools, followed by retouched pointed blades (12%) and endscrapers (8.57%). Retouched pointed flakes (0.86%) align with the typology of pointed blades. Denticulated-notched tools (3.71%) and sidescrapers (2.28%) are less frequent, although pointed flakes and pointed blades can be classified as convergent sidescrapers. Tool fragments, with a high overall fragmentation rate, constitute 10% of the tools. Perforators, burins, Levallois flakes, truncated pieces, and raclettes are sporadically present **(Table 11)**.

**Table 11:** Tool typology in terms of their blanks in the IUP layers I and J from Bacho Kiro Cave.

Tool typology	Core	Crested blade	Tablet	Plunging flake	Blade	Blade or flake	Bladelet	Burin spall	Elongated flake	Flake	Levallois flake	Scaled piece	Tool fragment	Undetermined.	Total	%
**Endscrapers**					9	2			1	12				6	30	8.57
**Perforator**					1					1					2	0.57
**Burin**					1										1	0.29
**Retouched pointed blade**					42										42	12
**Retouched pointed flakes**										3					3	0.86
**Levallois point**										1					1	0.29
**Truncation**					1										1	0.29
**Retouched blades**		1			84	1	1	2							89	25.43
**Retouched flakes**			1	1		1			4	109	1	1	3	2	123	35.14
**Denticulaited, notched tools**					3				1	8	1				13	3.71
**Sidescrapers**	1				1				1	4			1		8	2.28
**Raclettes**										1	1				2	0.57
**Tool fragments**					4					4			21	6	35	10
**Total**	1	1	1	1	146	4	1	2	7	143	3	1	25	14	350	100
**%**	0.3	0.29	0.29	0.29	41.7	1.14	0.29	0.57	2	40.9	0.86	0.29	7.14	4	100	

Blades were modified principally in three different shapes: by distal retouching in endscrapers, lateral retouches (in retouched blades), or convergent lateral retouches in triangular elongated tools (pointed blades). Those more frequent shapes of blades and elongated tools are complemented by flakes tools with circular forms such as endscrapers, by also retouched points (on flakes) similar to pointed blades but shorter, and retouched flakes. Sidescrapers and retouched blades are distinguished by the blanks type which is usually a flake for the sidescraper and the nature of retouch, but both types cover principally identic attributes. See online 3D model of such a heavily retouched pointed blade reminiscent to convergent sidescraper (https://skfb.ly/oUtHr). Larger imported blades and smaller blades some of which likely to be produced on-site are used for similar tool morphology suggesting that tool-kits consisted of similar tools with various sizes **(Fig 7)**.

### Retouched blade tools economy

Most frequently tools are manufactured on blades (41.7%) keeping in mind that their number was higher as part of them are transformed into cores or other implements **(Table 11)**. When comparing the retouched and unretouched blades, the blade tools with convergent lateral edges are double more recurrent (31.7%) than unretouched blades with convergent edges (14.7%) showing a clear trend also for convergent pointed morphology among the retouched blades **(S8 Fig)**. Blade tools as the unretouched specimens are mainly with unidirectional parallel scars (75.3%), followed by blades with bidirectional opposites scars (8.2%) and convergent scars negatives (6.9%) **(Table 9, S8 Fig)**. Blades with straight profiles (88.5%) are preferable for being retouched, and those with triangular cross-sections predominate (53.4%). Blades with a trapezoidal cross-section (43.4%) are frequently selected for being retouched as they predominate with almost 10% of the unretouched blades with a trapezoidal cross-section (35%) **(S9 Fig)**. Therefore, there is a certain tendency for robust long tools and this trend is even more clear with the blades with polyhedral cross-sections: from the blade tools 3.17% with polyhedral cross-sections against the remaining 1.41% unretouched blades with the same section **(S9 Fig)**. See an online 3D model of pointed heavy blade (https://skfb.ly/oUtHB).

## Discussion and comparisons

The Initial Upper Paleolithic (IUP) has been one of the most widely discussed topics in Paleolithic archaeology over the past two decades. The record from Bacho Kiro Cave provides crucial data for discussing the early dispersals of *H*. *sapiens* during MIS 3 and interactions with indigenous Neanderthals, the precise chronology and regional duration of the occupations, and contribute for understanding the patterns of mobility and logistic organization of substantial activities. It also sheds light on cultural diversity, adaptation strategies, and environmental impacts. Furthermore, it allows for comparisons with other *inter*- and *intra*-regional IUP sites. The IUP lithic assemblages in the region are recognized only in the northern slope of the Balkan Mountain, and stratigraphically they are positioned between the MP and UP deposits. As in the original IUP definition likely derived from a Levallois-Mousterian technology similar to those in south- eastern Asia and the Levant [81, 82]. The IUP was originally defined in the Levant, where it coexisted and emerged from late MP Levallois technology around 50 ka cal BP most likely in the southern Levant [83, 84]. There, the IUP transformed gradually, based on the MP Levallois-Mousterian tradition: from a Levallois laminar technology called Emiran at Boker Tachtit (Level 1–3) to a UP prismatic blade technology called IUP [9, 85].

### Major technological and typological features of the IUP in Bacho Kiro Cave

The analyzed lithic assemblages from Bacho Kiro Cave in their contextual background reflect a certain degree of behavioral plasticity of these *H*. *sapiens* that inhabited between the lower Danube and the Balkan Mountains about ca 45 ka. Massive silicite acquisition from diverse remote sources, off-site core reduction, transformation of blanks and their transport to the site up to ca 190 km can be interpreted as evidence for long distance mobility. Finished products (blades) and other lithic implements were most likely produced and transported from mainly two remote locations: Ludogorie and Nikopol-Asenovo area close to the Danube River. The imported blades exhibit unidirectional and bidirectional laminar reduction and distinct *chaines operatoires*, but resulting in similar final products (large straight blades produced by direct percussion with hard hammer). Some blades, as indicated by their technical attributes, originate from volumetric cores with concepts that are usually present in Levallois technology (facetted platforms, bidirectional and convergent scar-pattern). The long-distance imported blanks and finished products were subject of secondary knapping in the cave and served for the planned activities related to exploitation of the fauna, producing of bone tools and ornaments. As a result, the lithics are highly fragmented, decreased in size, and transformed into more or less typical scaled pieces. That is, a proportion (perhaps quite a large, if not most) of these scaled pieces is not the result of a purposeful, deliberately applied bipolar technique, but rather (unintended) of other operations (activities).

Blade and flake tools and blanks were used for on-site activities [38] then resharpened, reshaped as indicated by the large number of chips and retouch flakes [12, 37] and re-flaked on-anvil percussion and turned into small cores and wedges [60]. The intense on-site activities and use of the cave during the formation of Layer I can be deduced from the increased density of finds a large number of piece-plotted fauna and the human modifications on the fauna [64], evidence for the extensive presence of fire, growth of anthropogenic input of organic matter [14] and sieved sediments extremely rich in bone fragments and tiny lithic (< 1.5 cm). This intensive lithic reduction was likely related to processing animal remains, as confirmed by high number of selectively transported fauna [64], and subsequent bone processing for the manufacture of osseous tools, ornaments, and animal- teeth pendants [59]. Small blades and flakes produced on-site were probably used unmodified for wood working [80]. Blade tools and blanks were intensively reshaped and used (with some of their edges damaged from this use). Different types of tools were used on similar materials and for similar tasks [80], showing their multifunctional aspect and nonspecialized function. On-site lithic and bone technologies were interactive and complementary [60]. Different types of retouched blades seem to have been designed and used intensively (e.g., long-term use) to work different materials, including bone [80]. Similar economy and functional patterns are observed in the bone technology where bone tools and ornaments where crafted with lithic tools [59]. It seems that a significant amount of fauna is processed with comparably low quantity of lithic raw material. In Layer I there are almost 14 bones (> 2 cm) compared to only 2 lithics (> 1.5 cm) per liter of sediment. Bone per liter of excavated sediment is 17.5 times greater in Layer I than in Layer J [64]. The increasing density of finds, coupled with the heavily retouched and reduced lithics, strongly indicates the potential transformation of the site into a dynamic hub for meticulously planned task executions [54, 86]. Drawing on L. Binford’s concept of gear organization, which is intricately linked to "special purpose" locations, the notion of an active gear emerges as a distinctive settlement-subsistence system shaped by a unique technological organization [54]. The major techno-typological and economic features inherent in the IUP lithic artifacts assemblages in Bacho Kiro Cave summarized below, outline behaviors suggesting that the site may have functioned as an active gear.

Major technological and typological features of the IUP assemblages in Bacho Kiro Cave:

- Long distance (up to 190 km) transport of finished products from two distinct areas;
- Primary off-site distinct *chaînes opératoires* for blades with similar final products: straight profiles, robust triangular and trapezoidal sections, uni- and bidirectional scar pattern, parallel edges, plain and also facetted platforms;
- One of the off-site reduction methods for blades was likely bidirectional with shifted adjacent flaking platforms and initiated at the narrow and wide core sides.
- Diagnostic retouched tools are pointed blades, some of them reminiscent of Mousterian points and sidescrapers are heavily retouched;
- UP tool types: endscrapers, retouched blades, burins resulting from bipolar percussion;
- Secondary on-site knapping: bipolar technique applied on blade fragments and other fragmented tools for wedging activities and, also for production of small blades and flakes (re-debitage);
- Deliberate fragmentation and reutilization of previous large blade tools;
- Small blades and flakes produced on-site are not retouched and likely used with raw edges;
- On-site production and use of bone tools and personal ornaments;
- Increasing the number of finds with each subsequent occupation which implies more activities (perhaps also more people).

These techno-typological features typical for the IUP assemblages from Bacho Kiro Cave are not unique to this cave and they have also been described for other IUP assemblages in other regions (see below). This widespread occurrence suggests that similar adaptive strategies were employed by *H*. *sapiens* across diverse environments.

Direct evidence for the climatic conditions during the IUP occupations in Bacho Kiro Cave was obtained through high-resolution oxygen-stable isotope analysis of bison and horse teeth [40]. *H*. *sapiens* experienced subarctic cold conditions, with mean annual temperatures 10–15°C lower than today. For Layer J, the mean annual temperature was -1.1 ± 3.3°C, similar to present-day Russia and Central Asia, while for Layer I, it was slightly higher at 3.4 ± 2.5°C, similar to present-day Scandinavia [40]. These findings align with the presence of cold-climate species like woolly mammoth, reindeer, and wolverine [64]. Seasonality data from Layer I indicate year-round habitation of the cave [64]. Recalibration using Radiocarbon 3.0 of directly dated human remains **(S1 Appendix in S1 File)** suggests that humans primarily lived in the colder phase of GI12 (Greenland interstadial) and possibly at the end of its warm phase. A model with three occupational phases indicates that two humans inhabited the cave during the warm phase of GI12, one during its cold phase, and another at the beginning of the warm interstadial GI11 [87].

The refined results with Radiocarbon 3.0 and the possibilities that humans were in the cave during generally cold but also warmer climatic conditions is more in agreement with the fauna composition. Mix of macro and microfauna species are indicative for local and regional climate shifts and suggest that the Late Pleistocene climate around Bacho Kiro Cave was cooler than today with diverse environmental conditions [14, 33]. During the last ice age, the Balkans’ climate was warmer than central and western Europe [88, 89]. The Bacho Kiro Cave, in a semi-mountainous area, was a natural habitat for various caprines and close to forested and mixed environments, as well as northern steppic areas. The climatic data indicate that IUP *H*. *sapiens* from Bacho Kiro Cave inhabited and adapted to generally cold climate with certain fluctuations, and he was hunting in divers’ environments. His subsistence focused on a range of large- to medium-sized herbivores, along with opportunistic hunting or exploitation of natural deaths of smaller and larger carnivores, including cave bears [64]. The exploitation of carnivores, especially cave bears, appears to be related to using these species for fur, as well as raw material for bone tools and personal ornaments [59, 64].

### Bacho Kiro IUP within the regional context

The lithic assemblages from Bacho Kiro Cave are interpreted since the 1970s as being locally intrusive mainly on the basis of raw material shift between MP (local metamorphic rocks) and UP layers (imported silicites) [33]. From a regional perspective, looking at the blade technologies and lithic tools we find general similarities with Temnata Cave located ca 140 km to the west and Samuilitsa II Cave. Two sectors in Temnata reveal human occupations as possibly of IUP with blade technologies and UP tool types. Here, the lithic record in sector II-layer VI made out mainly of local raw material, contains Levallois and volumetric prismatic blade components (including hard-hammer percussion and faceting of platforms), consistent with the IUP record. However, the stratigraphic association of both components was contested [90]. Nevertheless, the blade volumetric component is analogous to the one from the IUP. Mostly bidirectional and also unidirectional reduction was used for production of rectangular large-size blades by direct percussion with a hard hammer [12]. This layer VI is covered by CI-Y5 tephra, indicating that it is older than 39 Ka BP [42]. In sector I, layer 4 includes blade assemblages with similar UP prismatic-volumetric cores and UP tool types. Within the same area there are Samuilitsa II and Toplitsa caves with chronologies and technologies corresponding to the IUP *sensu lato* **(S6 Appendix in S1 File, S11 Fig)**. In Samuilitsa II the Levallois technology is decreasing towards the top of the sequence where it is replaced by UP prismatic blade cores [91, 92].

On the other hand, the main difference between the Bacho Kiro Cave and the other sites in northern Bulgaria is in the use of non-local flint sources, high transportation of blades and high curation of blanks and tools. In Temnata and Samuilitsa II, the assemblages are predominantly made from raw material that is local and, therefore, there was presumably no need for tool curation (**S8 Table**). Interestingly, here is a similar pattern of increasing density of artefact distribution within the sequence towards the top of the layer 4 in Temnata [38] suggesting increasing human activities as in Bacho Kiro Cave.

In Kozarnika Cave, which contains the most complete regional Paleolithic sequence, the layer corresponding chronologically to the IUP is the layer 6/7 located between the MP and UP layers [12]. The lithic assemblage from this layer has two or three technological components: one laminar, likely corresponding to the IUP, and the other is lamellar, technologically corresponding to the overlying Early Kozarnikian from the layer 5c (level VII), and possibly some Middle Paleolithic input. Due to their potential of significantly complementing and increasing our knowledge about the earliest *Homo sapiens* and IUP of the region, a continuous and renewed work is needed in these other caves, and also the collections.

### Bacho Kiro IUP within the broader IUP context

Several basic technological elements such as the use of hard-hammer direct percussion for blade production, facetted platforms, and planar core exploitation allow the attribution of the Layer I and J lithic record as a variant of the Eurasian IUP as defined by [11]. Moreover, the sudden regional appearance of blade technology associated with bone tools and ornaments [11] and UP tool types **(S8 Table)** strengthens the affiliation with the IUP. There is substantial variation among the Eurasian IUP record: certain technological features existing in one IUP site can be nonexistent in another site [11], as the broad geographical range of the IUP entailed exploitation of various biogeographic zones and stone resources across almost ten thousand years by different human populations [7, 13], in which case it is arguably unreasonable to expect a complete technological homogeneity and entirely identical artifact collections. The IUP record from Bacho Kiro Cave shares a common pattern in unidirectional production of blades with parallel edges with Üçağızlı cave [93], for example, but they both differ in the debitage concept of the Bohunician record where convergent Levallois blanks are manufactured on the narrow face of non-Levallois core [94]. The Bohunician technology differs from the IUP in Layer I in Bacho Kiro Cave in its production of typological elongated Levallois points, which are detached from bidirectional narrow facetted cores that transform into unidirectional and flat-facetted cores at the end of the reduction process [61]. The blades detached from the core sides *(débordants)* are byproducts of the manufacture of predetermined elongated points with Levallois morphology [94]. Bohunician technology is interpreted as a conceptual fusion between Levallois and volumetric laminar methods [95]. In the IUP of Bacho Kiro Cave, there are also a few Levallois flakes likely products of *rédebitage* and a unique small Levallois core **(Fig 6: 4–10)**, but these are only partially comparable to the Bohunician due to their infrequency and the clear absence of elongated Levallois points. This conceptual difference in elongated points versus blade production between the Bohunician and Bacho Kiro Cave could be also because the Bohunician represents an older IUP phase. The production of Levallois elongated points from bidirectional non-Levallois method is typical for the early IUP development (Emirian) in Boker Tachtit (Layer 1–3) [9] while the laminar production associated to volumetric pyramidal cores is typical for the late IUP phase (corresponding to the uppermost layer 1 at Boker Tachtit) [11, 21]. The Ukrainian open-air site of Kulychivka site [96, 97] share similar conception of blade production like the Bohunician but with relatively recently obtained younger radiocarbon ages [98]. In any case, a robust technological link is established between Bohunician and the Levantine IUP [99].

Lately, a re-examination of Lincombian-Ranisian-Jerzmanowician (LRJ) industry from five sites in southern Moravia and two caves in Bohemia (Czech Republic) are actually reconsidered as late IUP industry being dated shortly before the Heinrich 4 Event and the Campanian Ignimbrite (CI) super-eruption, c. 42–40 ka cal BP [21]. The blade technology at these sites is interpreted as being evolved from the Bohunician and it consists in general of non-Levallois uni-and bi-directional blades, and elongated flakes used as blanks for J-type unifacial and partially bifacial points, UP tools types, and in some case preserved personal ornaments [21]. This re-attribution of the LRG techno-complex is confirmed by new discoveries at the site Ilsenhöhle in Ranis consisting of new directly associated *H*. *sapiens* remains [18]. These new findings show that LRG at Ranis is made by humans with *H*. *sapiens* mtDNA much earlier than Neanderthal went extinct, and with a genetic link to the Zlatý kůň *H*. *sapiens* skull from Check Republic, the last overlapping with dates for the Bohunician [18, 100]. These new findings and the re-attribution of the LRG techno-complex (previously attributed to Neanderthals) demonstrate that technical systems, such as blade technology and production methods, were evolving and dynamic aspects of human technological behavior. This evolution was influenced by inherited techno-cultural traditions and environmental contexts [101]. Shared technological and typological features in laminar and retouched tools production from the Mediterranean and Central European IUP sites can be acknowledged together with certain differences. One part of the blades from Bacho Kiro Cave originated from bidirectional cores with opposite and shifted platforms. In southern Turkey Üçağızlı cave, in layers from G to I, preserves a rich sequence with IUP occupations dated ca 45,000–43,000 comparable to the IUP from Boker Tachtit 4 and Ksar Akil sites [93]. The volumetric method of unidirectional reduction and techniques of laminar production at Üçağızlı are comparable with those from IUP layers in Bacho Kiro and Temnata caves (except for the middle part of layer 4 in Temnata (phase B) where the blade production is predominantly bidirectional [12]. Unlike in Bacho Kiro Cave at Üçagızlı cave the silicite originate from not-so-distant sources [93], therefore we can expect different on-site lithic economy between both IUP sites. In the case of Bacho Kiro Cave more logistical anticipation is needed to supply the cave with the designated silicite quality. Shared feature between Üçağızlı IUP layers and Bacho Kiro cave is the pattern of occupation with less intensive find density at the base and increasing toward the top [93] which is also coinciding with the distribution of finds pattern in Temnata cave, Layer 4. In the site of Ksâr ‘Akil site (Lebanon) the blades production of IUP levels XXV to XXI typical with unidirectional reduction of volumetric prismatic cores and UP tool type [102] suggesting comparable concepts and reduction technique with Bacho Kiro site, but also Temnata, Layer 4 (lower phase C and upper phase A). IUP at Ksâr ‘Akil began before 45,900 cal BP [103] and it is suggested now that previous stages of the IUP development are not presented at the site [104, 105]. Finally, a similar chronological schema of development is observed in the Negev, where the later IUP overlaps with the EUP Ahmarian of the Mediterranean region, between 47 and 44 ka calBP [83]. The coexistence of IUP and EUP industries in the Levant precedes the similar process in the eastern Balkans by a few millennia, and most probably led to the dispersals of human groups and technological knowledge from South-western Asia and the Levant to Europe [4, 17]. Recently it has been proposed that the IUP of Bacho Kiro is corresponding to the Early Upper Paleolithic (EUP)/ Norther Early Ahmarian (NEA) of the Levant, characterize by production of small bipolar blades and backed points [25]. The IUP of Bacho Kiro assemblages are lacking bladelets *sensu-stricto* and backed points (among other differences). What corresponds to the Levantine EUP / NEA is the Early Kozarnikian, Level VII [106], as explained above.The lithic assemblages from Bacho Kiro Cave share some of the major features of blades technologies and behaviors from Siberia and Central Asia, whose appearance goes back > 50 Ka BP [107, 108]. The IUP in this regions follows Mousterian and Levallois-Mousterian layers in Denisova cave, Okladnikov, Kara-Bom [109] and could have evolved based also from the local Middle Paleolithic as is roughly contemporaneous to the Levantine IUP [83, 107, 108]. In Altai (Siberia), there are several well-known IUP sites include Kamenka and Varvarina Gora sites, Khotyk, Barun-Alan sites, in the Zabaikal region, and the Ust-Karakol and many others that yielded laminar technology and UP tool-types typical [109, 110].

Exceptionally long distance transportation of raw material over 500 km have been reported in the IUP site of Kharganyn Gol 5 (Northen Mongolia) [111]. In the site Tolbor-16 and Tolbor-21, the IUP techno-complex appeared between 45 and 40 ka cal BP [19, 112]. The IUP in Central and East Asia is consistent with *Homo sapiens* dispersals along a ‘Northern Route’ as early as 46–45 ka cal BP [16, 17, 109, 113, 114]. A part the chrono-stratigraphical context of the IUP, the technological features distinguishing those assemblages are large blade production, reduction of thick technical elements into burin-cores, platform preparation by pecking, and presence of personal ornaments [112]. The IUP site of Tolbor-16 is contemporaneous with *H*. *sapiens* skeletal evidence [16] establishing a crucial archaeological link between Siberia and Northern China [19]. Along with evidence from adjacent regions, the material from Archeological horizon 6 at Tolbor-16 suggests a widespread behavioral shift occurred across the Eurasian steppe during a period of climatic instability included milder and wetter phases, and potentially increased the landscape’s carrying capacity and facilitated human expansions [19]. The timing of the IUP and *H*. *sapiens* dispersal in this part of Asia coincides with the millennial-scale GI12 temperate climate event (ibid.).

In northwestern China, one of the most prominent IUP sites is Shuidonggou Locality 1 and 2 contains blade-rich assemblages and has been extensively studied. At Shuidonggou 2 blade technology associated with personal ornaments appeared around 41 ka cal BP [115] in the time core-and-flake assemblages were widespread in North China [116]. The site of Shiyu site in Northen China delivered an assemblage in Layer 2 consistent with IUP (volumetric blade cores and Levallois points), long-distance transfer of obsidian from even longer distances (China and Russian Far East 800-1000km away) and dated back to ca 45 Ka [117]. Few *H*. *sapiens* fossils have been recovered over a wide geographic area of northern Asia, with Ust’-Ishim in the Russian Altai of southern and western Siberia representing one of the earliest *H*. *sapiens* in the region, dated to approximately 45.9–42.9 cal ka BP [16].

By ca 40 Ka BP *H*. *sapiens* was presented in northeastern China in Tianyuan Cave site which site provided human fossils and ancient DNA evidence (ibid.) which ancestry was subsequently widespread in Eastern Asia [117, 118]. In summary, multiple IUP sites have been discovered in both Siberia and China, contributing to our understanding of Late Pleistocene *H*. *sapiens* dispersals and technological advancements in these regions. Regardless of the long distance, certain conceptual and behavioral shared features between the record from the Asian sites and the Bacho Kiro Cave can be acknowledge:

- simultaneously appearance of IUP in the time-span of 46–45 Ka BP;
- blade volumetric technologies (bidirectional but also unidirectional) associated with UP tool-types, production of small blades and burin-cores, personal ornaments;
- certain assemblages oriented toward the production of convergent blades and triangular flakes, with facetted platforms, typologically Levallois and produced from volumetric cores of UP concept;
- dominance of hard hammer direct percussion technique;
- increased long-distance mobility and transportation of raw material and lithic implements;
- IUP is mostly intrusive in North China and Mongolia, as its appearance in Bacho Kiro Cave, but not in the places where is stratified above Levallois-Mousterian (likely the case of Samuilitsa II cave near Temnata Cave in Bulgaria);
- IUP occurring in a time of generally cold climatic conditions with certain instabilities.

Lastly, the IUP attribution of Layer I and J in Bacho Kiro Cave was contested, assuming laminar production follows the Levallois method [26]. The critique highlighted three misconceptions, regarding blade production methods, personal ornaments and comparisons with Üçağızlı cave: 1) New excavation results yielded complete blades **(Figs 7; 8: 2)**, supporting evidence that blades originated from prismatic volumetric and bidirectional pyramidal cores with shifted platforms (**Figs 7: 1, 4; 8: 7)**. The absence of clear Levallois blades and the presence of a large partial tablet **(S12 Fig)** inconsistent with the Levallois concept supports the argument for volumetric blade debitage; reworked small cores are exploited on the wide face and the narrow edge **(Fig 6: 1, 11)**. Unretouched blades are with abrupt lateral edges **(Figs 7: 5; 8: 2, 3, 7, 9)** contradict the contesting paper´s claim of flat blades; 2) Considering personal ornaments, regional IUP variations are crucial. In the Balkans´ continental inward zone, IUP layers often deliver pendants and ornaments made from locally available resources [33], aligning with the definition that IUP personal ornaments reflect local resources [11]; 3) Regarding the comparison with Üçağızlı cave Layer I-F and the IUP layers in Bacho Kiro Cave [11, 14, 38], given the diverse environmental context of *H*. *sapiens* dispersals, naturally lead to variations in lithic technology and behavioral economy. These variations are not only expected but also underscore the adaptability and resilience of IUP *H*. *sapiens*.

## Conclusion

Bacho Kiro Cave provides one of the most detailed contexts for the IUP, offering precise insights into the behavioral variability and adaptation among IUP groups in southeastern Europe. The archaeological assemblages from Layers J and I, including those from both the Main sector and Niche 1, as well as layers 11 and 11a from the 1970s excavation, represent a variant of the Eurasian Initial Upper Paleolithic phenomenon showing a specific nature of environmental adaptation and exploitation of available resources. With exceptionally precise and accurate radiocarbon chronology, Bacho Kiro Cave is crucial for understanding the duration of the IUP, accurately dated between 45,040 to 43,280 cal BP, beginning likely around 45,990 cal BP [6], recalibrated using IntCal20). A new model suggests the IUP lasted either 1710 years or 770 years [87].

*H*. *sapiens* from Bacho Kiro Cave show genetic links to Neanderthals, demonstrating that when they entered the Balkans they interacted with them, moreover they contributed to present-day and ancient populations in East Asia and the Americas, and not to west Eurasian populations [13]. Bacho Kiro Cave genomes show that several distinct *H*. *sapiens* populations existed at the onset of the UP in Eurasia.

The laminar technology widely utilized during the IUP was characterized by the production of robust and long blades using direct percussion with a hard hammer, was vital for adapting to the cold and unstable climate. These humans were transporting high-quality flint and using imported tools for on-site activities, including butchering herbivores and carnivores like the cave bears and other carnivores likely exploded for their fur and manufacture of warm clothing [59]. Tools were segmented, reused, and adapted for working on various materials [80] and manufacturing osseous formal and unformal and informal tools and pendants from animal tooths (mainly cave bear) [59]. The crafting of personal ornaments, beads from hard animal tissue and stone, engraved bones, and also the use of ocher highlight the cultural identity of these hunter-gathers groups (ibid.).

The increased activities in the IUP layers at Bacho Kiro Cave and also Temnata suggest a demographic impact in the region and raise questions about possible interactions with new *H*. *sapiens* groups, such as the makers of the Early Kozarnikan bladelet techno-complex, whose radiocarbon ages overlap with the IUP. Additionally, this prompts questions about the factors that led the *H*. *sapiens* makers of the IUP to leave the region and disperse towards Asia [12].

Future research should focus on processing more sites with high-resolution radiocarbon dates to better understand the trajectory of the IUP techno-complex in southeast Europe, its connections to the Levant, southwest Asia, and central Europe. Detailed studies on post-excavation stratigraphy [20], spatial distribution of raw materials, and raw material economy will enhance our understanding of the technological organization and variability, behavioral adaptation, and potentially social organization of these early human groups.

## Supporting information

S1 Fig Bacho Kiro Cave, raw material–Group 11. 1. Macroscopic view; 2 à 6. Mesoscopic view. Bryo: cyclostome bryozoan; inc: Incertae sedis; Text: benthic foraminifera cf. Textularidea; Rota: benthic foraminifera cf. Rotalidea; Glom: benthic foraminifera cf. Glomospira. Group 16: 7. Mesoscopic view.
(TIF)

S2 Fig Bacho Kiro Cave, raw material–Group 13. 1. Macroscopic view; 2 à 7. Mesoscopic view. Bryo: cyclostome bryozoan; Glomo: benthic foraminifera cf. Glomospira; coq: fragment of undetermined shell; qua: detrital quartz grain.
(TIF)

S3 Fig Bacho Kiro Cave, raw material–Group 21. 1. Macroscopic view; 2. Mesoscopic view. Group 22: 3. Macroscopic view; 4. Mesoscopic view. Group 23: 5. Macroscopic view; 6. Mesoscopic view. Group 24: 7. Macroscopic view; 8. Mesoscopic view.
(TIF)

S4 Fig Bacho Kiro Cave, raw material–Groupe 33. 1. Macroscopic view; 2 à 7. Mesoscopic view. Spim: monaxon spicule of Hexactinnelide sponges; Rota: benthic foraminifera cf. Rotalidea; radi: spumellar radiolarian; serp: worms tubes cf. serpulidae.
(TIF)

S5 Fig Bacho Kiro Cave, raw material–Groupe 34. 1. Macroscopic view; 2. Mesoscopic view. Groupe 35: 3. Macroscopic view; 4 à 6. Mesoscopic view. Groupe 36: 7. Macroscopic view; 8. Mesoscopic view. Dasy: Marine green algae cf. dasycladale; bryo: cyclostome bryozoans; Lent: benthic foraminifera cf. Lenticulina; spon: fused spicules of sponges.
(TIF)

S6 Fig Bacho Kiro Cave, raw material–Groupe 41. 1. Macroscopic view; 2. Mesoscopic view. Groupe 42: 3. Macroscopic view; 4 à 6. Mesoscopic view. Groupe 51: 7. Macroscopic view; 8. Mesoscopic view. Glob: planktonic foraminifera cf. Globorotalidea; coq: fragment of undetermined shell.
(TIF)

S7 Fig Scatter-plot of cores dimensions: length and width (left) and width and thickness (right) of freehand and bipolar cores, IUP layers, Bacho Kiro Cave.
(DOCX)

S8 Fig Clustered column comparing the shape of blades lateral edges between unretouched blades and blade tools, IUP layers, Bacho Kiro Cave. (DOCX)

S9 Fig Clustered column and bar comparing the blade profiles types of unretouched blades and blade tools (left) and blade cross-section types (right), IUP layers, Bacho Kiro Cave.
(DOCX)

S10 Fig Comparison of the distribution of blade fragments (distal, medial, and proximal) for retouched and non-retouched blades in relation to complete blades. (DOCX)

S11 Fig Regional chrono-technological comparisons [12]. Plotted calibrated radiocarbon dates from Kozarnika, Temnata, Bacho Kiro and Samuilitsa II caves. The original dates from Kozarnika and Temnata caves are on charcoal, while those from Bacho Kiro Cave are on bone. The single date from Samuilitsa II Cave, made on bone, is 42,780 ± 1270 uncalBP (GrN- 5181). All dates are recalibrated, using Oxcal v.4.4 with IntCal20 curve [119].
(DOCX)

S12 Fig Large partial tablet from IUP layer I in Bacho Kiro Cave, and corresponding likely to the volumetric laminar technology (Pictures Ts. Tsanova, drawings I. Krumov). (TIF)

S1 Table Counts of piece-plotted lithic (>1.5 cm), fauna remains (>2 cm), and collected and sieved sediments (1 bucket contains 9 liters of sediment) in the IUP layers from Bacho Kiro Cave. (DOCX)

S2 Table Bacho Kiro—Synthetic table of silicite groups. (-) = rare; (o) = present; (+) = abundant; (+++) = very abundant.
(DOCX)

S3 Table Bacho Kiro—Geological variation of different material groups. On the left, silicites; on the right, other rocks.
(DOCX)

S4 Table Core blanks for the bipolar and freehand cores in the IUP layers from Bacho Kiro Cave. (DOCX)

S5 Table Comparison of blade platform types between the IUP assemblages from Bacho Kiro (Layers I and J), and Temnata caves (layer 4). (DOCX)

S6 Table Distribution of retouched tool fragments in the IUP layers from Bacho Kiro Cave. (DOCX)

S7 Table Counts of the artefact fragments with percussion marks and bulbs on the break attesting a deliberate fragmentation in the IUP layers from Bacho Kiro Cave. (DOCX)

S8 Table Comparison with regional IUP blades and EUP bladelets assemblages. Techno-economic features. (DOCX)

S1 File References. (DOCX)
