# Correction: Curated character of the Initial Upper Palaeolithic lithic artefact assemblages in Bacho Kiro Cave (Bulgaria)

**DOI:** 10.1371/journal.pone.0347680

**Published:** 2026-04-17

**Authors:** Tsenka Tsanova, Vincent Delvigne, Svoboda Sirakova, Elka Anastasova, Pedro Horta, Ivaylo Krumov, João Marreiros, Elena Nacheva, Zeljko Rezek, Jean-Jacques Hublin, Nikolay Sirakov

In Fig 9, the legend of the fourth group in yellow should be “EPA bipolar cores” instead of “Exterior platform angle (degree)”. Please see the correct Fig 9 here.

In Fig 10, the legend of the fourth group in yellow should be “Flake platforms thickness” instead of “mm”. Please see the correct Fig 10 here.

In Fig 12, the legend of the sixth group in green should be “Non-retouched blades thickness” instead of “mm”. Please see the correct Fig 12 here.

In the Stratigraphic context subsection of Results, there is an error in the third sentence of the first paragraph. The correct sentence is: The Main Sector (MS) had 246 piece-plotted (cut-off size 1.5 cm) lithics across ca. 3m^2^, for 6417 liters of sediments, while Niche 1 yielded 2435 lithics across ca. 10 m^2^ for 9765 liters of sediments. This numbers can be verified in S1 Table (MS has 713 buckets x 9 liter = 6417 l. while Niche 1 has 1085 buckets x 9 liter = 9765 l).

**Fig 9:**
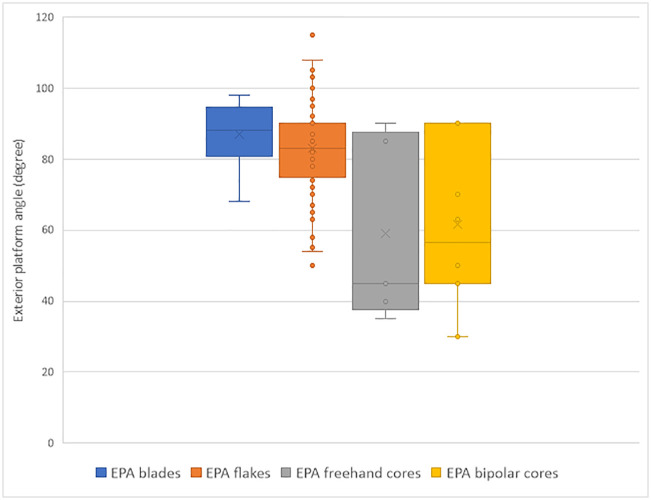
Plot of the exterior platform angles (EPA) of blades, flakes, freehand and bipolar cores, IUP layers, Bacho Kiro Cave.

**Fig 10:**
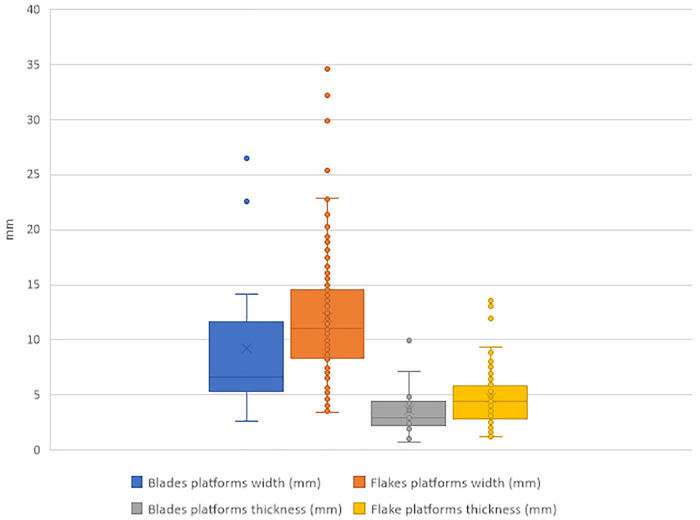
Box & whisker plot of the blades and flakes platforms dimensions (width and thickness), IUP layers, Bacho Kiro Cave.

**Fig 12:**
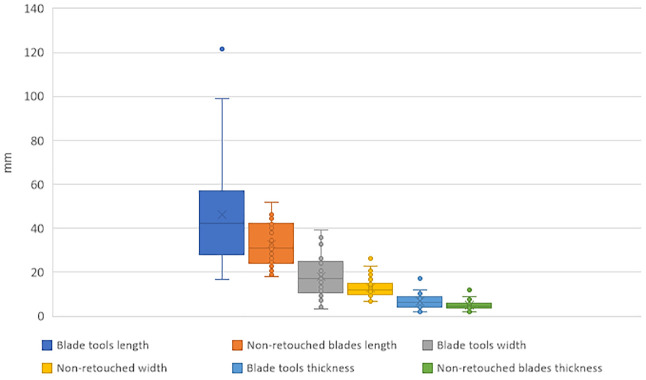
Plot of blades dimensions: Length, width and thickness comparing complete retouched (tools) and complete unretouched blades, IUP layers, Bacho Kiro Cave.
