# Supplementary figures and images for: Curated character of the Initial Upper Palaeolithic lithic artefact assemblages in Bacho Kiro Cave (Bulgaria)
Source: PLoS One. 2024 Sep 4;19(9):e0307435. doi: 10.1371/journal.pone.0307435 (PMC11373871; doi:10.1371/journal.pone.0307435)

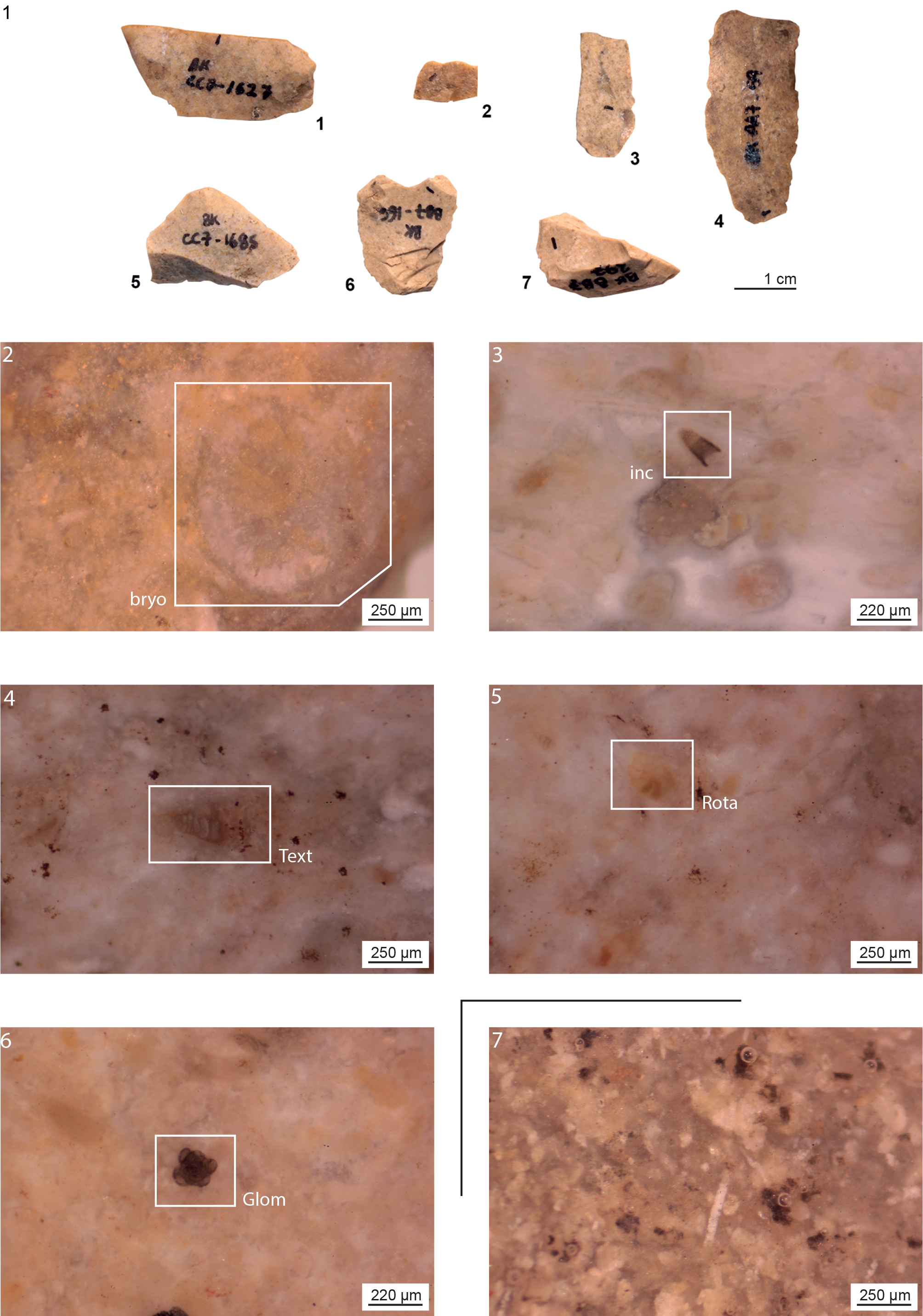

Supplement: S1 Fig — 1. Macroscopic view; 2 à 6. Mesoscopic view. Bryo: cyclostome bryozoan; inc: Incertae sedis; Text: benthic foraminifera cf. Textularidea; Rota: benthic foraminifera cf. Rotalidea; Glom: benthic foraminifera cf. Glomospira. Group 16: 7. Mesoscopic view. (TIF) [file pone.0307435.s001.tif]

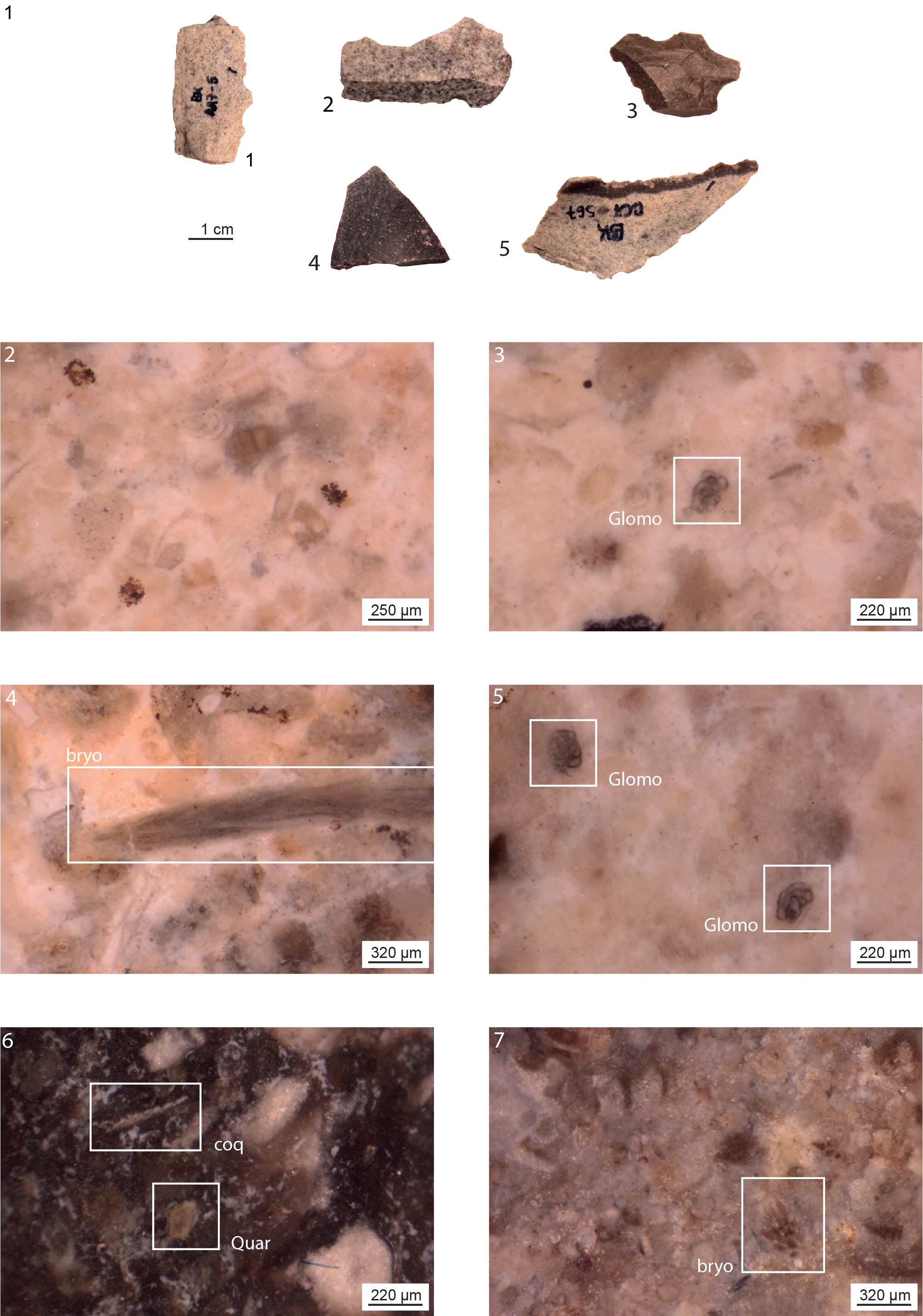

Supplement: S2 Fig — 1. Macroscopic view; 2 à 7. Mesoscopic view. Bryo: cyclostome bryozoan; Glomo: benthic foraminifera cf. Glomospira; coq: fragment of undetermined shell; qua: detrital quartz grain. (TIF) [file pone.0307435.s002.tif]

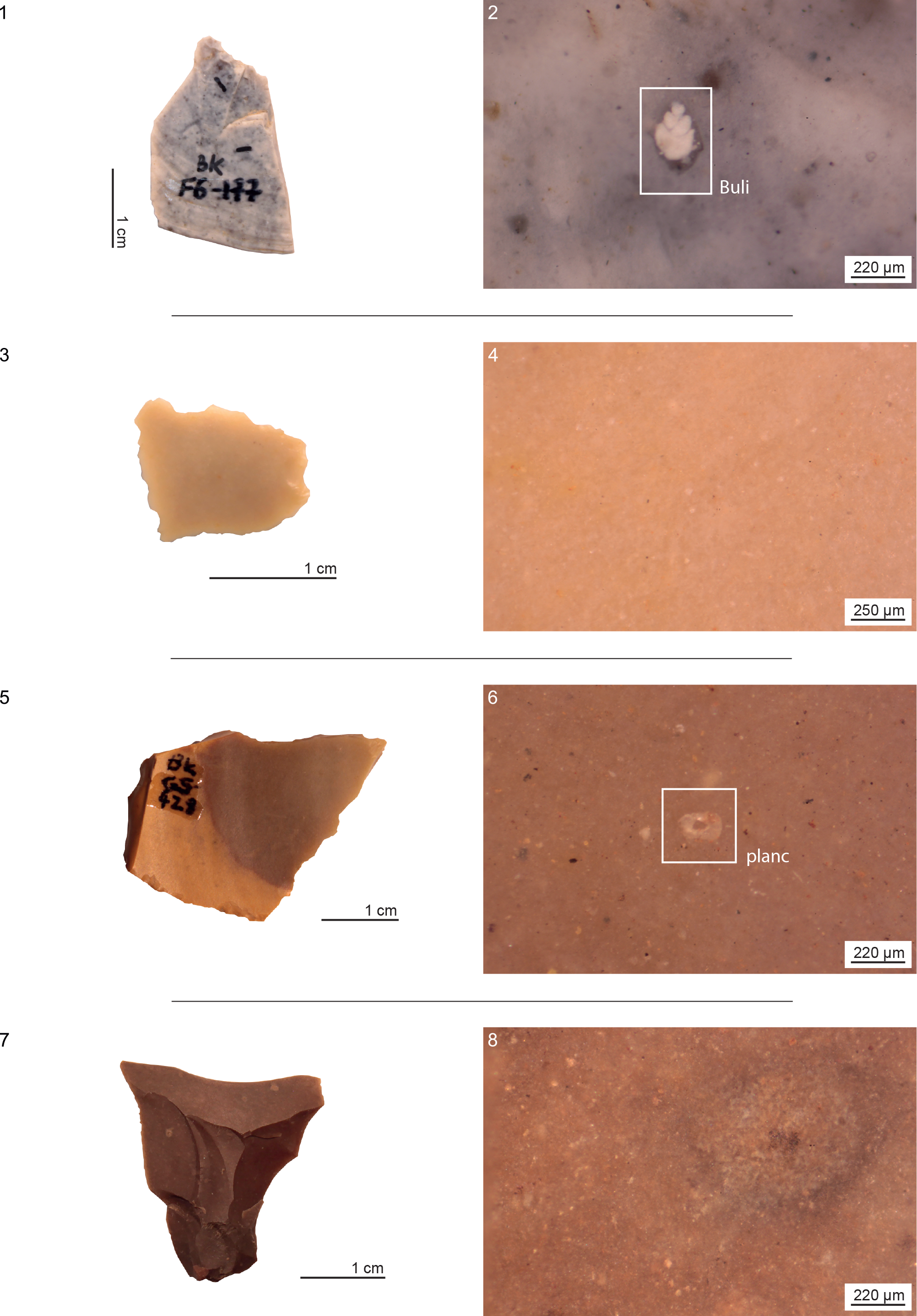

Supplement: S3 Fig — 1. Macroscopic view; 2. Mesoscopic view. Group 22: 3. Macroscopic view; 4. Mesoscopic view. Group 23: 5. Macroscopic view; 6. Mesoscopic view. Group 24: 7. Macroscopic view; 8. Mesoscopic view. (TIF) [file pone.0307435.s003.tif]

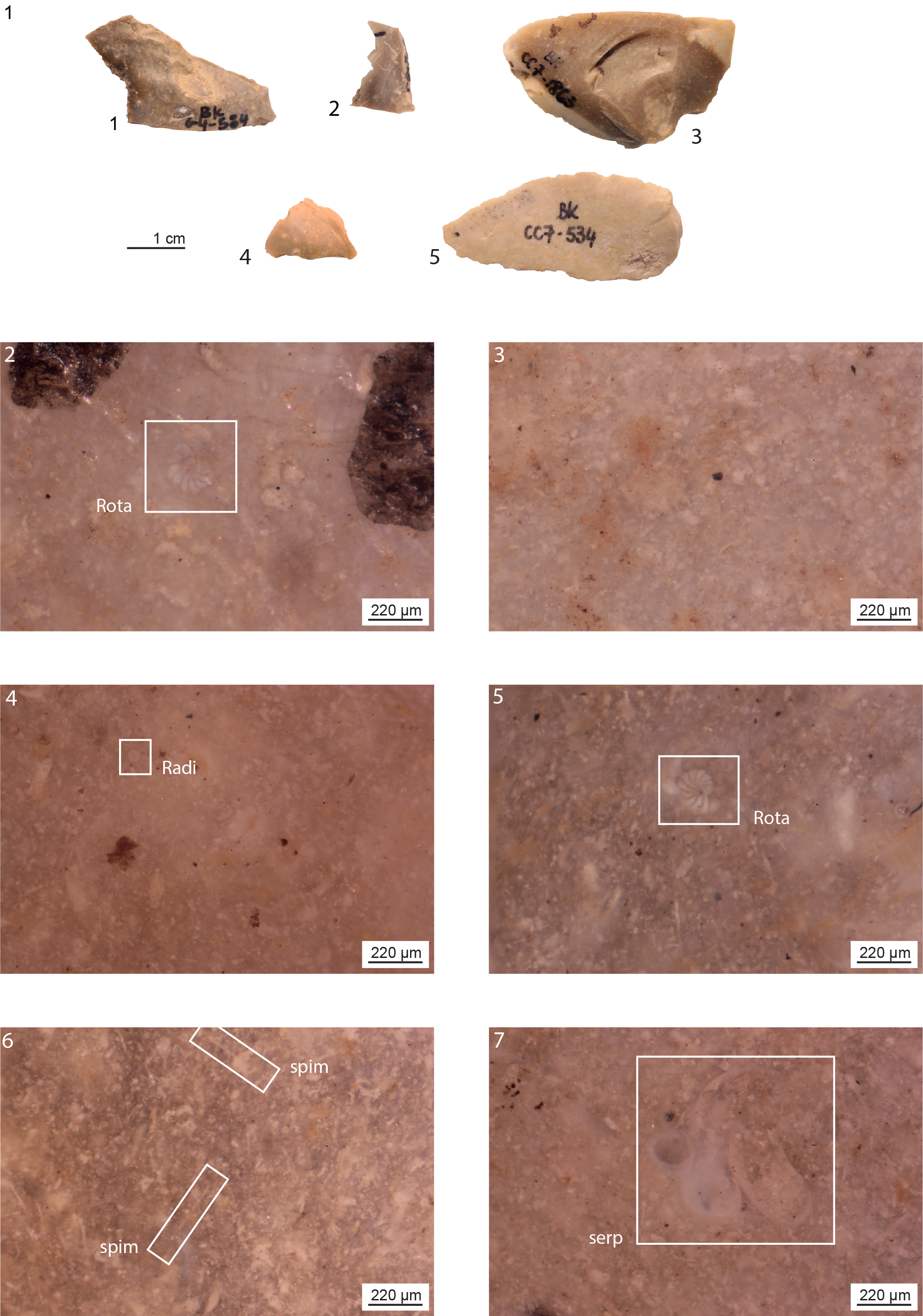

Supplement: S4 Fig — 1. Macroscopic view; 2 à 7. Mesoscopic view. Spim: monaxon spicule of Hexactinnelide sponges; Rota: benthic foraminifera cf. Rotalidea; radi: spumellar radiolarian; serp: worms tubes cf. serpulidae. (TIF) [file pone.0307435.s004.tif]

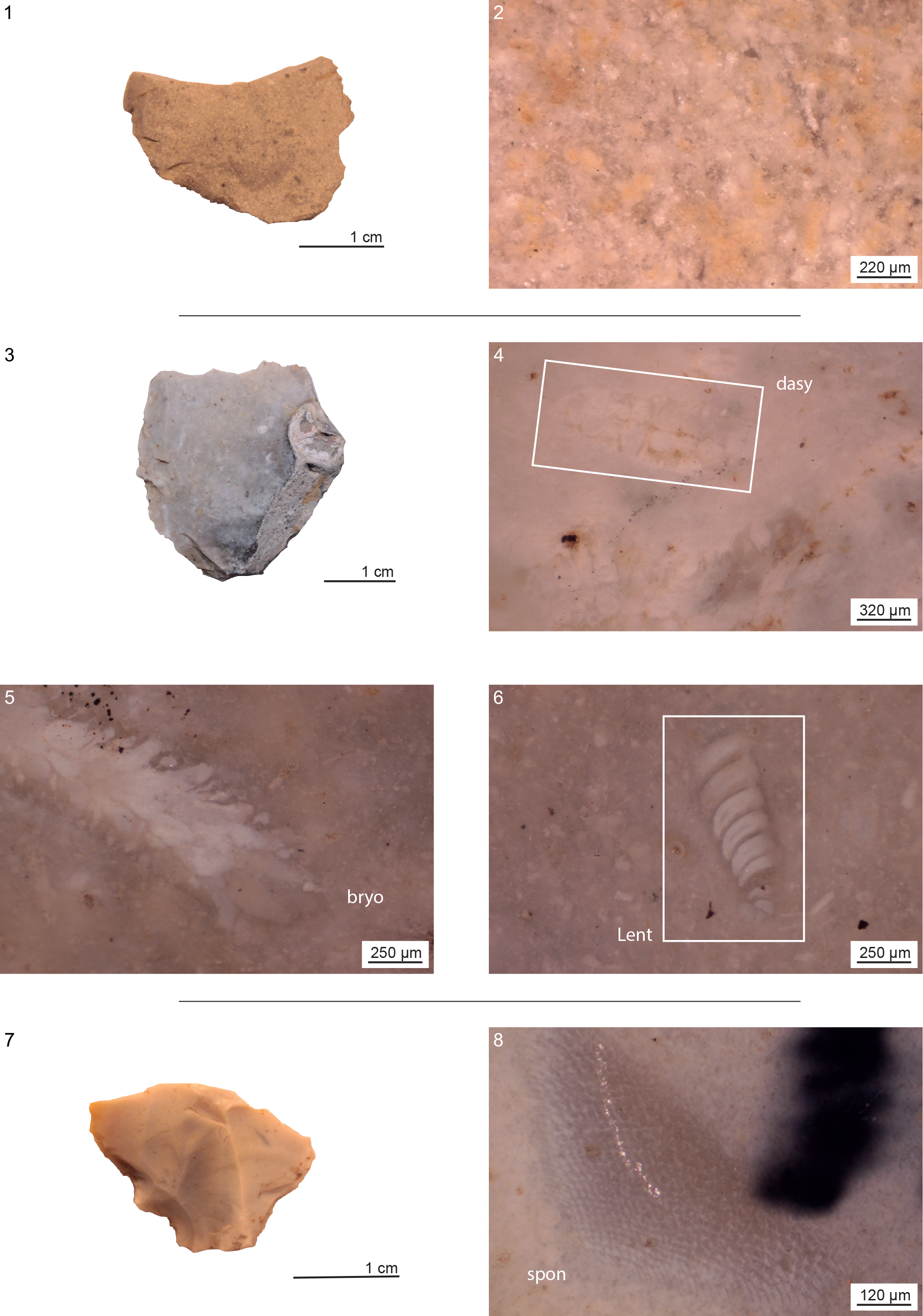

Supplement: S5 Fig — 1. Macroscopic view; 2. Mesoscopic view. Groupe 35: 3. Macroscopic view; 4 à 6. Mesoscopic view. Groupe 36: 7. Macroscopic view; 8. Mesoscopic view. Dasy: Marine green algae cf. dasycladale; bryo: cyclostome bryozoans; Lent: benthic foraminifera cf. Lenticulina; spon: fused spicules of sponges. (TIF) [file pone.0307435.s005.tif]

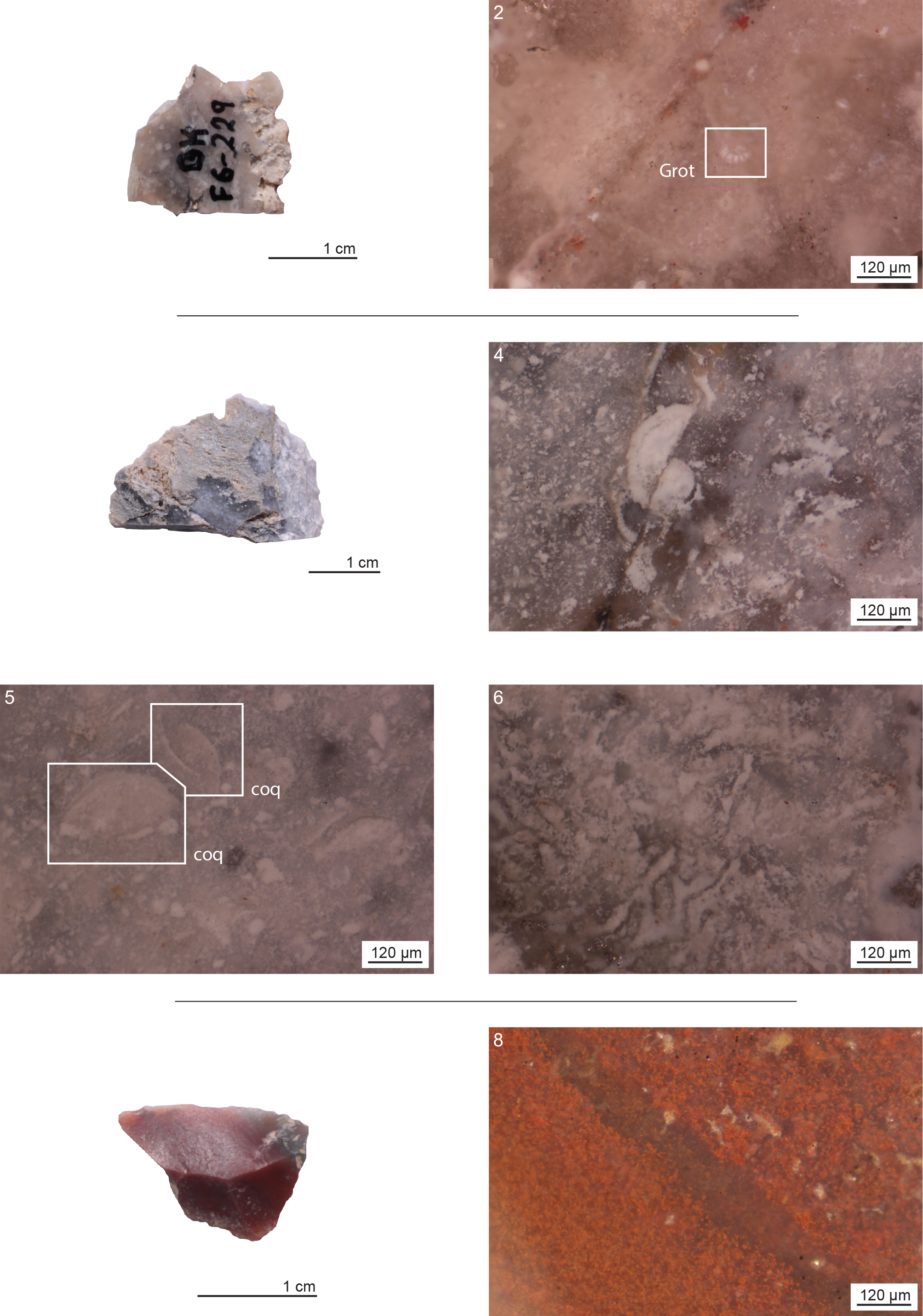

Supplement: S6 Fig — 1. Macroscopic view; 2. Mesoscopic view. Groupe 42: 3. Macroscopic view; 4 à 6. Mesoscopic view. Groupe 51: 7. Macroscopic view; 8. Mesoscopic view. Glob: planktonic foraminifera cf. Globorotalidea; coq: fragment of undetermined shell. (TIF) [file pone.0307435.s006.tif]

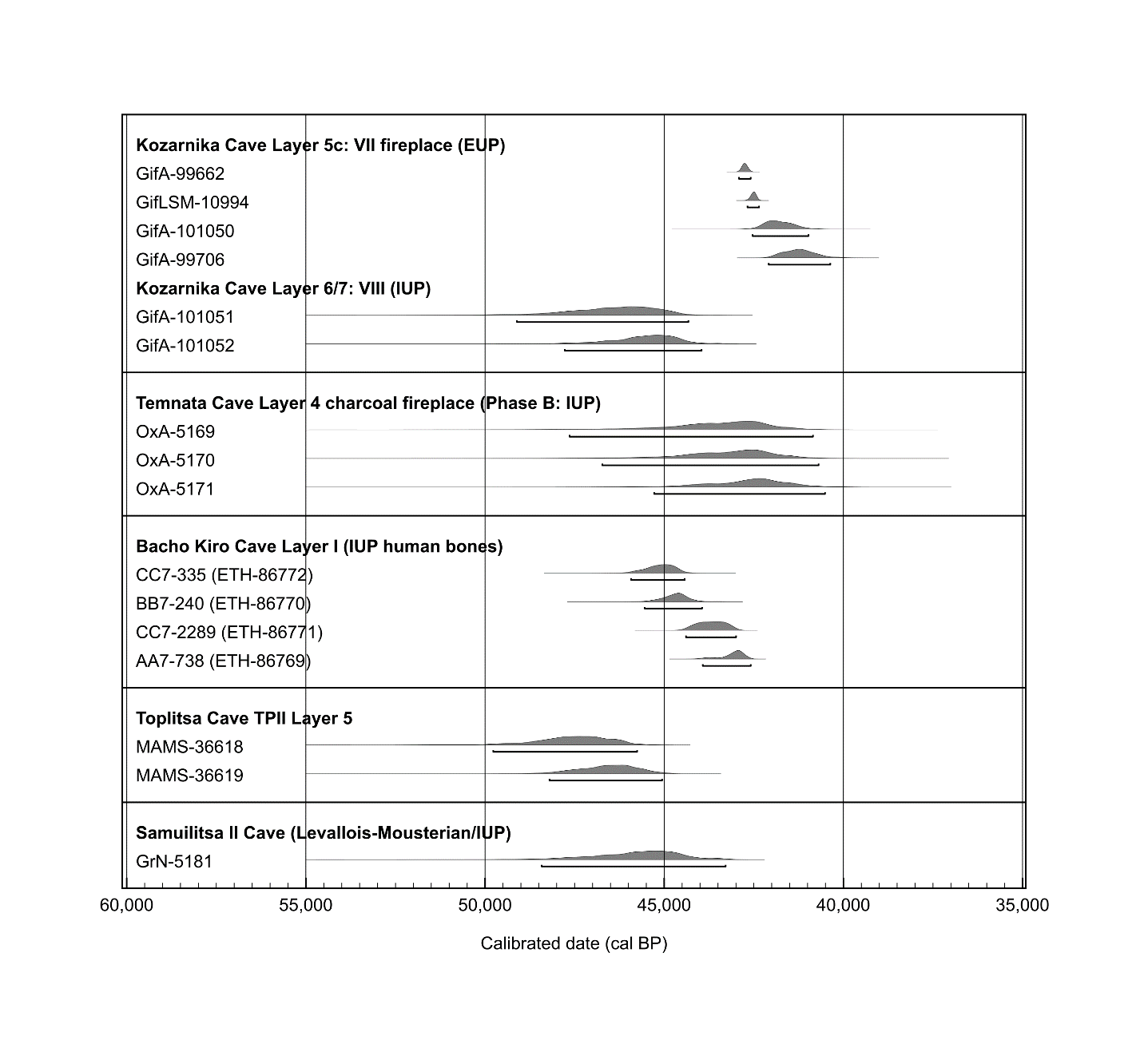

Supplement: S11 Fig — Plotted calibrated radiocarbon dates from Kozarnika, Temnata, Bacho Kiro and Samuilitsa II caves. The original dates from Kozarnika and Temnata caves are on charcoal, while those from Bacho Kiro Cave are on bone. The single date from Samuilitsa II Cave, made on bone, is 42,780 ± 1270 uncalBP (GrN- 5181). All dates are recalibrated, using Oxcal v.4.4 with IntCal20 curve [119]. (DOCX) [file pone.0307435.s011.docx]

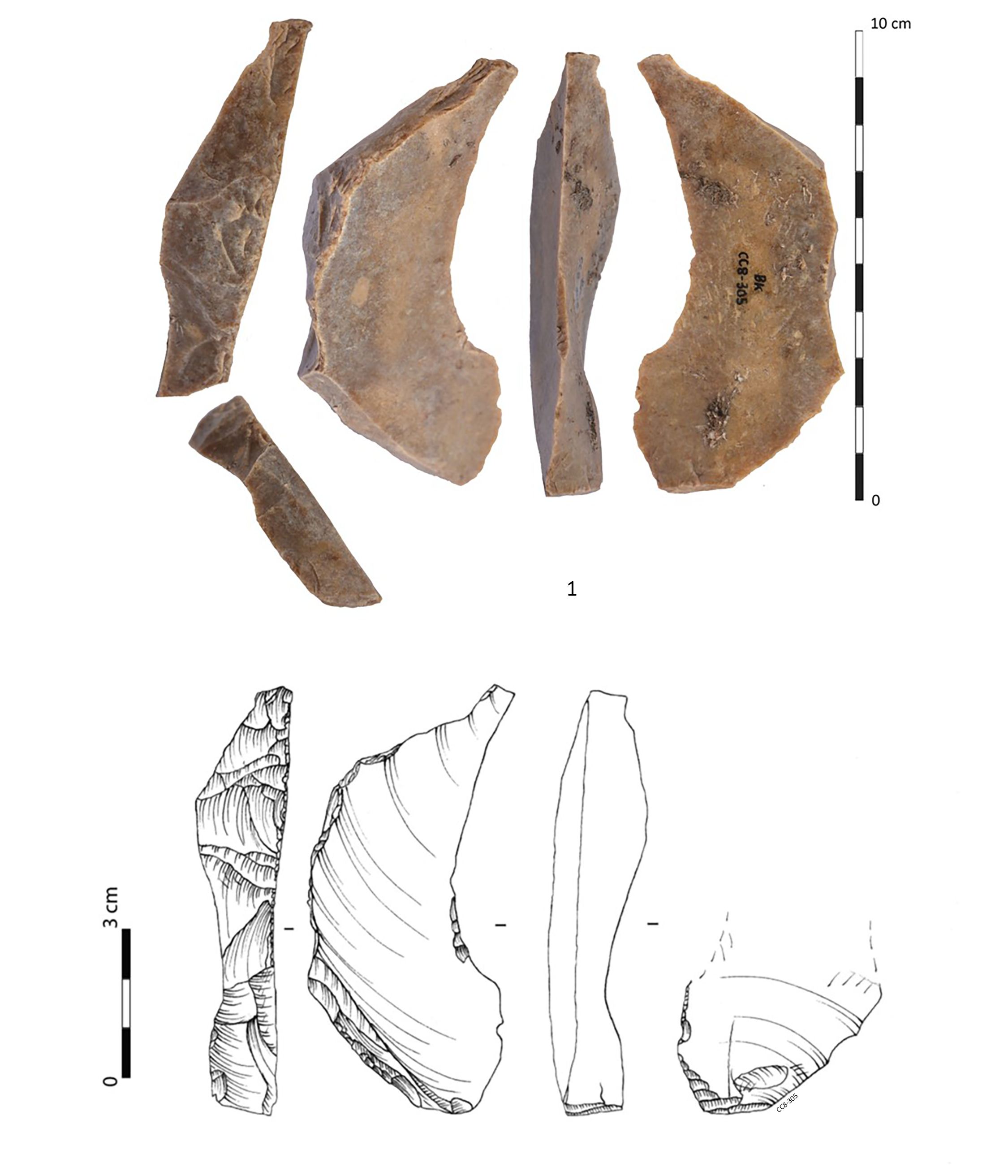

Supplement: S12 Fig — (TIF) [file pone.0307435.s012.tif]
