# Supplementary material for: Curated character of the Initial Upper Palaeolithic lithic artefact assemblages in Bacho Kiro Cave (Bulgaria)
Source: PLoS One. 2024 Sep 4;19(9):e0307435. doi: 10.1371/journal.pone.0307435 (PMC11373871; doi:10.1371/journal.pone.0307435)
Supplement: S7 Fig — Scatter-plot of cores dimensions: length and width (left) and width and thickness (right) of freehand and bipolar cores, IUP layers, Bacho Kiro Cave. (DOCX) [file pone.0307435.s007.docx]

| 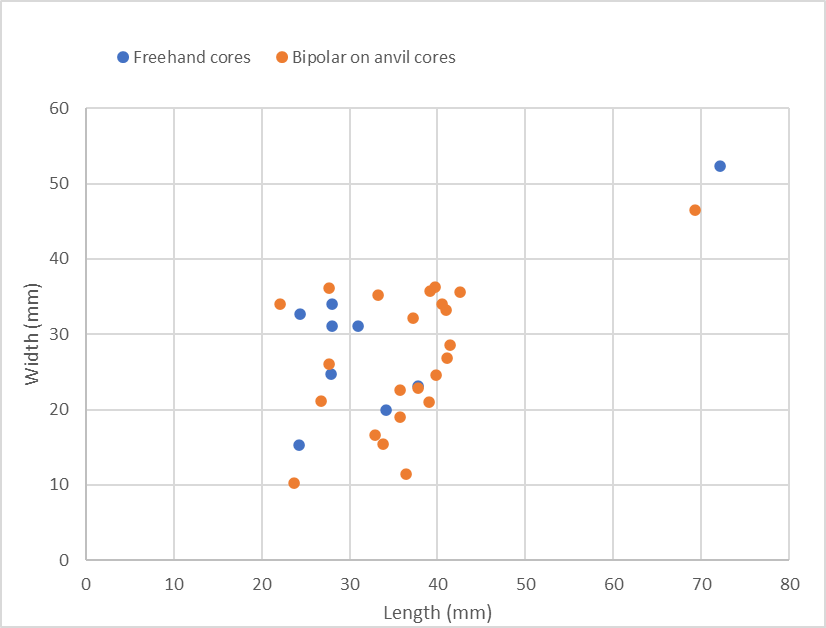 | 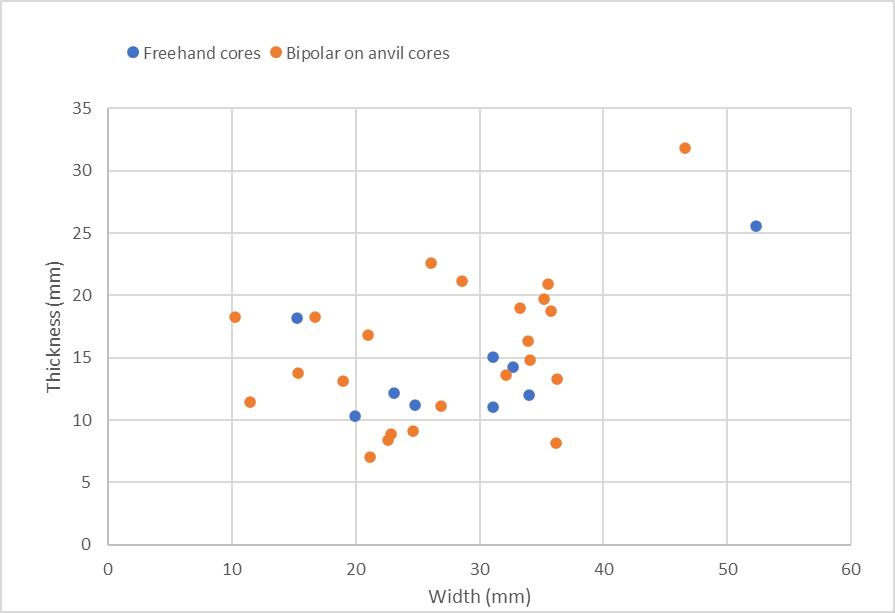 |
| --- | --- |

**S7 Figure. Scatter-plot of cores dimensions**: length and width (left) and width and thickness (right) of freehand and bipolar cores, IUP layers, Bacho Kiro Cave.
