# Supplementary material for: Curated character of the Initial Upper Palaeolithic lithic artefact assemblages in Bacho Kiro Cave (Bulgaria)
Source: PLoS One. 2024 Sep 4;19(9):e0307435. doi: 10.1371/journal.pone.0307435 (PMC11373871; doi:10.1371/journal.pone.0307435)
Supplement: S9 Fig — Clustered column and bar comparing the blade profiles types of unretouched blades and blade tools (left) and blade cross-section types (right), IUP layers, Bacho Kiro Cave. (DOCX) [file pone.0307435.s009.docx]

|  |  |
| --- | --- |
