# Supplementary material for: Curated character of the Initial Upper Palaeolithic lithic artefact assemblages in Bacho Kiro Cave (Bulgaria)
Source: PLoS One. 2024 Sep 4;19(9):e0307435. doi: 10.1371/journal.pone.0307435 (PMC11373871; doi:10.1371/journal.pone.0307435)
Supplement: S10 Fig — (DOCX) [file pone.0307435.s010.docx]

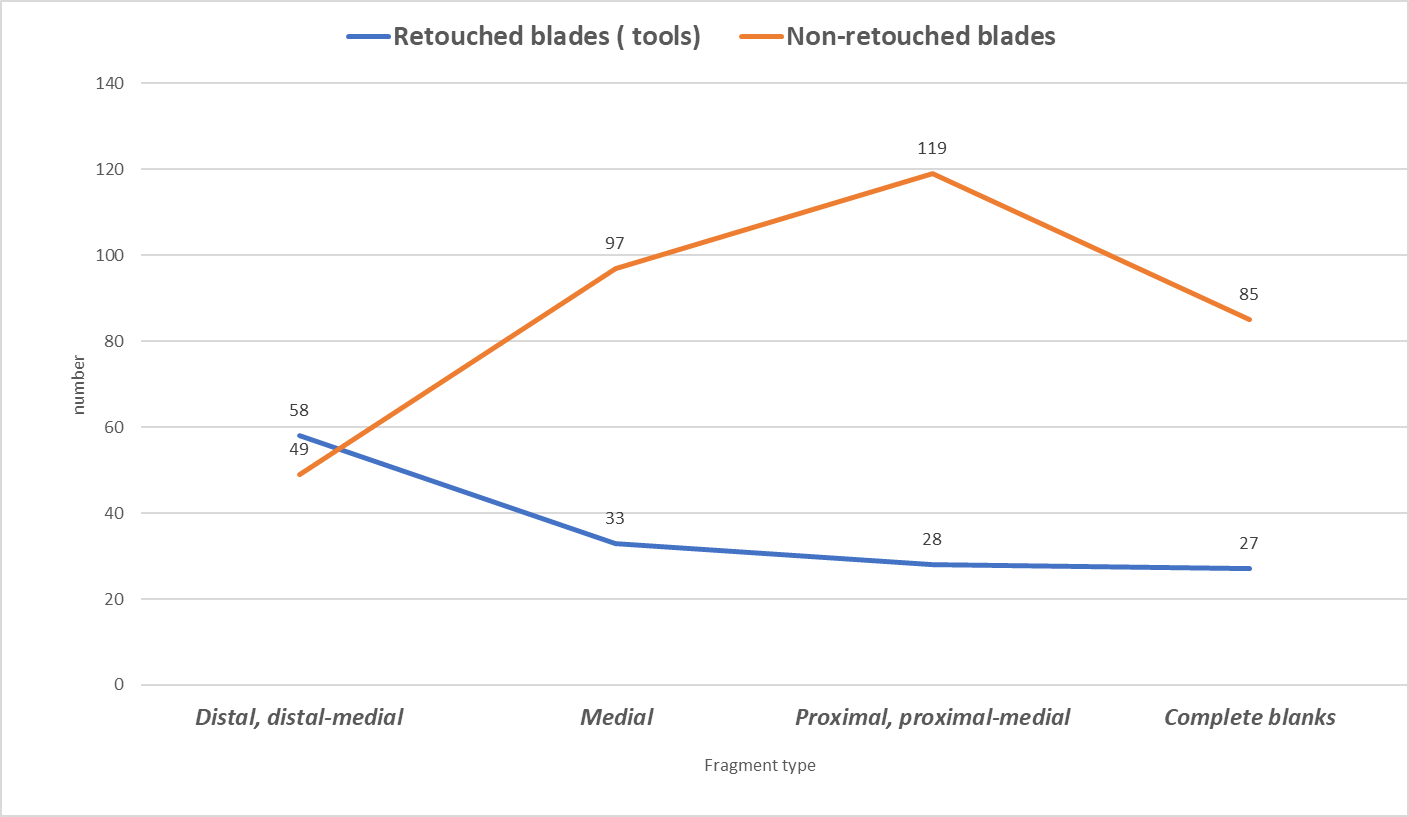


**S10 Figure. Comparison of the distribution of blade fragments** (distal, medial, and proximal) for retouched and non-retouched blades in relation to complete blades.
