# Supplementary material for: Curated character of the Initial Upper Palaeolithic lithic artefact assemblages in Bacho Kiro Cave (Bulgaria)
Source: PLoS One. 2024 Sep 4;19(9):e0307435. doi: 10.1371/journal.pone.0307435 (PMC11373871; doi:10.1371/journal.pone.0307435)
Supplement: S1 Table — (DOCX) [file pone.0307435.s013.docx]

| ***Main Sector (MS)***  **Layers** | **Lithic** | **Bone** | **Nb of buckets** |
| --- | --- | --- | --- |
| **3c/3d** | 0 | 12 | 10 |
| **A0** | 2 | 7 | 27 |
| **A1** | 6 | 31 | 142 |
| **A1/A2** | 0 | 1 | 0 |
| **A2** | 11 | 87 | 85 |
| **A2a/b** | 2 | 4 | 0 |
| **B** | 108 | 566 | 125 |
| **B/C** | 2 | 20 | 2 |
| **C** | 20 | 190 | 60 |
| **D** | 0 | 40 | 25 |
| **E** | 0 | 81 | 39 |
| **F** | 0 | 87 | 65 |
| **G1** | 0 | 4 | 52 |
| **G2** | 1 | 9 | 34 |
| **G3** | 2 | 2 | 13 |
| **G3/H** | 0 | 0 | 1 |
| **H** | 1 | 0 | 3 |
| **H/I** | 12 | 7 | 5 |
| **I** | 43 | 135 | 4 |
| **I/J** | 26 | 130 | 6 |
| **J** | 10 | 139 | 15 |
| **Total Main Sector** | 246 | 1552 | 713 |
| **No context (back dirt)** |  | 15 | 2 |
| ***Niche 1 (N1) Sector*** | **Lithic** | **Bone** | **Nb of buckets** |
| **N1 3c/3d** | 0 | 5 | 4 |
| **N1 3d/3e** | 0 | 8 | 8 |
| **N1 G1/G2** | 0 | 0 | 6 |
| **N1-1** | 0 | 17 | 17 |
| **N1-2** | 1 | 18 | 14 |
| **N1-3a** | 0 | 50 | 14 |
| **N1-3b** | 0 | 37 | 32 |
| **N1-3b** | 0 | 1 | 0 |
| **N1-3b/3c** | 0 | 7 | 10 |
| **N1-3c** | 1 | 28 | 13 |
| **N1-3d** | 0 | 12 | 16 |
| **N1-3e** | 0 | 52 | 34 |
| **N1-3e/G1** | 0 | 3 | 4 |
| **N1-G1** | 0 | 13 | 92 |
| **N1-G2** | 0 | 9 | 114 |
| **N1-G2/H** | 0 | 0 | 2 |
| **N1-G3** | 0 | 1 | 13 |
| **N1-H** | 93 | 226 | 88 |
| **N1-H/I** | 251 | 662 | 26 |
| **N1-I** | 1808 | 12578 | 97 |
| **N1-I/J** | 127 | 1437 | 37 |
| **N1-J** | 53 | 1903 | 207 |
| **N1-J/K** | 8 | 279 | 67 |
| **N1-K** | 93 | 890 | 170 |
| **Total Niche 1** | 2435 | 18236 | 1085 |
| **Total (both sectors)** | **2681** | **19803** | **1800** |

**S1 Table. Bacho Kiro Cave, IUP layers. Counts of piece-plotted lithic (>1.5 cm), fauna remains (>2 cm), and collected and sieved sediments (1 bucket contains 9 liters of sediment).**
