# Supplementary material for: Curated character of the Initial Upper Palaeolithic lithic artefact assemblages in Bacho Kiro Cave (Bulgaria)
Source: PLoS One. 2024 Sep 4;19(9):e0307435. doi: 10.1371/journal.pone.0307435 (PMC11373871; doi:10.1371/journal.pone.0307435)
Supplement: S4 Table — (DOCX) [file pone.0307435.s016.docx]

| **Core blank/Technique** | **Bipolar** | **Freehand** | **Total** | **%** |
| --- | --- | --- | --- | --- |
| **Concretion** |  | 1 | 1 | 3.1 |
| **Pebble** | 3 |  | 3 | 9.4 |
| **Flake** | 5 | 3 | 8 | 25 |
| **Blade** | 1 |  | 1 | 3.1 |
| **Blade or flake** | 8 |  | 8 | 25 |
| **Core** | 2 |  | 2 | 6.3 |
| **Tool fragment** | 1 |  | 1 | 3.1 |
| **Undeterminable** | 4 | 4 | 8 | 25 |
| **Total** | 24 | 8 | 32 | 100 |
| **%** | 75 | 25 | 100 |  |

**S4 Table. Core blanks for the bipolar and freehand cores in the IUP layers from Bacho Kiro Cave.**
