# Supplementary material for: Curated character of the Initial Upper Palaeolithic lithic artefact assemblages in Bacho Kiro Cave (Bulgaria)
Source: PLoS One. 2024 Sep 4;19(9):e0307435. doi: 10.1371/journal.pone.0307435 (PMC11373871; doi:10.1371/journal.pone.0307435)
Supplement: S5 Table — (DOCX) [file pone.0307435.s017.docx]

| Platform type | Bacho Kiro Cave,  IUP Layers I, J | % | Temnata-I, Layer 4 | % |
| --- | --- | --- | --- | --- |
| Cortical, natural | 2 | 0.77 | 9 | 1.04 |
| Plain | 134 | 51.74 | 483 | 56.23 |
| Dihedral | 2 | 0.77 | 41 | 4.78 |
| Facetted | 47 | 18.15 | 202 | 23.51 |
| Linear | 27 | 10.42 | 14 | 1.63 |
| Punctiform | 4 | 1.54 | 6 | 0.7 |
| Shattered | 1 | 0.39 |  | 0 |
| Smashed | 10 | 3.87 | 4 | 0.46 |
| Broken | 28 | 10.81 |  | 0 |
| Undeterminable | 4 | 1.54 | 100 | 11.65 |
| Total: | 259 | 100 | 859 | 100 |

**S5 Table. Comparison of blade platform types between the IUP assemblages from Bacho Kiro (Layers I and J), and Temnata caves (layer 4).**
