# Supplementary material for: Curated character of the Initial Upper Palaeolithic lithic artefact assemblages in Bacho Kiro Cave (Bulgaria)
Source: PLoS One. 2024 Sep 4;19(9):e0307435. doi: 10.1371/journal.pone.0307435 (PMC11373871; doi:10.1371/journal.pone.0307435)
Supplement: S6 Table — (DOCX) [file pone.0307435.s018.docx]

| **Fragment type** | **Blade tools** | **%** | **Flake tools** | **%** | **Total** | **%** |
| --- | --- | --- | --- | --- | --- | --- |
| **Distal** | 31 | 25.83 | 17 | 20.73 | 48 | 23.76 |
| **Mesial-distal** | 27 | 22.5 | 16 | 19.51 | 43 | 21.29 |
| **Mesial** | 33 | 27.5 | 23 | 28.05 | 56 | 27.72 |
| **Proximal-mesial** | 22 | 18.34 | 6 | 7.32 | 28 | 13.87 |
| **Proximal** | 6 | 5 | 11 | 13.41 | 17 | 8.41 |
| **Lateral left part** |  | 0 | 2 | 2.44 | 2 | 0.99 |
| **Lateral right part** | 1 | 0.83 | 2 | 2.44 | 3 | 1.49 |
| **Undeterminable** |  | 0 | 5 | 6.1 | 5 | 2.47 |
| **Total fragments** | **120** | 100 | **82** | 100 | **202** | 100 |
| **Fragments %** |  | 59.4* |  | 40.6* | 100 |  |
| **Complete tool blanks** | 27 | 18.36** | 67 | 44.97** | **94** | 31.76*** |
| **Total (fragments+** **completes)** | **147** | 49.66*** | **149** | 50.34*** | **296** |  |

***Total number of fractures for the retouched tools is 248, fragmentation rate is 68.2%***

* related to the total number of fragmented blade and flake tools (n=202), ** related to the total number of the group (blade tools n=147, flake tools n=149), *** related to the total number of complete and fragmented artifacts (n=296)

**S6 Table. Distribution of retouched tool fragments in the IUP layers from Bacho Kiro Cave.**
