# Supplementary material for: Curated character of the Initial Upper Palaeolithic lithic artefact assemblages in Bacho Kiro Cave (Bulgaria)
Source: PLoS One. 2024 Sep 4;19(9):e0307435. doi: 10.1371/journal.pone.0307435 (PMC11373871; doi:10.1371/journal.pone.0307435)
Supplement: S7 Table — (DOCX) [file pone.0307435.s019.docx]

| **Fragments types** | **Fragmented blanks and tools** |  |  |  | **Fragmented blanks, tools with bulb on one of the breaks** |  |  |  |  |  |  |  |  |
| --- | --- | --- | --- | --- | --- | --- | --- | --- | --- | --- | --- | --- | --- |
|  | **NR* Blade** | **NR Flake** | **Blade tools** | **Flake tools** | **NR Blade** | **NR Blade or Flake** | **NR Flake** | **Bipolar core** | **Scaled piece** | **Blade tool** | **Blade or Flake tool** | **Flake tool** | **Tool fragment** |
| **Distal** | 14 | 46 | 31 | 17 |  |  | 2 |  |  | 1 | 2 | 3 |  |
| **Mesial-distal** | 35 | 50 | 27 | 16 |  |  |  |  |  |  |  |  | 1 |
| **Mesial** | 97 | 70 | 33 | 23 | 1 | 2 | 2 |  | 3 | 2 | 3 |  | 6 |
| **Proximal-mesial** | 83 | 74 | 22 | 6 | 3 |  | 5 |  | 1 |  |  |  |  |
| **Proximal** | 36 | 74 | 6 | 11 | 2 |  | 3 | 1 |  | 1 |  |  |  |
| **Lateral left part** |  | 4 |  | 2 |  |  |  |  |  |  |  |  |  |
| **Lateral right part** |  | 3 | 1 | 2 |  |  |  |  |  |  |  |  |  |
| **Undeterminable** | 3 | 48 |  | 5 |  | 1 |  |  |  |  |  |  | 3 |
| **Total** | **268** | **369** | **120** | **82** | **6** | **3** | **12** | **1** | **4** | **4** | **5** | **3** | **10** |

* NR=Non retouched

**S6 Table. Counts of the artefact fragments with percussion marks and bulbs on the break attesting a deliberate fragmentation in the IUP layers from Bacho Kiro Cave.**
