# Supplementary material for: Curated character of the Initial Upper Palaeolithic lithic artefact assemblages in Bacho Kiro Cave (Bulgaria)
Source: PLoS One. 2024 Sep 4;19(9):e0307435. doi: 10.1371/journal.pone.0307435 (PMC11373871; doi:10.1371/journal.pone.0307435)
Supplement: S8 Table — (DOCX) [file pone.0307435.s020.docx]

|  | **Initial Upper Palaeolithic** | | | **EUP before CI Y-5 eruption >39 Ka BP** |
| --- | --- | --- | --- | --- |
| **Site** | **Bacho Kiro, layer I** | **Temnata-II, layer VI** | **Temnata-I, layer 4** | **Kozarnika, layer VII** |
| **Raw material** | Non-local (imported) fine-grained Aptian and Campanian flint | Local, coarse-grained, grey flint: 76.5%;  Fine-grained non-local flint: 22.1%. | Local, coarse-grained grey flint: 77.7%;  Fine-grained flint: 19.4% | Local, fine-grained grey flint: 86.9%;  Non-local, fine-grained yellow flint: 6.99%. |
| **Raw material blanks** | Unknown, probably nodules > 12 cm | Nodules local flint >10 cm and gelifracted slabs | Nodules local flint >10 cm and gelifracted slabs | Nodules local flint 4–8 cm; gelifracted slabs |
| **Main production** | Imported blades, On site production of small blades, and flakes | Elongated flakes and blades to small blades | Blades to small blades | Small blades to bladelets |
| **Methods** | Bidirectional pyramidal cores with shifted platforms; Unidirectional likely volumetric, on a broad and narrow flaking surface | Levallois, Prismatic, Bidirectional blade cores, few unidirectional blade cores,  Circular and semi-circular debitage progression | Prismatic, Bidirectional and unidirectional blade cores  Circular and semi-circular debitage progression | Pyramidal unidirectional bladelet cores and prismatic bidirectional bladelet cores;  Semi-circular debitage progression |
| **Reduction for small laminar blanks (*redébitage*)** | Splintered pieces, Kombewa type, Kostienki type, Burin-cores | Burins-cores | Kombewa type, burins-cores, splintered pieces | Burin cores: debitage on the long edge of the blank |
| **Knapping techniques** | Direct hard hammer,  On anvil percussion (Bipolar) | Direct hard hammer | Direct hard hammer,  On anvil percussion (Bipolar) | Direct soft hammer,  Direct hard hammer |
| **Blade Economy: Retouched blades/bladelet** | Pointed and retouched blades, endscrapers, sidescraper… | Pointed and retouched blades, endscrapers, sidescrapers… | Pointed and retouched blades endscrapers… | Pointed bladelets, retouched blades and bladelets, endscrapers, burins… |

**S8 Table. Comparison with regional IUP blades and EUP bladelets assemblages. Techno-economic features.**
