# Supplementary material for: Curated character of the Initial Upper Palaeolithic lithic artefact assemblages in Bacho Kiro Cave (Bulgaria)
Source: PLoS One. 2024 Sep 4;19(9):e0307435. doi: 10.1371/journal.pone.0307435 (PMC11373871; doi:10.1371/journal.pone.0307435)
Supplement: S1 File — (DOCX) [file pone.0307435.s021.docx]

**S1Appendix.** **Radiocarbon dates and models with Radiocarbon 3.0.** A new study of the radiocarbon dates from the last excavation of Layer I in Bacho Kiro Cave suggests that there are 2 or 3 distinct occupations (1) . The newest 14 C recalibration of the directly dated *Homo sapiens* by radiocarbon 3.0 and the floating tree-ring Kauri chronology of OxCal indicates that in the case of 2 phases model IUP spans between 44,630 to 43,860 cal BP (68,3%) for a total of 770 year (1) while in the 3 phases model the range is between 44,490 to 42,780 cal BP for total of 1,710 year (1).

**S2 Appendix.** **Terminology petrography.** From a petrological perspective, we mean by “genetic type” a set of rocks that share similar characteristics of age, formation environment and type of diagenesis. These characteristics – which relate to the genesis of the rock – are defined by a certain number of chemical, biological and detrital criteria (2-4).

**S3 Appendix.** **Definition of term silicite.** Extending the definition of (4), the term silicite here designates all rocks having undergone silicification of chemical, biochemical or diagenetic origin (flint, chert, silcrete and hydrothermal silica). It allows us to free ourselves from the term “flint”, the meaning of which is subject to controversy and misunderstanding depending on whether it is used by geologists, petrographers, sedimentologists or archaeologists. We therefore use the term flint only to designate rocks having undergone initial diagenesis followed by siliceous epigenesis and bearing a cortex.

**S4 Appendix**. **Methodology for raw material studies.** The analysis of silicites necessitates the utilization of dynamic models to decipher the various stages of diagenesis and the history of post-genetic processes e.g.(5). These factors must be considered during geological sampling or when examining archaeological objects and remain of paramount importance throughout the preparation and analysis of samples. Our approach entails a series of determinations and systematic measurements at multiple scales of the silicite's surface, the figured elements, the type of subcortical band, porosity, and poronecrosis (6).

The initial phase of this approach commences in the field, as constructing a lithotheca involves more than random collection during surveys; it entails identifying and tracing the occurrences of the same genetic type in every deposit, which serves as evidence of its origin. The quality of this sampling significantly influences subsequent petroarchaeological interpretations and the reliability of sourcing. To achieve this, we've developed a standardized method for cataloguing silicite formations. This method is applicable both during field surveys and when organizing existing lithotheca. Each deposit in the inventory record comprises 52 descriptive fields, covering metadata (authors, date, project type, lithothèque, etc.) and data (deposit location, geological information, deposit type, collection type, etc.) (7). In this case, we utilized the lithotheca ofthe *Earth and Man Museum* in Sofia as a referential. This lithotheca encompasses over 250 deposits from primary formations, clay with flint, and alluvial deposits across Bulgaria, excluding the ancient terraces of the Danube Valley, which have not been sampled.

To structure the microfacies observations and characterize the material, we have developed three analysis grids, grouped together in a database (3, 6, 8). Each of these grids focuses on a particular moment in the silicites' journey. Petroarchaeological analysis commences with the examination of the geological reference samples, which is then applied to archaeological materials to establish groups. The description of geological samples allows for the definition of types, understood as a population of geological samples of known origin (cf. source), defined by specific (bio)stratigraphic, sedimentological, diagenetic, and alteration criteria. These types serve as a reference and enable, through comparison, the repositioning of material groups derived from archaeological analysis – conducted using the same techniques (see below) as those on geological material – in space. In this regard, the groups constitute an analytical construction by the petro-archaeologist, gathering the facies presented by archaeological objects with similar characteristics in terms of (bio)stratigraphy, formation environment, siliceous diagenesis, and post-genetic evolution. Thus, the comparison of these criteria with those previously defined on geological material, judged diagnostic in establishing the reference framework, allows for a correlation between samples. In the present case, material groups are named with randomly assigned numbers, without any hierarchy within this nomenclature. Each material group therefore possesses unique characteristics described in the summary **S2 Table**. The variety of microfacies for each group is illustrated in the **S1 to S6 Figures**.

Grid 1, "petrography," comprises 72 fields designed to identify the genetic type. It enables the characterization of microfacies and the chemical, biological, and detrital components for each zone of every sample. The matrix and figured elements are described through the observation of all sample types at high magnification under a binocular loupe (minimum magnification x 80), initially dry and then under a water film. This microfacies description process helps determine the environment of formation and the age of the silicified rock, primarily based on the figured elements (lithoclastic, detrital, chemical, and biogenic, following (9), each assessed in terms of nature, abundance, orientation, sorting, bluntness, sphericity, and size. While specific attention is given to the biogenic fraction of clasts, other elements, especially the detrital fraction (often overlooked in favor of microfossils), serve as valuable proxies to distinguish variations in genetic types e.g., (10, 11). This analytical grid employs the vocabulary, charts, and diagrams established by sedimentologists and petrographers over the past sixty years (2, 12-17). All figured elements and their relationships with the matrix are thus considered. Understanding the nature and stratigraphic origin of a silicite type (marine, evaporitic, or lacustrine flint, pedogenetic or groundwater silcrete, hydrothermal silica, etc.) and its formation environment is crucial. It allows verification of whether differences in alterations stem from initial variety or, conversely, helps clearly identify the genetic variability of a type, which itself partly accounts for geological variability. In the present case, we have identified within the Bacho Kiro collection 19 silicite groups (see **S3 Table**). Seven originate from formations of the Lower Cretaceous in northeastern Bulgaria, ten come from various formations of the Upper Cretaceous in the east or northwest of Bulgaria, and a single object exhibits the characteristic criteria of a volcanogenic silica from the Rhodope Mountains (in a broad sense, as sampling of such formations in southern Bulgaria remains very limited).

At this stage of the analysis, it is necessary to decipher the types of mechanical actions, such as cracking or fragmentation, and the chemical processes, such as alterations or the diffusion of oxides in the matrix. This is the objective of grid 2 "Weathering." Organizing stigmata and traces provides the key to understanding the sequence of predepositional transformations, with each association being characteristic of a type of geological context (18-21). The history of the silicite's surface 'life' accompanies internal transformations of the material, and in extreme cases, evolutions can erase any relationship with the initial material (3). As the silicite matrix evolves, its chemical and mineralogical composition changes: disappearance of carbonates in the broad sense, replacement of siliceous phases in favor of microquartz, filling and/or secondary epigenesis of figured elements and/or pores, appearance of neo-porosity at the periphery of figured elements in the form of whitish halos, increase in the proportion of iron oxides (and other chemical elements), and/or filling of figured elements. Cross-referencing these different weathering data helps discriminate the evolved facies of a genetic type as defined in grid 1, and if the measurement of the degree of transformation of an archaeological object is equivalent to a reference evolved geological sample, it is possible to link it to a type of geological formation. Each genetic type is thus divided into as many gitological types, with the degree of alteration potentially correlated with the distance from the initial source (for the Bacho Kiro groups, see **S3 Table**); the greater the distance, the more the rock block tends to take on the form of a pebble and homogenize.

Finally, according to the concept of the evolutionary chain, silicites tend toward chemical equilibrium with the environment of their last place of residence without erasing the characteristics inherited from previous places of residence. In the case of a knapped object, it is necessary not only to determine the genetic (grid 1) and geological (grid 2) origin of the material but also to consider its post-depositional evolution occurring during its presence at its last place of residence, namely the archaeological site. A third scale of analysis is thus added to the previous ones, following the steps of the "Postdepositional processes" grid. In addition to provide information on the implementation and possible deformations of the archaeological level, the degree of weathering of the removals serves as a key indicator to understand the succession of modifying processes, before or after the abandonment of the object into the site. By virtually subtracting weathering marks associated with the post-depositional phase from non-anthropic surfaces, we can specify the type of formation in which the silicite was collected. Deciphering the chronology of impacts on the (neo)cortex is the only way to strictly prioritize associations of stigmata and to relate them to a pre-depositional or post-depositional stage.

**S5 Appendix. Methods of lithic analysis.** The lithic artifacts are orientated and analyzed according to the techno-typological and lithic economic approaches described in (22). For the recognition of the knapping techniques, our classification refers to (23), combined with platform type and exterior platform angles of blanks which enable the interpretation of the hammer type (hard stone, soft stone, or soft organic). Attention is given to the availability and initial shape of the lithic raw material, concepts and schemas of flake, blade and bladelet productions and objectives (final products) of the *chaîne opératoire*. We reconstructed blade technology through a technical ‘“reading’” of blades and their recording: cortex amount, cresting, dorsal scar patterns (the directions of the scars left from previously removed blanks), platform type and exterior platform angle, according to (24): p.136); curvature is qualified according to (25, 26); blade shape is measured according to the delineation of the lateral edges and transversal sections; blade dimensions (length, width, and thickness). For tracking tendencies in laminar blank selection, we examined the mean values in those dimensions of retouched versus non-retouched blades/bladelets.

We followed the metric criteria for a category of “bladelets” as proposed by (27): length/width ratios of equal or greater than 2:1, width between 7 and 12 mm, and a maximum length of 50 mm, and microblades with a width less than 7 mm. In the case of the examined laminar collection the use of term “bladelet” does not correspond technologically to the original UP definition of bladelet (op. cit.), as the so-called bladelets in the present IUP assemblages correspond only by metric criteria. However, here we do not adhere to the production criteria (produced from prismatic cores by organic soft hammer percussion) for the bladelet category as per (28) the bladelets in the IUP from Bacho Kiro were produced mostly by on anvil percussion technique. In this paper the splintered pieces are classified in term of *chaîne opératoire* approach and transformation of blanks and tools into bipolar cores and scaled pieces (29, 30).

For the analysis of blank platforms, all preserved platforms are categorized into two groups: blades (comprising complete unretouched blades and proximal fragments) and flakes (consisting of complete unretouched flakes, flake tools, and proximal fragments). For the dorsal scar pattern analysis, are considered the complete and fragmented blades and flakes, because of the low number of complete products.

The lithic artifacts are orientated and analyzed according to the techno-typological and lithic economic approaches described in (22). For the recognition of the knapping techniques, our classification refers to (18), combined with platform type and exterior platform angles of blanks which enable the interpretation of the hammer type (hard stone, soft stone, or soft organic). Attention is given to the availability and initial shape of the lithic raw material, concepts and schemas of flake, blade and bladelet productions and objectives (final products) of the *chaîne opératoire*. We reconstructed blade technology through a technical ‘“reading’” of blades and their recording: cortex amount, cresting, dorsal scar patterns (the directions of the scars left from previously removed blanks), platform type and exterior platform angle, according to (24): p.136); curvature is qualified according to (25, 26); blade shape is measured according to the delineation of the lateral edges and transversal sections; blade dimensions (length, width, and thickness). For tracking tendencies in laminar blank selection, we examined the mean values in those dimensions of retouched versus non-retouched blades/bladelets.

Retouched tools are classified by typological categories following for the UP and (31) for the MP. Additionally we referred to the translated UP tools typology of (32). Special attention is given to the pointed ‘diagnostic’ tools presumably some of them connected to subsistence hunting activities.

**S6 Appendix.** **Supplement regional comparisons.** In the site of Samuilitsa II cave the entire sequence is of Levallois-Mousterian technology (33), as in the lower part is more of Levallois technology with Mousterian tools, and in the upper part of the sequence are more IUP prismatic cores and UP tools types (8) (34). An old 14C date from the middle part of Samuilitsa II cave sequence spans from 48,420 to 43,280 calBP (GrN- 5181 in (35) and place the sequence within the IUP assemblages or even a little earlier **(S11 Figure)**.

Toplitsa cave, located in a neighbor valley of Samuilitsa II (36) have delivered small lithic assemblage in Layer 5 technologically consistent with IUP or other transitional MP-UP assemblages(37). Two *Ursus spelaeus* teeth from the underlying Layer 5 have been radiocarbon dated to 49,770 – 45,750 cal BP (95.4% probability) and to 48,210 – 45,050 cal BP (95.4% probability) (37).

The unique open-air site of Muselievo, located 8 km to the South from the Danube River and in the area of the Upper Campanian flint whose material of Bacho Kiro Cave finds its origin, is dated between 60-45 Ka BP and belonging to the first half of MIS 3 (38, 39). The site is attributed to final MP and is interpreted as a leaf point workshop located in the vicinity of a flint outcrop. A large inventory of over 500 leaf points and huge amounts of debitage flakes from the production have been excavated in single redeposited layer in the 1970s (38). This assemblage probably precedes the IUP from Bacho Kiro Cave but there is evidence that leaf points identical with those from Muselievo site were found in the middle part of Samuilitsa II sequence (33, 39)suggesting mobility and connexons of the bifacial leaf points producers ne with the Levallois-Mousterian entities from Samulitsa II cave.

**References**

1. Talamo S, Kromer B, Richards MP, Wacker L. Back to the future: The advantage of studying key events in human evolution using a new high resolution radiocarbon method. PLoS ONE. 2023; 18(2): e0280598.

2. Folk RL. Practical Petrographic Classification of Limestones1. AAPG Bulletin. 1959;43(1):1-38.

3. Delvigne V, Fernandes P, Tuffery C, Raynal J-P, Klaric L. Taphonomic methods and a database to establish the origin of sedimentary silicified rocks from the Middle-recent Gravettian open-air site of La Picardie (Indre-et-Loire, France). Journal of Archaeological Science: Reports. 2020;32:102369.

4. Přichystal A. CLassification of lithic raw materials used for prehistoric chipped artefacts in general and siliceous sediments (silicites) in particular: The Czech proposal. Archeometriai Muhely. 2010;7:177-82.

5. Knauth LP. Petrogenesis of chert. Reviews in Mineralogy and Geochemistry. 1994;29(1):233-58.

6. Fernandes P, Delvigne V, Piboule M, Raynal JP. “I've been havin' some hard travellin*…". The evolutionary chain: a petrological concept for the reconstruction of litho-spaces. . Journal of archaeological method and theory. (under press).

7. Delvigne V, Fernandes P, Bindon P, Bracco J-P, Klaric L, Lafarge A, et al. Geo-resources and techno-cultural expressions in the south of the French Massif Central during the Upper Palaeolithic: determinism and choices. 2019.

8. Delvigne V, Fernandes P, Noiret P. Quand la pétroarchéologie questionne la notion de site : états de surface et taphonomie des objets lithiques de La Belle-Roche (Sprimont, Prov. de Liège, Belgique). Comptes Rendus Palevol. 2021.

9. Folk RL, Ham WE. Spectral Subdivision of Limestone Types1. Classification of Carbonate Rocks—A Symposium. 1: American Association of Petroleum Geologists; 1962. p. 0.

10. Delvigne V, Fernandes P, Piboule M, Lafarge A, Raynal J-P. Circulation de géomatières sur de longues distances au Paléolithique supérieur : le cas des silex du Turonien du Sud du Bassin parisien. Comptes Rendus Palevol. 2017;16(1):82-102.

11. Tomasso A, Binder D, Fernandes P, Milot J, Lea VR. The Urgonian chert from Provence (France): the intra-formation variability and its exploitation in petro-archeological investigations. Archaeological and Anthropological Sciences. 2017;11:253-69.

12. Krumbein WC, Sloss LL. Stratigraphy and sedimentation: Gilluly, J. & Woodford, A.O.; 1955.

13. Shepard FP, Young R. Distinguishing betyween beach and dune sands. . Journal of Sedimentary Petrology. 1961;31:196-214.

14. Arbey F. Les formes de la silice et l’identification des évaporites dans les formations silicifiées. . Bulletin du Centre Recherche Exploration Production Elf-Aquitaine. 1980;4:308-65.

15. Rio M. Les accidents siliceux dans le Crétacé du bassin vocontien (Sud-Est de la France). Contribution à l’étude de la silicification des formations calcaires1982.

16. Bullock P., Fedoroff N., Jongerius A., Stoops G., Tursina T, Babel U. Handbook for Soil Thin Section Description. 152 pp. . 2009/05/01 ed: Cambridge University Press; 1985.

17. Boulvain F. Éléments de sédimentologie et de pétrologie sédimentaire. from ulg.ac.be/geolsed/sedim/dedimentologie.html.: ; 2011 [

18. Le Ribault L. L'exoscopie méthode et application. : Paris : Compagnie Francaise des Petroles; 1975.

19. Fernandes P, Raynal J-P. Pétroarchéologie du silex : un retour aux sources. Comptes Rendus Palevol. 2006;5:829-37.

20. Fernandes P, Le Bourdonnec F-X, Raynal J-P, Poupeau G, Piboule M, Moncel M-H. Origins of prehistoric flints: the neocortex memory revealed by Scanning Electron Microscopy. Comptes Rendus Palevol. 2007;6:557-68.

21. Thiry M, Fernandes P, Milnes A, Raynal J-P. Driving forces for the weathering and alteration of silica in the regolith: Implications for studies of prehistoric flint tools. Earth-Science Reviews. 2014;136:141-54.

22. Inizan ML, Reduron-Balliger M, Roche H, Tixier J. Technologie de la pierre taillée. Préhistoire de la pierre taillée. Meudon.: CREP; 1995.

23. Pelegrin J. Les techniques de débitage laminaire au Tardiglaciaire : critères de diagnose et quelques réflexions. In: Valentin B, Bodu P, Christensen M, editors. L‘Europe centrale et septentrionale au Tardiglaciaire. Actes de la table-ronde de Nemours, 1997.: Mémoires du Musée de Préhistoire d‘Ile de France; 2000. p. 73-86.

24. Inizan ML, Reduron-Ballinger M, Roche H, Tixier J. Technology and Terminology of Knapped Stone1999.

25. Bon F. L’Aurignacien entre Mer et Océan. Réflexion sur l’Unité des Phases Anciennes de l’Aurignacien dans le Sud de la France. Paris2002.

26. Zwyns N. Laminar technology and the onset of the Upper Paleolithic in the Altai, Siberia 2012.

27. Tixier J. Typologie de l'épipaléolithique du Maghreb. Paris: Arts et métiers graphiques; 1963.

28. Tsanova T, Zwyns N, Eizenberg L, Teyssandier N, Le Brun-Ricalens F, Otte M. Le plus petit dénominateur commun : réflexion sur La variabilité des ensembles lamellaires du paléolithique supérieur ancien d’Eurasie. Un bilan autour des exemples de Kozarnika (Est des Balkans) et Yafteh (Zagros Central). L'Anthropologie 2012;116.4 469–509.

29. Brun-Ricalens F. Les pièces esquillées: État des connaissances après un siècle de reconnaissance. Paléo. 2006;18.

30. Horta P, Cascalheira J, Bicho N. The Role of Lithic Bipolar Technology in Western Iberia’s Upper Paleolithic: the Case of Vale Boi (Southern Portugal). Journal of Paleolithic Archaeology. 2019;2.

31. Bordes F, Vaufrey R. Typologie du Paléolithique ancien et moyen. Bordeaux: Impr. Delmas; 1961.

32. Sisk M. European Upper Paleolithic Stone Tool Typology: An annotated translation of the de Sonneville-Bordes and Perrot typology. 2013.

33. Sirakov N. Reconstruction of the Middle Palaeolithic flint assemblages from the Cave Samuilitsa II (northern Bulgaria) and their taxonomical position seen against the Palaeolithic of south-eastern Europe: Wrocław : Zakład narodowy im. Ossolińskich; 1983.

34. Tsanova T. A diachronic view of flake production from the beginning of the Upper Palaeolithic in the eastern Balkans. In: Pastoor A, Peresani M, editors. Flakes Not Blades: the Role of Flake Production at the Onset of the Upper Palaeolithic in Europe. 5. Mettman: Wissenschaftliche Schriftendes Neanderthal Museums; 2012. p. 215-38.

35. Tsanova T. Preliminary Comparison and Chronology of the Lithic Blade and Bladelet Assemblages at the Onset of the Upper Palaeolithic from Bacho Kiro, Temnata and Kozarnika Caves in the Eastern Balkans (Bulgaria). In: Ruis-Redondo ADW, editor. Proceedings of the British Academy. 258. Cambridge: The British Academy 2023. p. 156-202.

36. Taneva S, Chukalev K, Dimitrova I, Ganetsovski G, Gyaurova B, Popov P, et al. Test excavation and initial results of Toplica Cave, near Kunino village, region Vratsa. Annual of the National Archeological Museum, Sofia submitted.

37. Tsanova T, Veres D, Hambach U, Spasov R, Dimitrova I, Popov P, et al. Upper Palaeolithic layers and Campanian Ignimbrite/Y-5 tephra in Toplitsa cave, Northern Bulgaria. Journal of Archaeological Science: Reports. 2021;37:102912.

38. Sirakova S. The leafpoints of Muselievo. In: Kozlowski JK, editor. Les industries à pointes foliacées du Paléolithique supérieur européen. 42. Liège: ERAUL; 1990. p. 63-78.

39. Sirakova S. Middle Palaeolithic leaf points from Bulgaria and their analogues in Southeast and Central Europe. Sofia: Kastella M Company Ltd.; 2020.
