# Supplementary material for: Curated character of the Initial Upper Palaeolithic lithic artefact assemblages in Bacho Kiro Cave (Bulgaria)
Source: PLoS One. 2024 Sep 4;19(9):e0307435. doi: 10.1371/journal.pone.0307435 (PMC11373871; doi:10.1371/journal.pone.0307435)
Supplement: Supplementary file 12 [file pone.0307435.s008.docx]

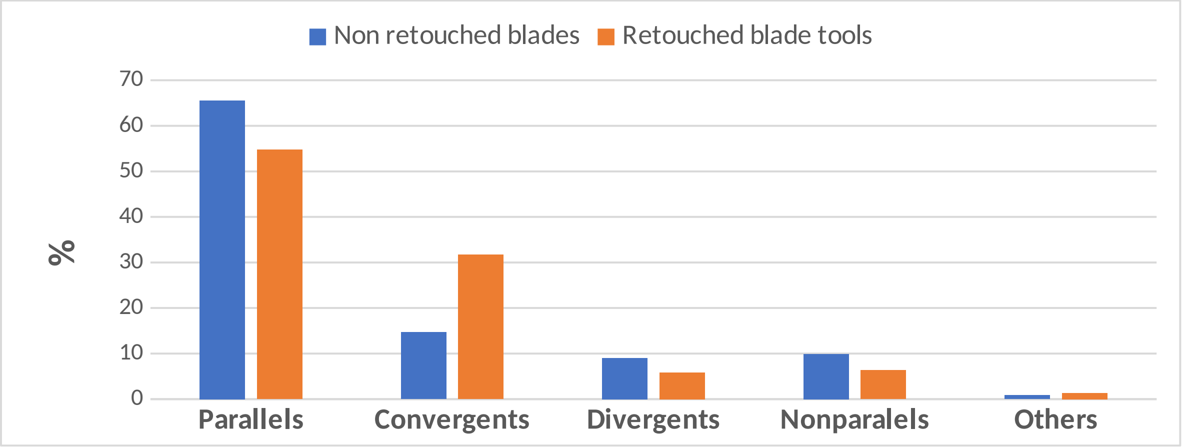
Non retouched blades
Retouched blade tools
%
70
60
50
40
30
20
10
0
Parallels
Convergents
Divergents
Nonparalels
Others
