# Supplementary material for: Curated character of the Initial Upper Palaeolithic lithic artefact assemblages in Bacho Kiro Cave (Bulgaria)
Source: PLoS One. 2024 Sep 4;19(9):e0307435. doi: 10.1371/journal.pone.0307435 (PMC11373871; doi:10.1371/journal.pone.0307435)
Supplement: Supplementary file 13 [file pone.0307435.s014.docx]

	Lower Cretaceous flints						
Geotope	Ludogoria				Ravno-Kamenovo ara		
Type	Group 11 - Ludogorie	Group 12 - Ludogorie sl	Group 13 - Tsenovo	Group 16 - Ludogorie (Lyublen)	Group 22 - Ravno	Group 23 - Ravno / Kamenovo	Group 24 - Ravno
Presence IUP	n = 587	n = 1	n = 203	n = 3	n = 3	n = 0	n = 1
Planche	Plate 1 (n°1-6)	none	plate 2 (n°1-7)	Plate 1 (n°7)	plate 3 (n°3-4)	plate 3 (n°5-6)	plate 3 (n°7-8)
Primary stratigraphic origin	Low Cretaceous (Aptian)	Low Cretaceous (Aptian)	Low Cretaceous (Aptian)	Low Cretaceous (Aptian)	Low Cretaceous (Barremo-Aptian)	Low Cretaceous (Barremo-Aptian)	Low Cretaceous (Barremo-Aptian)
Type of silicite	marine flint of internal plateform	marine flint of internal plateform	marine chert of internal plateform	marine flint of internal plateform	marine flint of external plateform	marine flint of external plateform	marine flint of external plateform
Type of host rock	Biocalcarenite	Biocalcarenite	Biocalcarenite	Biocalcarenite	calcimicrite	calcimicrite	calcimicrite
Habitus	decimetric to pluridecimetric regular nodule	indeterminate	bench fragments	decimetric to pluridecimetric regular nodule	decimetric to pluridecimetric regular nodule	decimetric to pluridecimetric regular nodule	decimetric to pluridecimetric regular nodule
primary color(s)	grey	black	black	gry	undetermined	grey	x
acquired color(s)	blond to brown	brown	brown	brown	orange	blond to brown	brown
Porogenesis	interparticle (o) ; intraparticle (o) ; vuggy (--) ; fracture (--)	interparticle (-) ; intraparticle (-)	interparticle (-) ; intraparticle (-)	interparticle (+) ; intraparticle (o)	x	intraparticle (-)	x
Poronecrosis	clogged with clear silica (+) ; lined by automorphic silica (-) ; left empty (--)	clogged with clear silica (o) ; left empty (--)	clogged with clear silica (o) ; left empty (--)	clogged with clear silica (o) ; left empty (--)	x	lined by automorphic silica or clogged by FeO	x
Structure	homogeneous	homogeneous (rarely bioturbate)	homogeneous (rarely bioturbate)	homogeneous (rarely bioturbate)	homogeneous	homogeneous	honogeneous
Allochems (abundance) (%det ; %chi ; %bio)	30 to 40 % (10% ; 70 % ; 20 %)	≈40 % (10 % ; 70 % ; 20 %)	≈40 % (10 % ; 70 % ; 20 %)	≈40 % (10 % ; 10 % ; 80 %)	< 10% (80% ; 0% ; 20 %)	< 10% (80% ; 0% ; 20 %)	< 10% (80% ; 0% ; 20 %)
Sorting of clasts	moderate to bad	moderate to bad	moderate to bad	moderate	undetermined	goog	undetermined
Distribution of clasts	homogeneous	homogeneous	homogeneous	homogeneous	homogeneous	homogeneous	homogeneous
Type of allochems	multiple	multiple	multiple	multiple	detrital	detrital	detrital
Particular background	< 50 microns (o)	< 50 microns (o)	< 50 microns (o)	< 50 microns (o)	(+) < 50 microns	(+) < 50 microns	(+) < 50 microns
Detrital component	detrital quartz (o) ; glauconite (---) ; endoclast (o) ; initialy black clasts (+)	detrital quartz (o) ; endoclast (o)	detrital quartz (o) ; endoclast (o)	detrital quartz (+) ; endoclast (-)	detrital quartz (o) ; micas (---)	detrital quartz (o) ; micas (---)	detrital quartz (o)
Shape and average size of detrital clasts	150 to 200 microns ; sph. 0,7-arr. 0,9	< 400 microns ; sph. 0,7-arr. 0,9	< 400 microns ; sph. 0,7-arr. 0,9	< 400 microns ; sph. 0,7-arr. 0,9	< 150 microns ; sph. 0,9 ; arr. 0,7-0,9	< 150 microns ; sph. 0,7 ; arr. 0,5-0,7	< 150 microns ; sph. 0,7 ; arr. 0,5-0,7
Chemical component	pelloids (150 microns ; sp.0,7-0,9 ; arr.0,9) (++)	pellods (200 à 400 microns ; sp.0,7 ; arr.0,9) (++)	pellods (200 à 400 microns ; sp.0,7 ; arr.0,9) (++)	pellods (200 à 400 microns ; sp.0,7 ; arr.0,9) (-)	x	x	x
Algae	x	x	dasycladale (---)	x	x	x	x
Echinoids	interambulacral area and spines (--)	spines (o>+) plate (++)	crinoids (--) ; spines (--)	x	x	x	x
Bivalves	shell fragments (+)	shell fragments (+)	shell fragments (+)	shell fragments (o)	x	x	x
Gastropods	shell fragments (o)	x	shell fragments ? (-)	x	x	x	x
Sponges	demosponges : monaxons, tetraxons partialy fragmented (++)	x	demospongs : monaxons partialy fragmented (o)	demospongs : monaxons partialy fragmented (o)	hexactinnelids (?) : monaxone and toxes partialy fragmented (o)	hexactinnelids (?) : monaxons (o)	hexactinnelids (?) : monaxons, triaxons and fused (o)
Bryozoans	cyclostoma (-)	cyclostoma (--)	cyclostoma (o)	cyclostoma (--)	x	x	x
Benthic foraminifera	Sabaudia (o) ; Textularia (o) ; Glomospira (o) ; Rotalidea (-) ; Lituolidea (-) ; Triloculina ? (-)	non observed	Glomospira (o) ; Textularia (-) ; Rotalidea (--) ; Lituolidea (---)	Sabaudia (o) ; Textularia (o) ; Rotalidea (o) ; Lituolidea (o)	x	x	x
Planktonic foraminifera	x	x	x	x	x	Heterohelicidea (-); Globigerinidea (-)	x
Ostracoda	valve fragments (o)	x	valve fragments (-)	x	x	x	x
Organic material	(---)	(--)	(--)	x	x	x	x
Other(s)	Incertae sedis (o)	Incertae sedis (-)	Incertae sedis (o)	serpulid (-) : Incertae sedis (-)	x	x	x
number of lithics	587	1	203	3	3	0	1
%	56.9	0.09	19.7	0.2	0.2	0	0.09

	Upper Cretaceous flints			
Geotope	Shumen	Nikopol region		
Type	Group 61 - Shumen area	Group 33 - Nikopol - Asenovo	Group 34 - Ohoden	Group 35 - Muselievo - Somovits
Presence IUP	n = 8	n = 214	n = 4	n = 9
Planche	none	plate 4 (n°1-7)	plate 5 (n°1-2)	plate 5 (n°3-6)
Primary stratigraphic origin	Upper Cretaceous (Campanian)	Upper Cretaceous (Campanian)	Upper Cretaceous (Maastrichtian)	Upper Cretaceous (Campanian)
Type of silicite	marine flint of external plateform	marine flint of external plateform	silicified limestone	marine flint of (external ?) plateform
Type of host rock	biocalcimicrite	biocalcimicrite	biocalcimicrite	biocalcimicrite
Habitus	decimetric irregular nodules	decimetric to pluridecimetric regular nodule	unknown	decimetric to pluridecimetric regular nodule
primary color(s)	grey to black	grey to black	grey	grey to black
acquired color(s)	blond to brown	blond to brown	x	blond to brown
Porogenesis	intraparticle (-) ; vuggy (--)	intraparticle (o)	interparticle (o)	fracture (-) ; intraparticle (-)
Poronecrosis	clogged with clear silica or left empty	left empty or clogged by clear silica or FeO	left empty or clogged by clear silica	left empty, linned by automorphic silica or clogged by clear silica
Structure	homogeneous (rarely bioturbate)	homogeneous (rarely bioturbate)	homogeneous to bioturbate	homogeneous to bioturbate
Allochems (abundance) (%det ; %chi ; %bio)	≈15 % (80 % ; 0% ; 20%)	≈40% (60% ; 10% ; 40%)	≈40 % (70 % ; 0% ; 30 %)	≈30 % (60% ; 10% ; 30%)
Sorting of clasts	good	moderate	moderate to good	moderate
Distribution of clasts	homogeneous	homogeneous	homogeneous	homogeneous
Type of allochems	multiple	multiple	multiple	multiple
Particular background	(+) < 50 microns	(++) < 50 microns	(++) < 50 microns	(++) < 50 microns
Detrital component	detrital quartz (o) ; gauconite (---); endoclast (-) ; initialy black clasts (--)	detrital quartz (o > +) ; gauconite (---); endoclast (o) ; initialy black clasts (o)	detrital quartz (o) ; endoclast (-) ; initialy black clasts (-)	detrital quartz (-) ; endoclast (o)
Shape and average size of detrital clasts	< 100 microns ; sph. 0,7-0,9 ; arr. 0,7-0,9	≈100 microns ; sph. 0,7-0,9 ; arr. 0,9	< 200 microns ; sph. 0,5-0,7 ; arr. 0,9	< 150 microns ; sph. 0,7 ; arr. 0,9
Chemical component	x	pelloids (150 microns ; sp.0,7 ; arr.0,9) (---)	x	pelloids (200 microns ; sp.0,7 ; arr.0,9) (-)
Algae	x	dasycladale (---)	x	dasycladacée (o)
Echinoids	interambulacral area (---)	interambulacral area and spine (-) ; scleritis of holoturie (---)	x	spines (-)
Bivalves	x	shell fragments ? (-)	shell fragments ? (o)	shell fragments ? (o)
Gastropods	shell fragments (--)	shell fragments ? (-)	shell fragments ? (o)	shell fragments ? (o)
Sponges	hexactinnelids : monaxons, triaxons and fused (+)	hexactinnelids : monaxons, triaxons and fused (++)	hexactinnelids : monaxons, triaxons and fused (++)	hexactinnelids : monaxons, triaxons (++)
Bryozoans	cheilostoma and cyclostoma (---)	cyclostoma and cheilostoma (--)	x	cyclostoma and cheilostoma (+)
Benthic foraminifera	Rotalidae (-)	Textularidae (-) ; Rotalidae (-)	x	Rotalidea (o) ; Lenticulina (-)
Planktonic foraminifera	Heterohelicidea (-) ; Globorotalidea (o); Globotruncanidea (-) ; Guembelitria (---)	Heterohelicidea (o) ; Globorotalidea (-) Globotruncanidea (-) ; Guembelitria (---)	x	Heterohelicidea (o)
Ostracoda	(---)	(---)	x	x
Organic material	x	x	x	x
Other(s)	spumellar radiolarian (---)	spumellar radiolarian (+) ; worms tubes (---)	x	spumellar radiolarian (-)
number of lithics	8	214	4	9
%	0.7	20.7	0.3	0.8

	Upper Cretaceous flints						Hydrothermal silicia	
Geotope	Unknown origin						Rhodopes	
Type	Group 21 - unknown origin	Group 36 - unknown origin	Group 37 - unknown origin	Group 41 - unknown origin	Group 42 - unknown origin	Group 62 - unknown origin	Group 51 - Rhodopes	
Presence IUP	n = 0	n = 4	n = 0	n = 0	n = 0	n = 1	n = 0	
Planche	plate 3 (n°1-2)	plate 3 (n°7-8)	none	plate 6 (n°1-2)	plate 6 (n°3-6)	none	plate 6 (n°7-8)	
Primary stratigraphic origin	Upper Cretaceous	Upper Cretaceous	Upper Cretaceous	Upper Cretaceous	Upper Cretaceous	Upper Cretaceous (?)	Eo-Oligocene	
Type of silicite	marine flint of external plateform	marine flint of (external ?) plateform	marine flint of (external ?) plateform	silcrete of marine platform flints	marine flint of external plateform	marine flint of external plateform	volcanogenic silica	
Type of host rock	calcimicrite	biocalcimicrite	biocalcimicrite	undetermined	biocalcimicrite	biocalcimicrite	unknown	
Habitus	unknown (Danube valley ?)	unknown (Danube valley ?)	unknown (Shumen area?)	unknown (Danube valley ?)	unknown (Danube valley ?)	unknown (Danube valley ?)	unknown	
primary color(s)	grey to black	blue-grey	grey	grey	blue-grey	black	colorless	
acquired color(s)	borwn	blond	blond	x	x	brown	red	
Porogenesis	intraparticle (-)	x	intraparticle (o)	x	interparticle (o) ; fracture (o)	x	interparticle (o)	
Poronecrosis	left empty	x	left empty (-)	x	left empty or lined by automorphic microquartz	x	left empty or clogged by FeO	
Structure	homogeneous	homogeneous	homogeneous	heterogeneous	heterogeneous	heterogeneous	pseudobedded	
Allochems (abundance) (%det ; %chi ; %bio)	< 10 % (0% ; 0% ; 100%)	≈10% (50% ; 0% ; 50%)	≈10 % (50 % ; 0% ; 50%)	< 15% (80% ; 0% ; 20 %)	< 20 % (70% ; 0% ; 30%)	≈30 % (30% ; 0% ; 70%)	< 10% (100% ; 0% ; 0%)	
Sorting of clasts	undetermined	undetermined	indeterminate	moderate	undetermined	moderate	undetermined	
Distribution of clasts	homogeneous	homogeneous	homogeneous	heterogeneous	heterogeneous	homogeneous	undetermined	
Type of allochems	biogenic	multiple	biogenic	multiple	multiple	multiple	detrital	
Particular background	(o) < 100 microns	x	(+) < 50 microns	(o) < 50 microns	x	(+) < 50 microns	x	
Detrital component	x	detrital quartz (-) ; endoclast (o)	detrital quartz (-) ;endoclast (-)	detrital quartz (--) ; endoclast (o)	detrital quartz (o)	detrital quartz (-) ; endoclast (o)	detrital quartz (-)	
Shape and average size of detrital clasts	x	< 150 microns ; sph. 0,7 ; arr. 0,9	< 100 microns ; sph. 0,7-0,9 ; arr. 0,7-0,9	< 150 microns ; sph. 0,7 ; arr. 0,9	< 100 microns ; sph. 0,7 ; arr. 0,9	< 100 microns ; sph. 0,7 ; arr. 0,9	< 150 microns ; sph. 0,7 ; arr. 0,3-0,5	
Chemical component	x	x	x	x	x	x	x	
Algae	x	x	dasycladale (+)	x	x	x	x	
Echinoids	x	x	x	x	x	x	x	
Bivalves	x	x	x	x	shell fragments ? (-)	x	x	
Gastropods	shell fragments ? (o)	x	shell fragments (--)	x	shell fragments ? (-)	x	x	
Sponges	x	hexactinnelids : fused (+)	hexactinnelids : monaxons, triaxons and fused (o) and sycon (++)	hexactinnelids : monaxons (-)	hexactinnlids : monaxons, triaxons and fused (o)	hexactinnlids : monaxons, triaxons (+++)	x	
Bryozoans	cyclostoma (--)	x	cheilostoma and cyclostoma (---)	x	x	x	x	
Benthic foraminifera	Buliminidea (+)	x	Rotalidae (-)	Trochospirale indet. (---)	x	x	x	
Planktonic foraminifera	x	x	Heterohelicidea (-)	x	x	x	x	
Ostracoda	x	x	x	x	x	x	x	
Organic material	x	x	x	x	x	x	x	
Other(s)	x	x	spumellar radiolarian (---)	illuviations figure	x	x	x	total:
number of lithics	0	4	0	0	0	1	0	1038
%	0	0.3	0	0	0	0.09	0	100%
