# Supplementary material for: Curated character of the Initial Upper Palaeolithic lithic artefact assemblages in Bacho Kiro Cave (Bulgaria)
Source: PLoS One. 2024 Sep 4;19(9):e0307435. doi: 10.1371/journal.pone.0307435 (PMC11373871; doi:10.1371/journal.pone.0307435)
Supplement: Supplementary file 14 [file pone.0307435.s015.docx]

Gitologic Group (silicites)	number
**Group 11**	**598**
Close Secondary	118
Distant Secondary	109
indeterminate	319
Subprimary	52
**Group 12**	**1**
Distant Secondary	1
**Group 13**	**208**
Close Secondary	29
Distant Secondary	45
indeterminate	108
Primary	1
Subprimary	25
**Group 16**	**3**
Distant Secondary	1
indeterminate	2
**Group 21**	**1**
indeterminate	1
**Group 22**	**10**
indeterminate	4
Subprimary	6
**Group 23**	**2**
Close Secondary	1
indeterminate	1
**Group 24**	**1**
Distant Secondary	1
**Group 33**	**251**
Close Secondary	30
Distant Secondary	17
indeterminate	174
Primary	1
Subprimary	29
**Group 34**	**5**
indeterminate	5
**Group 35**	**17**
Close Secondary	3
indeterminate	13
Subprimary	1
**Group 36**	**4**
Distant Secondary	2
indeterminate	2
**Group 37**	**2**
Distant Secondary	2
**Group 41**	**1**
Primary	1
**Group 42**	**1**
Primary	1
**Group 51**	**1**
indeterminate	1
**Group 61**	**43**
Close Secondary	3
Distant Secondary	2
indeterminate	31
Primary	1
Subprimary	6
**Grup 62**	**1**
Distant Secondary	1
**Aptian sl flint**	**41**
Close Secondary	1
Distant Secondary	1
indeterminate	36
Subprimary	3
**Upper Cretaceous sl flint**	**86**
Distant Secondary	1
indeterminate	84
Subprimary	1

Other Rocks	number
**Basalt**	**5**
Distant Secondary	3
indeterminate	2
**Calcite**	**1**
indeterminate	1
**carbonated sandstone**	**8**
Distant Secondary	1
indeterminate	7
**iron oxyde**	**3**
indeterminate	3
**iron sandstone**	**1**
Subprimary	1
**Limestone**	**2**
Distant Secondary	1
indeterminate	1
**metarhyolith**	**2**
Distant Secondary	1
indeterminate	1
**psammite**	**3**
Distant Secondary	1
indeterminate	2
**Quartz**	**6**
Distant Secondary	2
indeterminate	4
**quartzitique sandstone**	**14**
Distant Secondary	1
indeterminate	12
Subprimary	1
**Rhyolithe**	**4**
Distant Secondary	2
indeterminate	2
**rhyolithe porphyrique**	**29**
Distant Secondary	19
indeterminate	10
**Sandstone**	**2**
Distant Secondary	2
**indeterminate**	**18**
indeterminate	18
